# Rapid pseudo five-component synthesis of intensively blue luminescent 2,5-di(hetero)arylfurans via a Sonogashira–Glaser cyclization sequence

**DOI:** 10.3762/bjoc.10.60

**Published:** 2014-03-18

**Authors:** Fabian Klukas, Alexander Grunwald, Franziska Menschel, Thomas J J Müller

**Affiliations:** 1 Heinrich-Heine Universität Düsseldorf, Institut für Organische Chemie und Makromolekulare Chemie, Universitätsstraße 1, D-40225 Düsseldorf, Germany

**Keywords:** C–C coupling, copper, DFT, fluorescence, furans, mircowave-assisted synthesis, multicomponent reactions, palladium

## Abstract

2,5-Di(hetero)arylfurans are readily accessible in a pseudo five-component reaction via a Sonogashira–Glaser coupling sequence followed by a superbase-mediated (KOH/DMSO) cyclization in a consecutive one-pot fashion. Besides the straightforward synthesis of natural products and biologically active molecules all representatives are particularly interesting due to their bright blue luminescence with remarkably high quantum yields. The electronic structure of the title compounds is additionally studied with DFT computations.

## Introduction

Multicomponent reactions (MCRs) [1–5] are conceptually diversity-oriented syntheses (DOS) [6–7] and have been developed to powerful tools for exploring broad ranges of different structural and functional characteristics. In addition, MCRs address the very fundamental principles of reaction efficiency and atom economy. Besides lead finding in pharmaceutical and medicinal chemistry [8–10] MCRs have also been recognized as a DOS tool for approaching functional π-systems [11] such as luminescent chromophores [6].

Interestingly, multicomponent syntheses of symmetrically substituted furans have remained rare [12–13], although furans are ubiquitous in nature [14]. In particular, 2,5-di(hetero)arylfurans are structural units with pronounced biological activities [15] and besides in natural products [16–17] they are also present in potential pharmaceuticals for the treatment of human African trypanosomiasis [18–20], and against human renal cancer cells [21–23]. Furthermore, 2,5-di(hetero)arylfurans have been reported as photonic chromophores [24]. However, the electronic and photophysical properties of 2,5-di(hetero)aryl furans have only been occasionally studied [25–27].

The classical synthesis of symmetrical 2,5-disubstituted furans proceeds via Paal–Knorr synthesis [28]. Due to sophisticated starting materials this very general pathway is often not suitable for a DOS approach. In addition, some starting materials are either not readily available or quite expensive. 2,5-dihalogenated furans can be in principle employed in cross-coupling reactions, however, the poor stability of these dihalogenated precursors renders this approach very tedious [29]. Recent publications report gold-catalyzed syntheses of di(hetero)arylfurans starting from arylbutadiynes [30–31]. However, the major drawback of this approach is the complex, time-consuming preparation of the complicated gold catalyst and the separate synthesis of the butadiyne substrates. In a similar study arylbutadiynes prepared by Glaser homocoupling were converted into symmetrical 2,5-di(hetero)arylfurans [32] employing the superbase system DMSO/KOH/H_2_O in the terminal cyclization step [33]. The same approach was applied to butadiynes that were formed by oxidative dimerization of arylalkynes with a Cu/Fe catalyst [34]. Apart from using reactive terminal alkynes as starting materials the major drawbacks of this approach are clearly the extended reaction times (3–6 d) and the removal of the catalyst after the coupling step.

Recently, we became particularly interested in sequentially Pd-catalyzed processes [35] starting from (hetero)aryl iodides [36]. In particular, the Pd–Cu-catalyzed Sonogashira–Glaser sequence represents a highly intriguing combination of a cross-coupling and an oxidation reaction in a one-pot fashion [37]. By this approach we can efficiently avoid the major disadvantage of starting from terminal alkynes, which are occasionally unstable and tend to undergo polymerization. Based upon the Sonogashira–Glaser sequence we recently presented a straightforward one-pot sequence for the synthesis of 2,5-di(hetero)arylthiophenes [38]. Here, we report the methodological development of a novel multicomponent synthesis of symmetrical 2,5-(hetero)arylfurans in the sense of a consecutive one-pot sequence. In addition, the photophysical and electronical properties are studied and the electronic structure is investigated by DFT calculations.

## Results and Discussion

### Synthesis

Prior to setting up the one-pot sequence we first optimized the conditions of the terminal cyclization step for the formation of 2,5-diphenylfuran (**2a**, Table 1) starting from 1,4-diphenylbutadiyne (**1a**) as a substrate. Just upon eyesight a remarkable luminescence of **2a** caught our attention and we were encouraged to perform photophysical studies with these products as well (vide infra). In a set of experiments the reaction times under microwave heating (the temperature optimum of 130 °C was quickly identified by screening experiments), the water/KOH ratio, and the concentrations were varied.

**Table 1:** Evaluation of different reaction conditions.

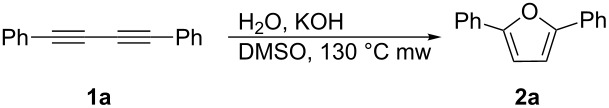					

Entry	H_2_O [equiv]	KOH [equiv]	DMSO [mL/mmol]	Time [h]	Yield^a^ [%]

1	2	2	4	1	53
2	2	2	4	3	24
3	10	2	4	1	25
4^b^	2	2	4	1	34
5^c^	2	2	4	1	36
6^c^	16	10	4	1	50
7^d^	8	10	4	1	40
8^d,e^	8	10	4	6	43
9	2	2	8	1	58
10	4	4	8	1	64
11	2	8	16	1	13
**12**	**8**	**8**	**16**	**1**	**84**
13	12	12	16	1	79

^a^Isolated yield after chromatography on silica gel. ^b^In the presence of 2 mol % PdCl_2_(PPh_3_)_2_. ^c^In the presence of 5 mol % CuI, 15 mol % DMEDA. ^d^In the presence of 5 mol % CuI, 15 mol % 1,10-phenanthroline. ^e^Conductive heating (oilbath at 130 °C).

In the course of our studies a related work using copper catalysts with the electronrich DMEDA (*N*,*N’*-dimethylethylenediamine) or the electronpoor 1,10-phenanthroline as ligands was published [32], however, in our hands no favorable effect on the isolated yields of **2a** was found (Table 1, entries 4–8). The cyclization works equally well with conductive heating in an oil bath instead of microwave heating (Table 1, entry 8). At higher concentrations we always observed the formation of byproducts that were not detectable by GC, although **1a** was completely consumed. An isolated black solid with an elemental analysis matching with the elemental composition of **2a** was partially soluble in acetone and THF. The supernatant of the THF extraction was analyzed by MALDI–TOF mass spectrometry indicating the formation of oligomers with *m*/*z* = 422 to 1046. We could efficiently suppress this oligo- and polymerization by increasing the amount of solvent, i.e. by dilution (Table 1, entries 9–13). Additionally increasing the concentration of KOH and water also proved to be beneficial (Table 1, entries 10–13). The optimal conditions for this cyclization are marked in entry 12 of Table 1.

**Scheme 1:**
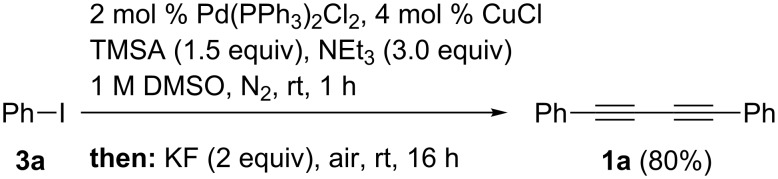
Sonogashira–Glaser sequence in DMSO as a solvent.

With these optimized conditions in hand we started to concatenate the one-pot sequence by generating the required 1,4-butadiynes from (hetero)aryl iodides. First, the Sonogashira–Glaser sequence had to be performed in DMSO as a solvent and in the presence of atmospheric oxygen for the Glaser step. Starting from iodobenzene (**3a**) and trimethylsilylacetylene (TMSA) the cross-coupling in DMSO proceeded uneventfully and the yield of the Glaser product **1a** was found to be 80%, i.e. approximately the same yield as for the sequence in THF as a solvent (Scheme 1) [37]. Most favorably no additional cosolvent was needed for increasing the solubility of the fluoride source [38]. For an optimal Glaser step vigorous stirring is required to ensure an efficient air saturation of the solvent.

**Scheme 2:**
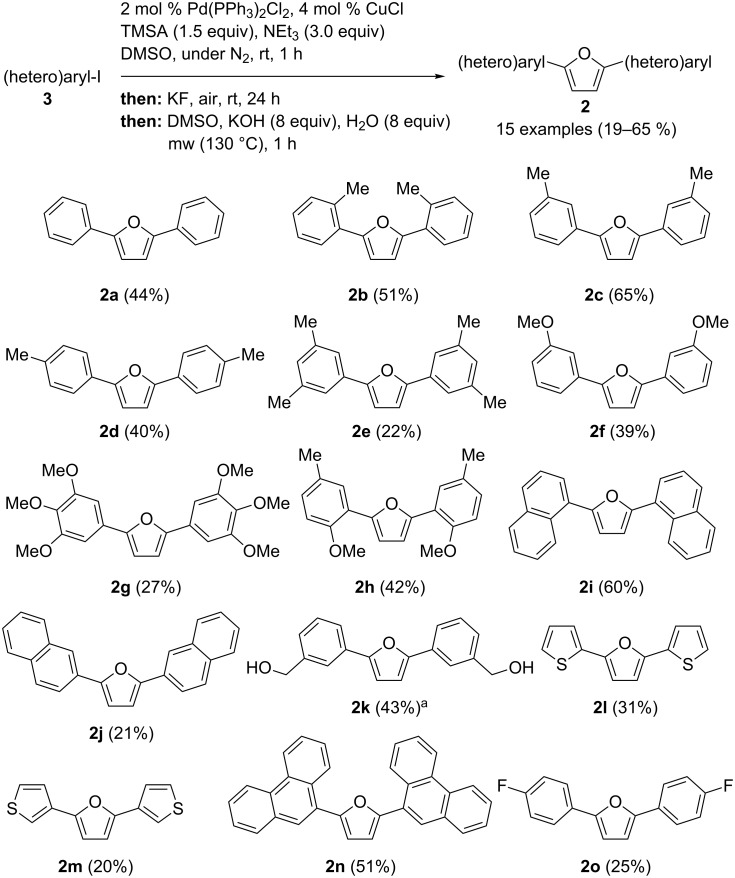
Pseudo five-component Sonogashira–Glaser cyclisation synthesis of 2,5-di(hetero)arylfurans **2** (^a^obtained from the THP-protected precursor).

Finally, starting from (hetero)aryl iodides **3** and TMSA we combined the Sonogashira–Glaser sequence with the cyclization step into a one-pot sequence and studied the substrate scope of this pseudo five-component synthesis of 2,5-di(hetero)arylfurans **2** (Scheme 2). All reactions were performed on a 2 mmol scale.

The structural assignments of all furans **2** were unambiguously supported by ^1^H and ^13^C NMR spectroscopy, mass spectrometry, and combustion analysis (HRMS in case of **2j** and **2m**). Due to the poor solubility of some compounds all spectra were recorded in DMSO at room temperature, whereas the compounds **2r** and **2p** were measured at 80 °C.

The yields of the obtained 2,5-di(hetero)arylfurans **2** are moderate to good and the employed (hetero)aryl substituents can be electroneutral (**2a**, **2i**, **2j**) and electronrich (**2b**–**2h**, **2k**, **2l, 2m**). Substituents in *ortho*- (**2b, 2h**), *meta*- (**2c**, **2e–2h**, **2k**) and *para*-position (**2d**, **2g**, **2o**) are well tolerated. Polar substituents like alcohols (**2k**) can also be employed in the sequence. From the literature it is known that the naturally occurring compound **2h** [16–17] and 2,5-bis(3,4,5-trimethoxyphenyl)furan (**2g**) are highly biological active [39]. Compound **2k** is structurally related to small molecule inhibitors of p53-HDM-2 [21–23].

### Electronic properties and computational studies

**Figure 1:**
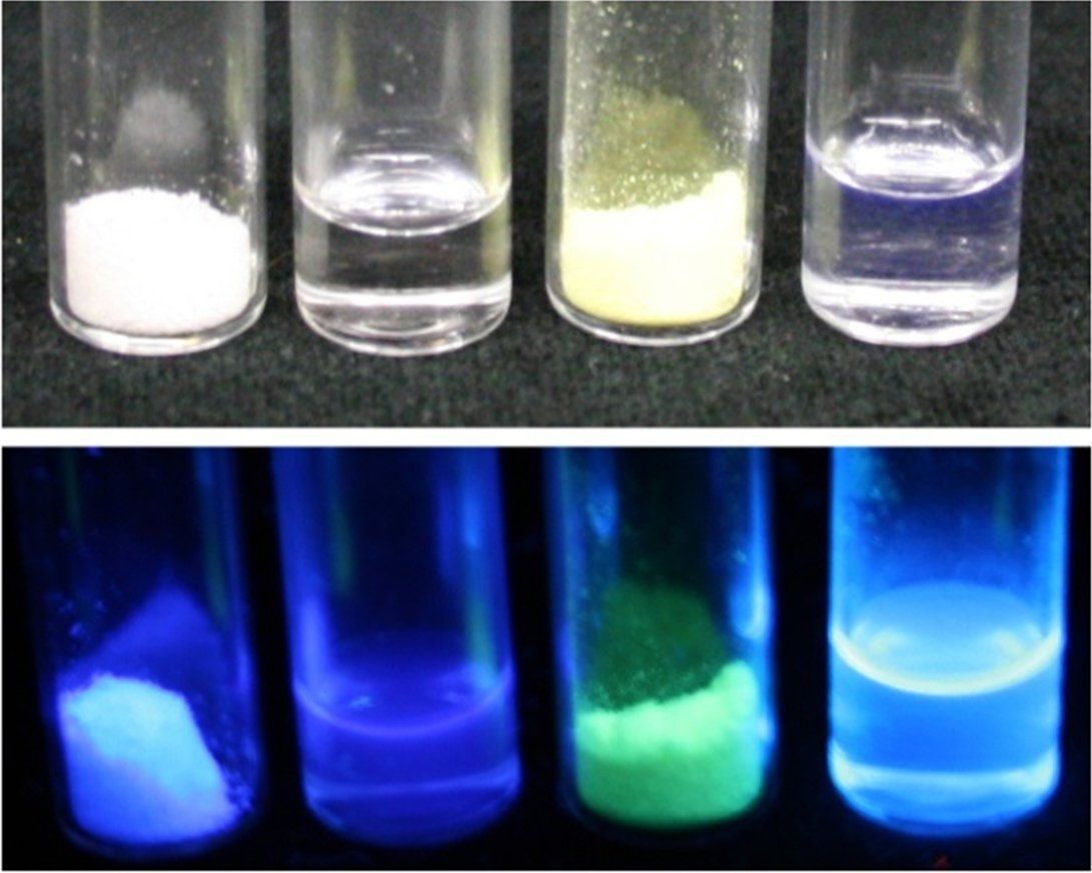
Compounds **2d** (solid and THF solution) and **2n** (solid and THF solution) (from left to right) under daylight (top) and under UV light (bottom, λ_max,exc_ = 366 nm).

All title compounds **2** display strong fluorescence in solution and in the solid state upon UV excitation (Figure 1). Therefore, the absorption and emission spectra of all compounds **2** were recorded in dichloromethane and the fluorescence quantum yields Φ_f_ were determined with coumarin 1 or *p*-terphenyl as references (Table 2).

**Table 2:** Selected absorption and emission data (recorded in dichloromethane at *T* = 293 K).

Compound	λ_max, abs_ [nm]^a^ ([L·mol·cm^−1^])	λ_max, em_ [nm]	Δ 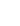 [cm^−1^]^b^	Φ_f_

				
**2a**	**327** (35000), 342 sh	358 sh, **373**	3800	83%^c^
**2b**	**314** (23000), 342 sh	359 sh, **375**	5200	59%^c^
**2c**	**329** (33000), 344 sh	360 sh ,**376**	3800	72%^c^
**2d**	**331** (33000), 348 sh	360 sh, **379**	3800	64%^c^
**2e**	**331** (28000), 347 sh	362 sh, **379**	3800	55%^c^
**2f**	**331** (29000), 347 sh	362 sh, **381**	4000	95%^c^
**2g**	**340** (33000), 356 sh	378 sh, **391**	3800	80%^c^
**2h**	**347** (33000), 364 sh	377 sh, **395**	3500	47%^c^
**2i**	**347** (29000)	424 sh, **436**	5900	75%^d^
**2j**	**358** (26000), 377 sh	393 sh, **411**	3600	100%^d^
**2k**	**329** (32000), 345 sh	362 sh, **377**	3900	80%^c^
**2l**	**353** (24000), 371 sh	389 sh, **407**	3800	42%^c,e^
**2m**	**321** (23000), 336 sh	351 sh, **367**	3900	29%^c^
**2n**	**343** (23000)	**439**	6400	69%^d^
**2o**	**323** (21000), 338 sh	353 sh, **368**	3800	76%^c^

^a^sh = shoulder. ^b^The boldfaced absorption and emission maxima were used to calculate the Stokes shifts. ^c^*p*-Terphenyl (Φ_f_ = 93% in cyclohexane) as a reference [40]. ^d^Coumarin 1 (Φ_f_ = 73% in EtOH) as a reference [41]. ^e^Ref. [26]: Φ_f_ = 33% in acetonitrile.

The most furans display intense, broad absorption bands between 321 and 358 nm with molar extinction coefficients between 21000 to 35000 L/mol cm^−1^. In addition redshifted shoulders appear between 336 and 377 nm. Likewise the emission maxima are found between 367 and 439 nm and blueshifted shoulders appear between 351 and 424 nm. The Stokes shifts 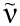 determined from the absorption and emission maxima range from 3500 to 6400 cm^−1^ and the quantum yields are quite large in a range from Φ_f_ = 29 to 100%. The compounds **2b**, **2i** and **2n** display unstructured broad absorption and emission bands and possess the largest Stokes shifts.

**Figure 2:**
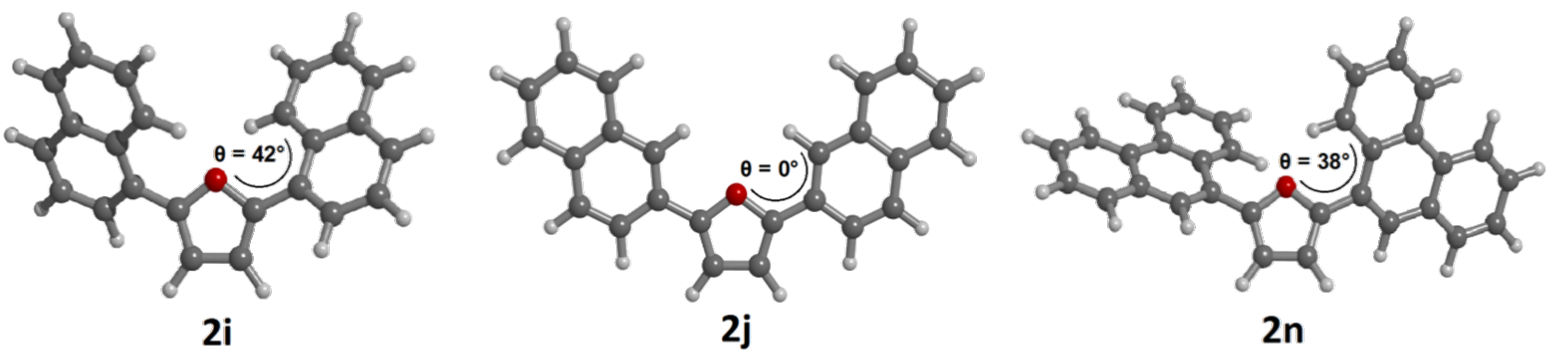
Selected computed minimum conformations of the 2,5-diarylfurans **2i**, **2j**, and **2n**.

This peculiar effect could arise from considerable geometrical differences between the electronic ground state and the vibrationally relaxed excited state caused by significant distortion of the aryl substituents from coplanarity in the ground state [42]. Therefore, the geometries of the ground state structures of the compounds **2a**,**2b**,**2i**,**2j,** and **2n** were optimized on the DFT level of theory (B3LYP functional [43–46] and the Pople 6-311G(d,p) basis set [47]) as implemented in Gaussian09 [48]. The computations applied the Polarizable Continuum Model (PCM) using dichloromethane as solvent [49]. All minima were confirmed by analytical frequency analyses. In conclusion, the computations clearly reveal that the *ortho*-aryl substituted compounds **2b**, **2h**, **2i** and **2n** are twisted from coplanarity while the other compounds are coplanar (Figure 2).

In the UV–vis spectra the similar planar structures **2a** and **2c** are bathochromically shifted in comparison to the twisted structure **2b**. The twisting from coplanarity also results in a lower fluorescence quantum yield Φ_f_. The same holds true for the comparison of the constitutional isomers **2i** and **2j**. Therefore, the twisted structure of **2j** causes a larger Stokes shift and a much lower fluorescence quantum yield Φ_f_. The huge Stokes shift originates from a considerable planarization in the excited state [42]. The absorption maximum of **2h** is considerably shifted bathochromically in comparison to those of **2c**, **2e**, and **2f**. The DFT calculation on structure **2h** reveals a twisted ground state structure. In the whole series compound **2j** shows the most redshifted absorption maximum and the highest fluorescence quantum yield Φ_f_. This finding correlates well with the planar ground state structure and an associated low Stokes shift. All studied representatives are potentially interesting singlet bluelight emitters.

In addition, the electronic properties of the furans **2** have been studied by cyclic voltammetry (Table 3). Most cyclovoltammograms display reversible Nernstian one-electron oxidations in the anodic region between 1.06 and 1.25 V (vs Ag/AgCl) (Table 3). Expectedly, with increasing electron density the oxidative potential diminishes. The compounds **2f**, **2g**, **2l**, and **2n** could not be measured by cyclic voltammetry due to precipitation on the electrode. The determined oxidation potentials *E*_1/2_^0/+1^ vs Ag/AgCl were recalculated vs the normal hydrogen electrode (NHE) and then transformed into eV [50]. The reduction potentials were calculated by subtraction of the *S*_1_–*S*_0_ energy gap (in eV) from the first oxidation potential *E*_1/2_^0/+1^. This gap was estimated by the cross-section of the absorption and emission spectra. For the missing oxidation potentials of **2f**, **2g**, **2m**, and **2l** the HOMO and LUMO energies were determined by DFT calculations [48]. For validation of the experimental and computational data the oxidation and reduction potentials were converted into the corresponding experimental HOMO and LUMO energies (for details see Supporting Information File 1). The plot of measured and calculated HOMO energies gives a reasonable linear correlation (*r*^2^ = 0.815, omitting the twisted compounds **2b**, **2h**, **2i** and **2n**) with a mean deviation of 0.05 eV (see Supporting Information File 1). Roughly a similar trend can be found for the HOMO–LUMO gap.

**Table 3:** Selected cyclovoltammetric^a^ (recorded in dichloromethane at 293 K) and computational^b^ data.

Compound	*E*_0_^0/+1^ [V]^c^	*E*_1/2_^0/+1^ [V] vs NHE^c^	*E*_1/2_^0/-1^ [V] vs NHE^d^	HOMO [eV]		LUMO [eV]		Δ*E*_HOMO−LUMO_	
				exp.^e^	calcd.^d^	exp.^f^	calcd.^b^	exp.	calcd.^b^

**2a**	1.25	1.45	−2.11	−5.60	−5.57	−2.04	−1.60	3.56	3.97
**2b**	1.19	1.39	−2.18	−5.54	−5.59	−1.97	−1.51	3.57	4.08
**2c**	1.19	1.39	−2.15	−5.54	−5.50	−2.00	−1.57	3.54	3.93
**2d**	1.09	1.29	−2.22	−5.44	−5.41	−1.93	−1.49	3.51	3.92
**2e**	1.06	1.26	−2.24	−5.41	−5.46	−1.91	−1.51	3.50	3.95
**2f**	−	−	−	−	−5.55	−	−1.57	−	3.98
**2g**	−	−	−	−	−5.44	−	−1.58	−	3.86
**2h**	1.24	1.44	−2.22	−5.59	−5.41	−1.93	−1.16	3.66	4.25
**2i**	1.15	1.35	−1.81	−5.50	−5.54	−2.34	−1.81	3.16	3.73
**2j**	1.10	1.30	−1.94	−5.45	−5.46	−2.21	−1.93	3.24	3.53
**2k**	1.16	1.36	−2.17	−5.51	−5.53	−1.98	−1.56	3.53	3.97
**2l**	−	−	−	−	−5.37	−	−1.70	−	3.67
**2m**	−	−	−	−	−5.44	−	−1.38	−	4.06
**2n**	1.14	1.34	−1.82	−5.49	−5.59	−2.33	−1.81	3.16	3.78
**2o**	1.24	1.44	−2.17	−5.59	−5.57	−1.98	−1.60	3.61	3.97

^a^ 0.1 M electrolyte: [Bu_4_N][PF_6_] (120 mg in 3 mL dichloromethane), Pt working electrode, Pt counter electrode, Ag/AgCl (in KCl) reference electrode. ^b^Calculated with Gaussian09, B3LYP/6-311G(d,p). ^c^

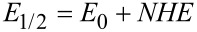
 (with NHE (3 M KCl Ag/Ag^+^) = 0.198 V). ^d^

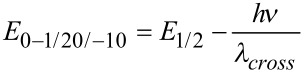
 (with cross-section of absorption and emission spectra). ^e^

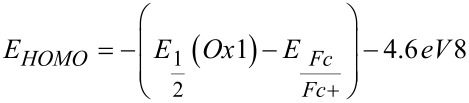
. ^f^

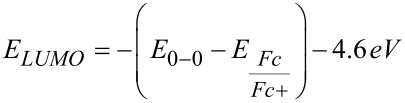
.

**Figure 3:**
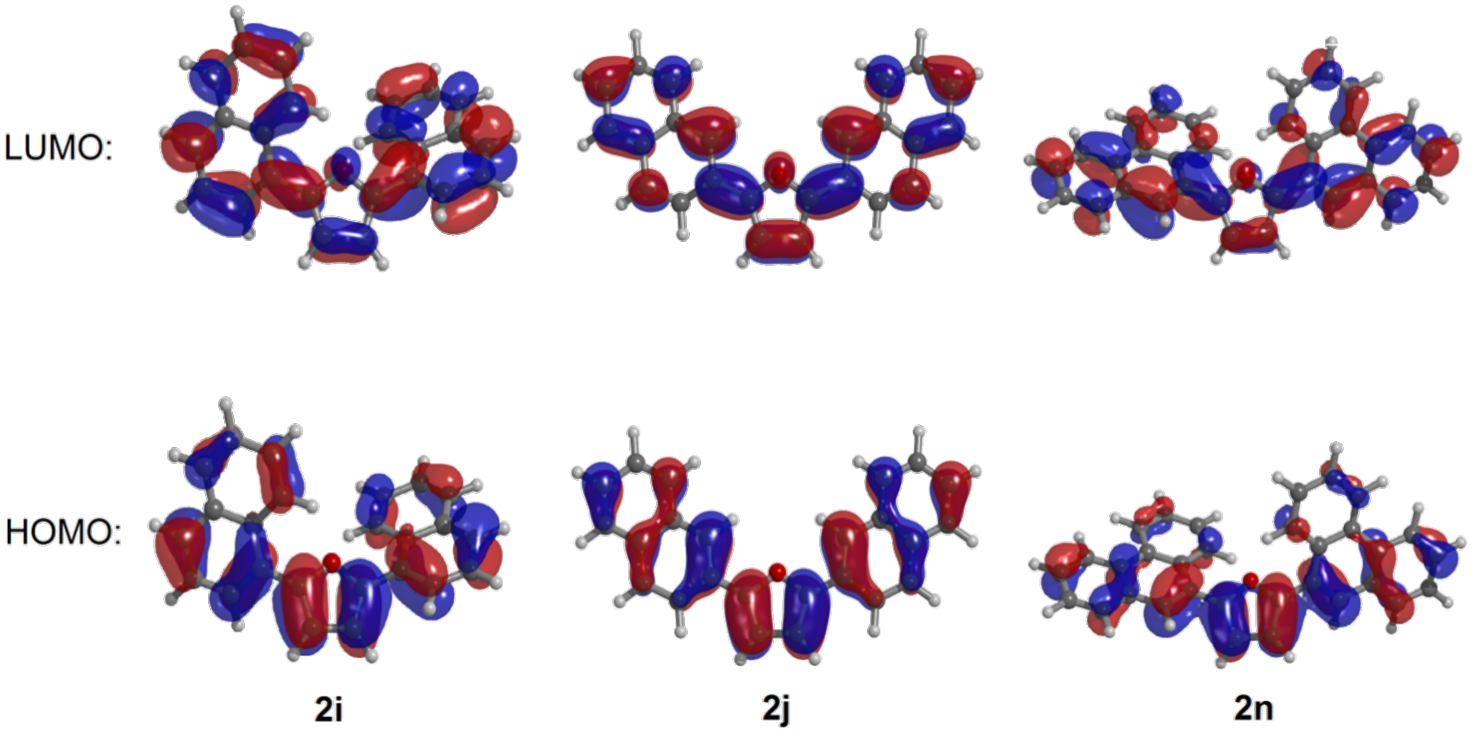
Kohn–Sham HOMOs (bottom) and LUMOs (top) of the compounds **2i**, **2j**, and **2n** (calculated on the DFT level of theory (B3LYP/6-311G(d,p)).

The inspection of the coefficient densities in the Kohn–Sham frontier molecular orbitals of the compounds **2i**, **2j**, and **2n** underlines that the HOMO and the LUMO are delocalized over the whole molecule (Figure 3), which plausibly rationalizes the high extinction coefficient of the longest wavelength absorptions bands.

## Conclusion

In summary we have disclosed a concise and efficient microwave-assisted pseudo five-component synthesis of symmetrical 2,5-di(hetero)arylfurans in a one-pot fashion, which opens a ready access to biologically active furan derivatives. In addition the investigation of the photophysical properties of these compounds reveals an intense blue luminescence in solution approaching unity for the fluorescence quantum yield Φ_f_ of distinct derivatives. Computations account for significant distortions from coplanarity in the electronic ground state and the computed HOMO energies correlate with the first reversible oxidation potentials determined by cyclic voltammetry.

## Experimental

**Pseudo five-component synthesis of 2a** [51] **in a manner similar to** [38]**):** A mixture of iodobenzene (**3a**, 408 mg, 2.00 mmol), PdCl_2_(PPh_3_)_2_ (28.1 mg, 0.04 mmol, 2 mol %), and CuCl (7.92 mg, 0.08 mmol, 4 mol %) was dissolved in DMSO (2.00 mL) in a 80 mL microwave vessel equipped with a stirring bar and a septum and was degassed with N_2_ for 5 min. After addition of trimethylsilylacetylene (0.42 mL, 3.00 mmol) and dry triethylamine (0.55 mL, 4.00 mmol) the solution was stirred at room temperature for 1 h. Then, KF (232 mg, 4.00 mmol) was added and the reaction mixture was vigorously stirred under air in the open reaction vessel at room temperature for 16 h. After the addition of H_2_O (144 mg, 8.00 mmol), potassium hydroxide (449 mg, 16 mmol), and DMSO (14.0 mL) the mixture was heated in the microwave cavity at 130 °C for 1 h. After cooling to room temperature the mixture was extracted with methylene chloride (300 mL) and brine (500 mL). The organic phase was dried with anhydrous Na_2_SO_4_ and the solvents were removed under reduced pressure. The residue was absorbed on Celite^®^ and purified by column chromatography on silica gel with *n*-hexane as an eluent to give 96.0 mg (0.44 mmol, 44%) of the desired product as colorless crystals. *R*_f_*:* = 0.31 (*n*-hexane). Mp 84 °C (66–68 °C [51]). ^1^H NMR (DMSO-*d*_6_, 600 MHz) δ 7.08 (s, 2H), 7.31 (t, *^3^J* = 7.4 Hz, 2H), 7.46 (t, *^3^J* = 7.8 Hz, 4H), 7.82 (d, *^3^J* = 7.3 Hz, 4H); ^13^C NMR (DMSO, 150 MHz) δ 108.2 (CH), 123.4 (CH), 127.5 (CH), 128.9 (CH), 130.0 (C_quat_), 152.6 (C_quat_). GC–MS (*m/z* (%)): 220 (M^+^, 100), 191 (13), 115 ((M-C_7_H_5_O)^+^, 41), 105 ((C_7_H_5_O)^+^, 22), 89 (14), 77 ((C_6_H_5_)^+^, 51), 63 (13), 51 (22); IR (KBr): 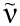 = 1479 (w) cm^−1^, 1446 (w), 1155 (w), 1022 (m), 925 (w), 910 (w), 794 (m), 756 (s), 689 (s), 671 (m). Anal. calcd for C_16_H_12_O (220.3): C 87.25, H 5.49; Found: C 87.09, H 5.42; UV–vis (CH_2_Cl_2_): λ_max_(ε): 327 nm (35000 L·mol^−1^·cm^−1^), 342 (22000). Fluorescence (CH_2_Cl_2_): λ_max_: 358 nm. Stokes shift Δ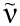 = 3800 cm^−1^. Quantum yield: Φ_f_ = 83% (Ref.: *p*-terphenyl (Φ_f_ = 93% in cyclohexane)). Cyclic voltammetry (CH_2_Cl_2_): *E*_1/2_^0/+1^ = 1.25 V.

## Supporting Information

For experimental details of the optimization studies of the cyclization step (compound **2a**), of general procedure of the Sonogashira–Glaser cyclization synthesis of the 2,5-di(hetero)arylfurans **2**, for UV–vis, fluorescence, and NMR spectra and cyclovoltammograms of the compounds **2**, and for computational data of the DFT calculations on the structures **2** of see Supporting Information.

File 1 Experimental procedures, spectroscopic and analytical data of all compounds **2**.
