# Supplementary material for: Rapid pseudo five-component synthesis of intensively blue luminescent 2,5-di(hetero)arylfurans via a Sonogashira–Glaser cyclization sequence
Source: Beilstein J Org Chem. 2014 Mar 18;10:672–9. doi: 10.3762/bjoc.10.60 (PMC3999768; doi:10.3762/bjoc.10.60)
Supplement: File 1 — Experimental procedures, spectroscopic and analytical data of all compounds 2. [file Beilstein_J_Org_Chem-10-672-s001.pdf]

Supporting Information

for

Rapid pseudo five-component synthesis of intensively blue luminescent 2,5-di(hetero)arylfurans via a Sonogashira–Glaser cyclization sequence

Fabian Klukas, Alexander Grunwald, Franziska Menschel, and Thomas J. J. Müller*

Address: Heinrich-Heine Universität Düsseldorf, Institut für Organische Chemie und Makromolekulare Chemie, Universitätsstraße 1, D-40225 Düsseldorf, Germany

Email: Thomas J.J.Müller - ThomasJJ.Mueller@uni-duesseldorf.de

*Corresponding author

Experimental procedures, spectroscopic and analytical data of all compounds 2.

Table of Contents

1. General considerations....s3
2. General procedure for optimization studies....s5
3. General procedure for the Sonogashira–Glaser cyclization sequence....s6
4. Analytical data of compounds **2a–2o**....s7
5. UV-absorption and fluorescence data of compounds **2a–2o**....s22
6. NMR spectra of compounds **2a–2o**....s30
7. CV data of compounds **2a–2o**....s60
8. xyz-Coordinates and dihedral angles of compounds **2a–2o**....s66

1. General considerations

All cross coupling reactions were carried out in oven-dried 80 mL microwave vessels (CEM Corporation) by using septa and syringes under nitrogen atmosphere. Commercial-grade reagents were used as supplied without further purification and were purchased from Sigma-Aldrich Chemie GmbH, ABCR GmbH & Co. KG, Alfa Aesar GmbH & Co. KG, and Merck Serono KGaA. The purification of products was performed on silica gel 60 (0.015–0.040 mm) from Macherey-Nagel Düren by using the flash technique under a pressure of 1.5 bar. The crude mixtures were adsorbed on Celite® 545 (0.02–0.10 mm) from Merck Serono KGaA Darmstadt before chromatographic purification. The reaction progress was monitored qualitatively by TLC silica gel 60 F254 aluminium sheets obtained by Merck Serono KGaA Darmstadt. The spots were detected with UV light at 254 nm and by using aqueous potassium permanganate solution.

^{1}H, ^{13}C, and 135-DEPT NMR spectra were recorded on a Bruker Avance III – 600 spectrometer. $CDCl_3$ and DMSO-d_6 were used as deuterated solvents. The resonances of the residues of nondeuterated solvent were locked as internal standards ($CDCl_3$: ^{1}H δ 7.26, ^{13}C δ 77.2; DMSO-d_6: ^{1}H δ 2.50, ^{13}C δ 39.52). The multiplicities of signals were abbreviated as follows: s: singlet; d: doublet; t: triplet; dd: doublet of doublets and m: multiplet. The type of carbon atoms was determined on the basis of 135-DEPT NMR spectra. EI mass spectra were measured on a Finnigan MAT 8200 or Shimadzu GC-2010/QP-2010 spectrometer. IR spectra were obtained on Bruker Vector 22 FTIR. The intensity of signals is abbreviated as follows: s (strong), m (medium) and w (weak). The melting points (uncorrected) were measured on a Büchi Melting Point B-540. Combustion analyses were carried out on Perkin Elmer Series II Analyser 2400 in the microanalytical laboratory of the Institut für Pharmazeutische and Medizinische Chemie at the Heinrich Heine-Universität Düsseldorf. The UV–vis spectra were recorded with a Perkin Elmer Spectrometer (Lambda 19). The emission data were carried out on Perkin Elmer Luminescence Spectrometer (LS55). The quantum yield was determined as followed:

$\boldsymbol{\phi_f}(\mathbf{sample}) = \boldsymbol{\phi_f}(\mathbf{reference}) \cdot \frac{F_{sample}}{F_{reference}} \cdot \frac{A_{reference}}{A_{sample}} \cdot \frac{n^2{}_{sample}}{n^2{}_{reference}}$ [1] with *p*-terphenyl (Φ_f = 93 % in cyclohexane) [2] or coumarin 1 (Φ_f = 73 % in EtOH) [3] as a reference.

Cyclic voltammetry experiments were performed with a 263A E&G Princeton Applied Research device under argon in dry and degassed dichloromethane at T = 293 K at scan rates of 100, 250, 500 and 1000 mVs^{-1}. The electrolyte was tetrabutylammonium hexafluorophosphate at a concentration of c = 0.1 mol L^{-1}. The working electrode was a 1 mm platinum disk, the counter electrode was a platinum wire and the reference electrode was a silver/silverchloride electrode filled with 3 M potassium chloride solution. The potentials were calibrated using $[FeCp_2]/[FeCp_2]^+$ ($E_0^{0/+1}$ = 450 mV) [4] as an internal potential standard. Computational support and infrastructure was provided by the "Center for Information and Media Technology" (ZIM) at the Heinrich-Heine-Universität Düsseldorf. The geometries of the ground state structures were optimizied on the DFT level of theory (B3LYP/6-311G(d,p) [5,6,7,8,9]) as implemented in Gaussian09 [10].

2. General procedure for optimization studies

A mixture of iodobenzene **3a** (101 mg, 0.50 mmol), H_2O (18.0 mg, 1.00 mmol) and potassium hydroxide (56.1 mg, 1 mmol) was dissolved in DMSO (2.00 mL) in a 10 mL microwave vessel equipped with a stirring bar and a septum. The mixture was heated in the microwave cavity at 130 °C for 1 h. After cooling to room temperature the mixture was extracted with methylene chloride (100 mL) and brine (250 mL). The organic phase was dried with anhydrous Na_2SO_4 and the solvents were removed under reduced pressure. The residue was absorbed on Celite® and purified by column chromatography on silica gel with *n*-hexane.

3. General procedure for the Sonogashira–Glaser cyclization sequence

In manner similar to [11]

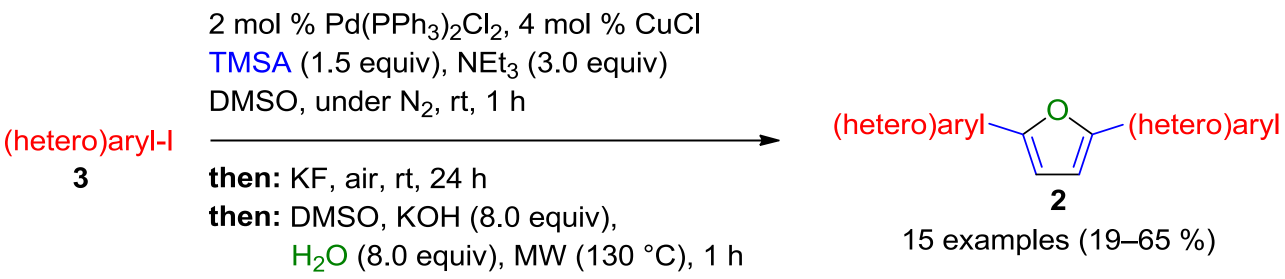

A mixture of a (hetero)aryl iodide **3** (2.00 mmol), $PdCl_2(PPh_3)_2$ (28.1 mg, 0.04 mmol, 2 mol %), and CuCl (7.92 mg, 0.08 mmol, 4 mol %) was dissolved in DMSO (2.00 mL) in a 80 mL microwave vessel equipped with a stirring bar and a septum and was degassed with N_2 for 5 min. After addition of trimethylsilylacetylene (0.42 mL, 3.00 mmol) and dry triethylamine (0.55 mL, 4.00 mmol) the solution was stirred at room temperature for 1 h. Then, KF (232 mg, 4.00 mmol) was added and the reaction mixture was vigorously stirred under air in the open reaction vessel at room temperature for 16 h. After the addition of H_2O (144 mg, 8.00 mmol), potassium hydroxide (449 mg, 16 mmol), and DMSO (14.0 mL) the mixture was heated in the microwave cavity at 130 °C for 1 h. After cooling to room temperature the mixture was extracted with methylene chloride (300 mL) and brine (500 mL). The organic phase was dried with anhydrous Na_2SO_4 and the solvents were removed under reduced pressure. The residue was absorbed on Celite® and purified by column chromatography on silica gel with *n*-hexane or *n*-hexane/THF as eluent.

4. Analytical data of compounds 2a–2o

2,5-Diphenylfuran (2a) [12]

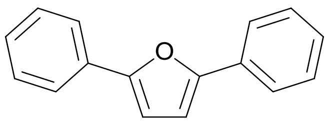

$C_{16}H_{12}O$
220.27

The synthesis was carried out according to the general procedure with 408 mg (2.00 mmol) of iodobenzene (**3a**) (Alfa Aesar). The crude product was absorbed onto Celite® and purified by column chromatography on silica gel with *n*-hexane as an eluent to give 96.0 mg (0.44 mmol, 44 %) of the desired product as colorless crystals.

R_f: = 0.31 (*n*-hexane). Mp 84 °C (66-68 °C [12]). ^{1}H NMR (DMSO-d_6, 600 MHz): δ= 7.08 (s, 2 H), 7.31 (t, ^3J = 7.4 Hz, 2 H), 7.46 (t, ^3J = 7.8 Hz, 4 H), 7.82 (d, ^3J = 7.3 Hz, 4 H). ^{13}C NMR (DMSO, 150 MHz): δ= 108.2 (CH), 123.4 (CH), 127.5 (CH), 128.9 (CH), 130.0 (C_{quat}), 152.6 (C_{quat}). GC-MS (*m/z* (%)): 220 (M^+, 100), 191 (13), 115 ($(M-C_7H_5O)^+$, 41), 105 ($(C_7H_5O)^+$, 22), 89 (14), 77 ($(C_6H_5)^+$, 51), 63 (13), 51 (22); IR (KBr): $\tilde{\nu}$ = 1479 (w) cm^{-1}, 1446 (w), 1155 (w), 1022 (m), 925 (w), 910 (w), 794 (m), 756 (s), 689 (s), 671 (m). Anal. calcd for $C_{16}H_{12}O$ (220.3): C 87.25, H 5.49; Found: C 87.09, H 5.42. UV–vis (CH_2Cl_2): $\lambda_{max}(\varepsilon)$: 327 nm (35000 L·mol^{-1}·cm^{-1}), 342 (22000). Fluorescence (CH_2Cl_2): λ_{max}: 358 nm. *Stokes* shift $\Delta\tilde{\nu}$ = 3800 cm^{-1}. Quantum yield: Φ_f = 83 % (ref.: *p*-terphenyl (Φ_f = 93 % in cyclohexane) [2]). Cyclic voltammetry (CH_2Cl_2): $E_{1/2}^{0/+1}$ = 1.25 V.

2,5-Di-*o*-tolylfuran (2b)

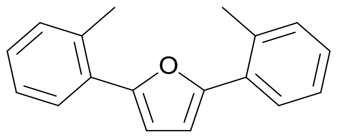

$C_{18}H_{16}O$
248.32

The synthesis was carried out according to the general procedure with 436 mg (2.00 mmol) of *o*-iodotoluene (**3b**) (Alfa Aesar). The crude product was absorbed onto Celite® and purified by column chromatography on silica gel with *n*-hexane as an eluent to give 126 mg (0.51 mmol, 51%) of the desired product as colorless solid.

R_f = 0.31 (*n*-hexane). Mp 70 °C. ^{1}H NMR (DMSO-d_6, 600 MHz): δ = 2.50 (s, 6 H), 6.87 (t, ^3J = 1.9 Hz, 2 H), 7.22 (t, ^3J = 7.4 Hz, 2 H), 7.28 (t, ^3J = 6.9 Hz, 4 H), 7.76 (d, ^3J = 7.2 Hz, 2 H). ^{13}C NMR (DMSO, 150 MHz): δ = 21.7 (CH_3), 111.1 (CH), 125.2 (CH), 126.4 (CH), 127.6 (CH), 129.3 (C_{quat}), 131.3 (CH), 133.9 (C_{quat}), 151.9 (C_{quat}); GC-MS (*m/z* (%)): 248 (M^+, 100), 128 (($C_{10}H_8)^+$, 40), 119 ((M-$C_{10}H_9)^+$, 69), 91 (($C_7H_7)^+$, 71), 77 (($C_6H_5)^+$, 15), 65 (29); IR (KBr): $\tilde{\nu}$ = 1481 (w) cm^{-1}, 1458 (w), 1436 (w), 1030 (w), 928 (w), 789 (w), 756 (s), 721 (m), 671 (w);. Anal. calcd for $C_{18}H_{16}O$ (248.3): C 87.05, H 6.49; Found: C 86.86, H 6.28; UV–vis (CH_2Cl_2): $\lambda_{max}(\varepsilon)$: 314 nm (23000 L·mol^{-1}·cm^{-1}), 342 (9000). Fluorescence (CH_2Cl_2): λ_{max}: 359 nm. *Stokes* shift $\Delta\tilde{\nu}$ = 5200 cm$^{-1.}$ Quantum yield: Φ_f = 59 % (ref.: *p*-terphenyl (Φ_f = 93 % in cyclohexane) [2]). Cyclic voltammetry (CH_2Cl_2): $E_{1/2}^{0/+1}$ = 1.19 V.

2,5-Di-*m*-tolylfuran (2c) [12]

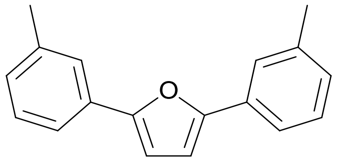

$C_{18}H_{16}O$
248.32

The synthesis was carried out according to the general procedure with 436 mg (2.00 mmol) of *m*-iodotoluene (**3c**) (Sigma-Aldrich). The crude product was absorbed onto Celite® and purified by column chromatography on silica gel with *n*-hexane as an eluent to give 160 mg (0.65 mmol, 65%) of the desired product as colorless solid.

R_f: = 0.24 (*n*-hexane)). Mp 83 °C (colorless oil [12]); ^{1}H NMR (DMSO-d_6, 600 MHz): δ = 2.37 (s, 6 H), 7.03 (s, 2 H), 7.12 (d, ^3J = 7.5 Hz, 2 H), 7.33 (t, ^3J = 7.6 Hz, 2 H), 7.61 (d, ^3J = 7.9 Hz, 2 H), 7.64 (s, 2 H). ^{13}C NMR (DMSO, 150 MHz): δ = 21.0 (CH_3), 108.0 (CH), 120.8 (CH), 123.9 (CH), 128.3 (CH), 128.9 (CH), 130.2 (C_{quat}), 138.2 (C_{quat}), 152.8 (C_{quat}). GC-MS (*m/z* (%)): 248 (M+, 100), 128 (($C_{10}H_8$)$^+$, 20), 119 ((M-$C_{10}H_9$)$^+$, 12), 91 ((C_7H_7)$^+$, 36), 77 ((C_6H_5)$^+$, 11), 65 (21). IR (KBr): $\tilde{\nu}$ = 1476 (w) cm^{-1}, 1260 (w), 1092 (w), 1026 (m), 779 (s), 698 (m). Anal. calcd for $C_{18}H_{16}O$ (248.3): C 87.05, H 6.49; Found: C 86.86, H 6.28. UV–vis (CH_2Cl_2): $\lambda_{max}(\varepsilon)$: 331 nm (33000 L·mol^{-1}·cm^{-1}), 348 (20000). Fluorescence (CH_2Cl_2): λ_{max}: 360 nm. *Stokes* shift $\Delta\tilde{\nu}$ = 3800 cm^{-1}. Quantum yield: Φ_f = 72 % (ref.: *p*-terphenyl (Φ_f = 93 % in cyclohexane) [2]). Cyclic voltammetry (CH_2Cl_2): $E_{1/2}^{0/+1}$ = 1.19 V.

2,5-Di-*p*-tolylfuran (2d) [12]

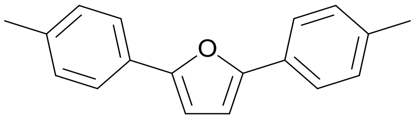

$C_{18}H_{16}O$
248.32

The synthesis was carried out according to the general procedure with 436 mg (2.00 mmol) of *p*-iodotoluene (**3d**) (Sigma-Aldrich). The crude product was absorbed onto Celite® and purified by column chromatography on silica gel with *n*-hexane as an eluent to give 100 mg (0.40 mmol, 40%) of the desired product as colorless solid.

$R_{f.}$: = 0.31 (*n*-hexane). Mp 168 °C (158-160 °C [12]). ^{1}H NMR (DMSO-d_6, 600 MHz): δ = 2.33 (s, 6 H), 6.97 (s, 2 H), 7.25 (d, ^{3}J = 8.4 Hz, 4 H), 7.69 (t, ^{3}J = 8.4, 4 H). ^{13}C NMR (DMSO, 150 MHz): δ = 20.9 (CH), 107.3 (CH), 123.3 (CH), 127.5 (C_{quat}), 129.5 (CH), 136.8 (C_{quat}), 152.4 (C_{quat}). GC-MS (*m/z* (%)): 248 (M+, 100), 205 (6), 129 ($(C_{10}H_7)^+$, 15), 119 ($(M-C_{10}H_9)^+$, 11), 91 ($(C_7H_7)^+$, 24), 77 ($(C_6H_5)^+$, 7), 65 (14); IR (KBr): $\tilde{\nu}$ = 2911 (w) cm^{-1}, 1912 (w), 1501 (m), 1487 (m), 1375 (w), 1287 (w), 1209 (w), 1113 (w), 1063 (w), 1017 (m), 928 (m), 824 (s), 791 (s), (716 (m), 673 (m). Anal. calcd for $C_{18}H_{16}O$ (248.3): C 87.05, H 6.49; Found: C 87.27, H 6.41. UV–vis (CH_2Cl_2): $\lambda_{max}(\varepsilon)$: 331 nm (33000 L·mol^{-1}·cm^{-1}), 348 (20000). Fluorescence (CH_2Cl_2): λ_{max}: 360 nm. *Stokes* shift: $\Delta\tilde{\nu}$ = 3800 cm^{-1}). Quantum yield: Φ_f = 64 % (ref.: *p*-terphenyl (Φ_f = 93 % in cyclohexane) [2]). Cyclic voltammetry (CH_2Cl_2): $E_{1/2}^{0/+1}$ = 1.09 V.

2,5-Bis(3,5-dimethylphenyl)furan (2e)

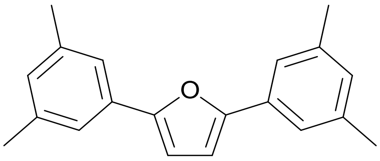

$C_{20}H_{20}O$
276.37

The synthesis was carried out according to the general procedure with 464 mg (2.00 mmol) of 3,5-dimethyliodobenzene (**3e**) (Alfa Aesar). The crude product was absorbed onto Celite® and purified by column chromatography on silica gel with *n*-hexane as an eluent to give 60.0 mg (0.22 mmol, 22%) of the desired product as colorless crystals.

R_f: = 0.43 (*n*-hexane). Mp 147 °C. ^{1}H NMR (DMSO-d_6, 600 MHz): δ = 2.50 (s, 12 H), 6.93 (s, 2 H), 6.99 (s, 2 H), 7.42 (s, 4 H). ^{13}C NMR (DMSO, 150 MHz): δ = 21.0 (CH), 107.9 (CH), 121.2 (CH), 129.1 (CH), 130.0 (C_{quat}), 137.9 (C_{quat}), 152,7 (C_{quat}). GC-MS (*m/z* (%)): 276 (M^+, 100), 233 (4), 143 ($(C_{11}H_{11})^+$, 11), 128 (11), 115 (10), 105 ($(C_8H_9)^+$, 11), 77 (14). IR (KBr): $\tilde{\nu}$ = 2913 (m) cm^{-1}, 1601 (m), 1466 (m), 1377 (w), 1312 (w), 1207 (w), 1028 (m), 941 (m), 891 (m), 845 (s), 791 (s), 698 (m), 675 (m). Anal. calcd for $C_{20}H_{20}O$ (276.4): C 86.92, H 7.29; Found: C 86.73, H 7.19. UV–vis (CH_2Cl_2): $\lambda_{max}(\varepsilon)$: 331 nm (28000 L·mol^{-1}·cm^{-1}), 347 (18000). Fluorescence (CH_2Cl_2): λ_{max}: 362 nm. *Stokes* shift: $\Delta\tilde{\nu}$ = 3800 cm^{-1}. Quantum yield: Φ_f = 55 % (ref.: *p*-terphenyl (Φ_f = 93 % in cyclohexane) [2]). Cyclic voltammetry (CH_2Cl_2): $E_{1/2}^{0/+1}$ = 1.06 V.

2,5-Bis(3-methoxyphenyl)furan (2f) [12]

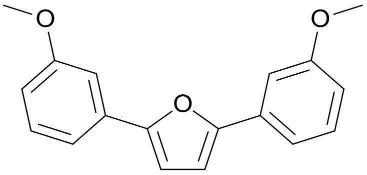

$C_{18}H_{16}O_3$
280.32

The synthesis was carried out according to the general procedure with 468 mg (2.00 mmol) of m-methoxyiodobenzene (**3f**) (Sigma Aldrich). The crude product was absorbed onto Celite® and purified by column chromatography on silica gel with *n*-hexane as an eluent to give 110 mg (0.39 mmol, 39%) of the desired product as beige solid.

R_f: = 0.08 (*n*-hexane). Mp 85 °C (colorless oil [12]). ^{1}H NMR (DMSO-d_6, 600 MHz): δ = 3.83 (s, 6 H), 6.89 (ddd, ^3J = 8.1 Hz, ^4J = 2.4 Hz, ^5J = 1.2 Hz, 2 H), 7.10 (s, 2 H), 7.34 – 7.37 (m, 4 H), 7.40 (td, ^3J = 7.8 Hz, ^4J = 1.2 Hz, 4 H). ^{13}C NMR (DMSO, 150 MHz): δ= 55.2 (CH), 108.6 (CH), 108.8 (CH), 113.3 (CH), 115.9 (CH), 130.1 (CH), 131.1 (C_{quat}), 152.5 (C_{quat}), 159.7 (C_{quat}). GC-MS (*m/z* (%)): 280 (M^+, 100), 237 (23), 194 (16), 165 (13), 145 (9), 102 (21), 92 (17), 77 (22), 63 (16), 51 (9). IR (KBr): $\tilde{\nu}$ = 2959 (w) cm^{-1}, 1611 (w), 1584 (m), 1485 (s), 1431 (m), 1279 (m), 1211 (s), 1169 (m), 1099 (w), 1038 (s), 864 (m), 796 (s), 777 (m), 692 (m), 681 (m). Anal. calcd for $C_{18}H_{16}O_3$ (280.3): C 77.12, H 5.75; Found: C 77.04, H 5.89. UV–vis (CH_2Cl_2): $\lambda_{max}(\varepsilon)$: 331 nm (29000 $L\cdot mol^{-1}\cdot cm^{-1}$), 347 (20000). Fluorescence (CH_2Cl_2): λ_{max}: 362 nm. *Stokes* shift: $\Delta\tilde{\nu}$ = 4000 cm^{-1}); Quantum yield: Φ_f = 95 % (ref.: *p*-terphenyl (Φ_f = 93 % in cyclohexane) [2]).

2,5-Bis(3,4,5-trimethoxyphenyl)furan (2g) [13]

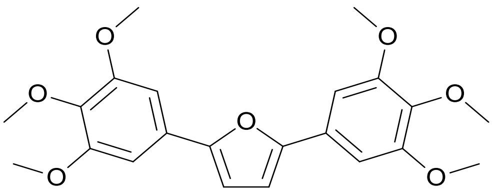

$C_{22}H_{24}O_7$
400.42

The synthesis was carried out according to the general procedure with 588 mg (2.00 mmol) of 3,4,5-trimethoxyiodobenzene (**3g**). The crude product was absorbed onto Celite® and purified by column chromatography on silica gel with 5/1 *n*-hexane/THF as an eluent to give 108 mg (0.27 mmol, 27%) of the desired product as colorless solid.

R_f: = 0.18 (5/1 *n*-hexane/THF). Mp 139 °C (140-140.9 °C [13]). ^{1}H NMR (DMSO-d_6, 600 MHz): δ = 3.70 (s, 6 H), 3.88 (s 12 H), 7.07 (s 2 H), 7.09 (s 4 H). ^{13}C NMR (DMSO, 150 MHz): δ = 56.0 (CH_t), 60.1 (CH), 101.2 (CH), 108.1 (CH), 125.8 (C_{quat}), 137.8 (C_{quat}), 152.4 (C_{quat}), 153.4 (C_{quat}). MS-EI (*m/z* (%)): 400 (M^+, 4), 293 (13), 195 ($(C_9H_{11}O_3)^+$, 3), 167 (19), 148 (100), 71 (23). IR (KBr): $\tilde{\nu}$ = 2951 (w) cm^{-1}, 1586 (m), 1493 (m) 1464 (m), 1414 (m), 1341 (m), 1240 (m), 1184 (w), 1123 (s), 1088 (m), 1022 (m), 1007 (m), 997 (m), 968 (m), 831 (m), 789 (m), 762 (m), 691 (m). Anal. calcd for $C_{22}H_{24}O_7$ (400.4): C 66.59, H 6.04; Found: C 66.32, H 5.90. UV–vis (CH_2Cl_2): $\lambda_{max}(\varepsilon)$: 340 nm (33000 L·mol^{-1}·cm^{-1}), 365 (20000). Fluorescence (CH_2Cl_2): λ_{max}: 378 nm. *Stokes* shift: $\Delta\tilde{\nu}$ = 3800 cm^{-1}. Quantum yield: Φ_f = 80 % (ref.: *p*-terphenyl (Φ_f = 93 % in cyclohexane) [2]).

2,5-Bis(2-methoxy-5-methylphenyl)furan (2h) [14]

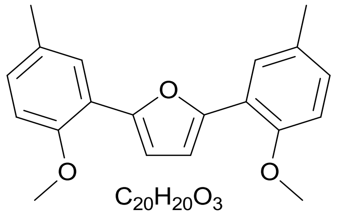

$C_{20}H_{20}O_3$
308.37

The synthesis was carried out according to the general procedure with 496 mg (2.00 mmol) of 2-iodo-1-methoxy-4-methylbenzene (**3h**). The crude product was absorbed onto Celite® and purified by column chromatography on silica gel with *n*-hexane as an eluent to give 128 mg (0.42 mmol, 42%) of the desired product as yellow solid.

R_f: = 0.32 (*n*-hexane). Mp 118 °C (110 °C [14]). ^{1}H NMR (DMSO-d_6, 600 MHz): δ = 2.34 (s, 6 H), 3.89 (s 6 H), 7.00 (t, ^3J = 4.2 Hz, 4 H), 7.09 (dd, ^3J = 6.6 Hz, ^4J = 1.8 Hz, 2 H), 7.75 (ds, ^4J = 1.8 Hz, 2 H). ^{13}C NMR (DMSO, 150 MHz): δ = 20.3 (CH), 55.9 (CH), 111.6 (CH), 112.3 (CH), 118.4 (C_{quat}), 125.5 (CH), 128.8 (CH), 129.4 (C_{quat}), 148.1 (C_{quat}), 153.2 (C_{quat}). GC-MS (*m/z* (%)): 308 (M^+, 100), 265 (42), 149 (($C_9H_9O_2)^+$, 23), 145 (45), 115 (40), 91 (32), 78 (45). IR (KBr): $\tilde{\nu}$ = 2926 (w) cm^{-1}, 1609 (w), 1503 (s), 1462 (m), 1439 (m), 1273 (m), 1244 (s), 1179 (m), 1146 (m), 1028 (s), 885 (w), 795 (s), 739 (m). Anal. calcd for $C_{20}H_{20}O_3$ (308.4): C 77.90, H 6.54; Found: C 77.93, H 6.59. UV–vis (CH_2Cl_2): $\lambda_{max}(\varepsilon)$: 347 nm (33000 L·mol^{-1}·cm^{-1}), 364 (26000). Fluorescence (CH_2Cl_2): λ_{max}: 377 nm. *Stokes* shift: $\Delta\tilde{\nu}$ = 3500 cm^{-1}. Quantum yield: Φ_f = 47 % (ref.: *p*-terphenyl (Φ_f = 93 % in cyclohexane) [2]). Cyclic voltammetry (CH_2Cl_2): $E_{1/2}^{0/+1}$ = 0.95 V.

2,5-Bis(naphthalen-1-yl)furan (2i) [15]

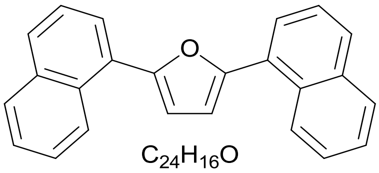

$C_{24}H_{16}O$
320.38

The synthesis was carried out according to the general procedure with 508 mg (2.00 mmol) of 1-iodonaphtalene (**3i**). The crude product was absorbed onto Celite® and purified by column chromatography on silica gel with *n*-hexane as an eluent to give 191 mg (0.60 mmol, 60%) of the desired product as beige solid.

R_f: = 0.43 (*n*-hexane). Mp 76 °C (yellow oil [15]). ^{1}H NMR (DMSO-d_6, 600 MHz): δ = 7.19 (s, 2 H), 7.58 – 7.65 (m, 6 H), 7.94 (d, ^3J = 7.1 Hz, 2 H), 7.99 (d, ^3J = 8.2 Hz, 2 H), 8.00 (d, ^3J = 8.0 Hz, 2 H), 8.53 (d, ^3J = 8.4 Hz, 2 H). ^{13}C NMR (DMSO, 150 MHz): δ = 111.8 (CH), 124.9 (CH), 125.6 (CH), 126.0 (CH), 126.2 (CH), 127.1 (CH), 127.5 (C_{quat}), 128.7 (CH), 129.3 (C_{quat}), 133.7 (C_{quat}), 152.6 (C_{quat}). GC-MS (*m/z* (%)): 320 (M^+, 100), 289 (9), 165 ($(C_{13}H_9)^+$, 30), 127 ($(C_{10}H_7)^+$, 57), 77 (12).IR (KBr): $\tilde{\nu}$ = 1479 (w) cm^{-1}, 1446 (w), 1155 (w), 1022 (m), 925 (w), 910 (w), 794 (m), 756 (s), 689 (s), 671 (m). Anal. calcd for $C_{24}H_{16}O$ (320.4): C 89.97, H 5.03; Found: C 89.69, H 5.08. UV–vis (CH_2Cl_2): $\lambda_{max}(\varepsilon)$: 347 nm (29000 L·mol^{-1}·cm^{-1}). Fluorescence (CH_2Cl_2): λ_{max}: 424 nm. *Stokes* shift: $\Delta\tilde{\nu}$ = 5300 cm^{-1}. Quantum yield: Φ_f = 75 % (ref.: coumarin 1 (Φ_f = 73 % in EtOH) [3]). Cyclic voltammetry (CH_2Cl_2): $E_{1/2}^{0/+1}$ = 1.15 V.

2,5-Bis(naphthalen-2-yl)furan (2j) [15]

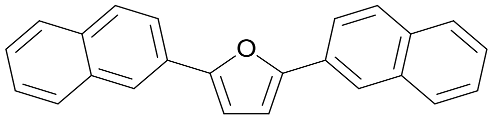

$C_{24}H_{16}O$
320.38

The synthesis was carried out according to the general procedure with 508 mg (2.00 mmol) of 2-iodonapthalene (**3j**). The crude product was absorbed onto Celite® and purified by column chromatography on silica gel with *n*-hexane as an eluent to give 96.0 mg (0.21 mmol, 21%) of the desired product as brown solid.

R_f: = 0.28 (*n*-hexane). Mp 201 °C (219-220 °C [15]). ^{1}H NMR ($CDCl_3$, 600 MHz): δ = 6.84 (s, 2 H), 7.40 (t, ^3J = 7.2 Hz, 2 H), 7.44 (t, ^3J = 7.2, 2 H), 7.76 (d, ^3J = 7.9, 2 H), 7.81 (s, 4 H), 7.86 (d, ^3J = 8.0, 2 H). ^{13}C NMR (DMSO, 150 MHz): δ = 107.1 (CH), 121.1 (CH), 121.4 (CH), 124.9 (CH), 125.5 (CH), 126.8 (CH), 127.0 (C_{quat}), 127.2 (CH), 127.4 (CH), 131.7 (C_{quat}), 132.7 (C_{quat}), 152.7 (C_{quat}). GC-MS (*m/z* (%)): 320 (M^+, 100), 289 (6), 165 ($(C_{13}H_9)^+$, 16), 127 ($(C_{10}H_7)^+$, 18), 77 (3). IR (KBr): $\tilde{\nu}$ = 2922 (w) cm^{-1}, 1661 (w), 1624 (w), 1599 (w), 1501 (w), 1375 (w), 1260 (m), 1090 (m), 1024 (m), 1015 (m), 795 (s), 741 (s). HRMS calcd for $C_{24}H_{16}O^+$: 320.1196; Found: 320.1197. UV–vis (CH_2Cl_2): $\lambda_{max}(\varepsilon)$: 358 nm (26000 $L\cdot mol^{-1}\cdot cm^{-1}$), 377 (21000). Fluorescence (CH_2Cl_2): λ_{max}: 393 nm. *Stokes* shift: $\Delta\tilde{\nu}$ = 3600 cm^{-1}. Quantum yield: Φ_f = 100 % (ref.: coumarin 1 (Φ_f = 73 % in EtOH) [3]). Cyclic voltammetry (CH_2Cl_2): $E_{1/2}^{0/+1}$ = 1.20 V.

2,5-Bis(3-hydroxymethylphenyl)furan (2k)

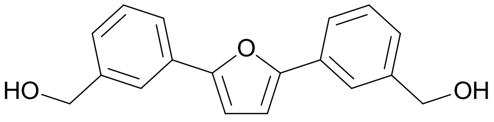

$C_{18}H_{16}O_3$
280.32

The synthesis was carried out according to the general procedure with 636 mg (2.00 mmol) of THP protected 3-iodobenzyl alcohol (**3k**). The crude product was absorbed onto Celite® and purified by column chromatography on silica gel with *n*-hexane as an eluent to give 120 mg (0.43 mmol, 43%) of the desired product as beige solid.

R_f: = 0.29 (*n*-hexane). Mp 146 °C. ^{1}H NMR (DMSO-d_6, 600 MHz): δ = 4.57 (d, ^3J = 6.0 Hz, 4 H), 5.30 (t, ^3J = 5.4 Hz, 2 H), 7.06 (s, 2 H), 7.27 (d, ^3J = 7.2 Hz, 2 H), 7.41 (t, ^3J = 6.0 Hz, 2 H), 7.68 (d, ^3J = 7.8 Hz, 2 H), 7.77 (s, 2 H). ^{13}C NMR (DMSO, 150 MHz): δ = 62.8 (CH_2), 108.1 (CH), 121.3 (CH), 121.9 (CH), 125.7 (CH), 128.7 (CH), 129.9 (C_{quat}), 143.4 (C_{quat}), 152.7 (C_{quat}). MS-EI (*m/z* (%)): 280 (M^+, 100), 261 (5), 202 (6), 191 (5), 145 (($C_{10}H_9O)^{+,}$ 11), 135 (($C_8H_7O_3)^{+,}$ 7), 107 (($C_7H_7O)^+$, 4),89 (8), 77 (6). IR (KBr): $\tilde{\nu}$ = 3277 (m) cm^{-1}, 2924 (w), 1601 (w), 1476 (w), 1452 (m), 1414 (m), 1294 (m), 1184 (m), 1045 (m), 1026 (s), 1003 (s), 941 (m), 897 (m), 901 (s), 775 (s), 694 (s), 613 (m). Anal. calcd for $C_{18}H_{16}O_3$ (280.3): C 77.12, H 5.75; Found: C 76.88, H 5.82. UV–vis (CH_2Cl_2): $\lambda_{max}(\varepsilon)$: 329 nm (32000 L·mol^{-1}·cm^{-1}), 345 (22000). Fluorescence (CH_2Cl_2): λ_{max}: 362 nm. *Stokes* shift: $\Delta\tilde{\nu}$ = 3900 cm^{-1}. Quantum yield: Φ_f = 80 % (ref.: *p*-terphenyl (Φ_f = 93 % in cyclohexane) [3]). Cyclic voltammetry (CH_2Cl_2): $E_{1/2}^{0/+1}$ = 1.16 V.

2,5-Bis(thiophen-2-yl)furan (2l) [16]

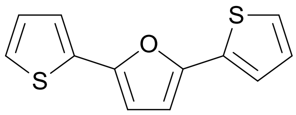

$C_{12}H_8OS_2$
232.32

The synthesis was carried out according to the general procedure with 420 mg (2.00 mmol) of 2-iodothiophene (**3l**). The crude product was absorbed onto Celite® and purified by column chromatography on silica gel with *n*-hexane as an eluent to give 73.0 mg (0.31 mmol, 31 %) of the desired product as colorless solid.

R_f: 0.31 (*n*-hexane). Mp 78 °C (81-82 °C [16]). ^{1}H NMR (DMSO-d_6, 600 MHz): δ = 6.85 (s, 2 H), 7.13 (t, ^3J = 3.7 Hz, 2 H), 7.42 (d, ^3J = 3.6 Hz, 2 H), 7.55 (d, ^3J = 5.0 Hz, 2 H). ^{13}C NMR (DMSO, 150 MHz): δ = 107.8 (CH), 123.2 (CH), 125.4 (CH), 128.2 (CH), 132.3 (C_{quat}), 147.9 (C_{quat}). GC-MS (*m/z* (%)): 232 (M^+, 100), 203 (29), 171 (31), 121 $(M-C_5H_3OS)^+$, 26), 111 ($(M-C_7H_5O)^+$, 34), 83 ($(C_4H_3S)^+$, 12), 77 (17), 69 (17), 63 (13), 58 (16), 51 (11). IR (KBr): $\tilde{\nu}$ = 3105 (w) cm^{-1}, 1796 (w), 1429 (w), 1260 (w), 1003 (s), 843 (m), 826 (m), 789 (s), 691 (s). Anal. calcd for $C_{12}H_8OS_2$ (232.3): C 62.04, H 3.47, S 27.60; Found: C 62.27, H 3.39, S 27.73. UV–vis (CH_2Cl_2): $\lambda_{max}(\varepsilon)$: 353 nm (24000 L·mol^{-1}·cm^{-1}), 371 (15000). Fluorescence (CH_2Cl_2): λ_{max}: 389 nm. *Stokes* shift: $\Delta\tilde{\nu}$ = 3800 cm^{-1}. Quantum yield: Φ_f = 42 % (ref.: *p*-terphenyl (Φ_f = 93 % in cyclohexane) [2]).

2,5-Bis(thiophen-3-yl)furan (2m) [17]

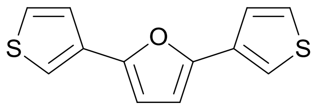

$C_{12}H_8OS_2$
232.32

The synthesis was carried out according to the general procedure with 420 mg (2.00 mmol) of 3-iodothiophene (**3m**). The crude product was absorbed onto Celite® and purified by column chromatography on silica gel with *n*-hexane as an eluent to give 45.0 mg (0.20 mmol, 20 %) of the desired product as colorless solid.

R_f: = 0.22 (*n*-hexane). Mp 131 °C (130-131 °C [17]). ^{1}H NMR (DMSO-d_6, 600 MHz): δ = 6.82 (s, 2 H), 7.50 (dd, ^5J = 0.6 Hz, ^3J = 5.0 Hz, 2 H), 7.64-7.65 (m, 2 H), 7.79 (dd, ^5J = 1.1 Hz, ^4J = 3.0 Hz, 2 H). ^{13}C NMR (DMSO, 150 MHz): δ = 107.3 (C_{quat}), 119.2 (C_{quat}), 124.7 (C_{quat}), 127.4 (C_{quat}), 131.9 (CH), 149.3 (CH). GC-MS (*m/z* (%)): 232 (M^+, 100), 203 (20.6), 171 (13.9), 121 ((M-$C_7H_8S^+$) 20.9), 111 ((M-$C_5H_5OS)^+$, 25.0), 83 ((M-$C_5H_3S)^+$, 11.4). IR (KBr): $\tilde{\nu}$ = 3101 (w), 2920 (w), 1573 (w), 1483 (w) 1413 (w), 1269 (w), 1085 (w), 1018 (m), 848 (m), 769 (s), 684 (m). HRMS calcd for $C_{13}H_8OS_2^+$: 233.0089; Found: 232.0090 UV–vis (CH_2Cl_2): $\lambda_{max}(\varepsilon)$: 321 nm (23000 L·mol^{-1}·cm^{-1}), 336 (15000). Fluorescence (CH_2Cl_2): λ_{max}: 351 nm. *Stokes* shift: $\Delta\tilde{\nu}$ = 3900 cm^{-1}. Quantum yield: Φ_f = 29 % (ref.: *p*-terphenyl (Φ_f = 93 % in cyclohexane) [2]).

2,5-Bis(phenanthren-9-yl)furan (2n)

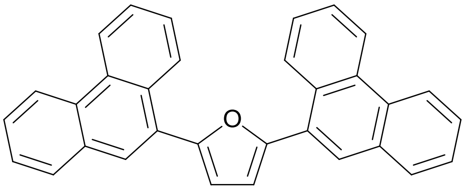

$C_{32}H_{20}O$
420.50

The synthesis was carried out according to the general procedure with 608 mg (2.00 mmol) of 9-iodophenanthrene (**3n**). The crude product was absorbed onto Celite® and purified by column chromatography on silica gel with 80/1 *n*-Hexane/THF as an eluent to give 215 mg (0.51 mmol, 51%) of the desired product as yellow solid.

R_f: = 0.30 (40/1 *n*-hexane/THF). Mp 157 °C. ^{1}H NMR (DMSO-d_6 measured at 80 °C (DMSO-d_6 Signal locked at 3.5 ppm), 600 MHz): δ = 7.21 (s, 2.H), 7.68 (dt, ^3J = 7.2 Hz, ^4J = 1.2 Hz, 2 H), 7.72 - 7.79 (m, 6 H), 8.09 (d, 2 H), 8.27 (s, 2 H), 8.57 (dd, ^{3}J = 9, ^4J = 1.8 Hz, 2 H), 8.83 (d, ^3J = 7.8 Hz, 2 H), 8.92 (dd, ^3J = 9 Hz, ^4J = 1.8 Hz, 2 H). ^{13}C NMR (DMSO-d_6 measured at 80 °C (DMSO-*d*6 Signal locked at 39.5 ppm), 150 MHz): δ = 112.4 (CH), 123.2 (CH), 123.9 (CH), 126.3 (C_{quat}), 127.0 (CH), 127.5 (CH), 127.6 (CH), 127.7 (CH), 127.8 (CH), 128.0 (CH), 129.3 (C_{quat}), 129.5 (CH), 130.3 (C_{quat}), 131.0 (C_{quat}), 131.4 (C_{quat}), 153.2 (C_{quat}). MS-EI (*m/z* (%)): 420 (M^+, 100), 389 (12), 376 (11), 215 ($(C_{17}H_{11})^+$, 22), 205 ($(C_{15}H_9O)^+$, 14), 188 (22), 177 ($(C_{14}H_9)^+$, 21); IR (KBr): $\tilde{\nu}$ = 3053 (w) cm^{-1}, 1940 (w), 1609 (w), 1493 (w), 1447 (m), 1425 (m), 1250 (m), 1140 (m), 897 (s), 764 (s), 743 (s), 717 (s). Anal. calcd for $C_{32}H_{20}O$ (420.5): C 91.40, H 4.79; Found: C 91.18, H 5.08. UV–vis (CH_2Cl_2): $\lambda_{max}(\varepsilon)$: 343 nm (23000 L·mol^{-1}·cm^{-1}). Fluorescence (CH_2Cl_2): λ_{max}: 439 nm. *Stokes* shift: $\Delta\tilde{\nu}$ = 6400 cm^{-1}. Quantum yield: Φ_f = 69 % (ref.: coumarin 1 (Φ_f = 73 % in EtOH) [3]). Cyclic voltammetry (CH_2Cl_2): $E_{1/2}^{0/+1}$ = 1.24 V.

2,5-Bis(4-fluorophenyl)furan (2o) [12]

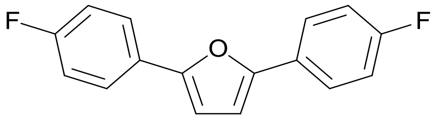

$C_{16}H_{10}F_2O$
256.25

The synthesis was carried out according to the general procedure with 444 mg (2.00 mmol) of p-fluoroiodobenzene (**3o**) (ABCR). The crude product was absorbed onto Celite® and purified by column chromatography on silica gel with *n*-hexane as an eluent to give 63.0 mg (0.25 mmol, 25%) of the desired product as colorless crystals.

R_f: = 0.33 (*n*-hexane). Mp 146 °C (140-141 °C [12]). ^{1}H NMR (DMSO-d_6, 600 MHz): δ = 7.05 (s, 2 H), 7.29 (t, ^3J = 6.0 Hz, 2 H), 7.86 (dt, ^3J = 5.4 Hz, ^4J = 3 Hz, 4 H). ^{13}C NMR (DMSO, 150 MHz): δ = 108.0 (CH), 115.9 (d, ^2J = 21.9 Hz, CH), 125.5 (d, ^3J = 8.1 Hz, CH), 126.7 (d, ^4J = 3 Hz, C_{quat}), 151.7 (C_{quat}), 161.5 (d, ^1J = 243.5 Hz, C_{quat}). GC-MS (*m/z* (%)): 256 (M^+, 100), 227 (13), 207 (5), 133 ($(C_9H_6F)^+$, 22), 123 ($(C_7H_4FO)^+$, 18), 95 ($(C_6H_4F)^+$, 16). IR (KBr): $\tilde{\nu}$ = 2924 (w) cm^{-1}, 1599 (m), 1487 (s), 1300 (m), 1229 (s), 1157 (m), 1103 (m), 1026 (m), 928 (m), 829 (s), 810 (m), 787 (m), 774 (s), 664 (m), 635 (m), 623 (s). Anal. calcd for $C_{16}H_{10}F_2O$ (256.3): C 74.99, H 3.93; Found.: C 74.82, H 4.16. UV–vis (CH_2Cl_2): $\lambda_{max}(\varepsilon)$: 323 nm (21000 L·mol^{-1}·cm^{-1}), 338 (12000). Fluorescence (CH_2Cl_2): λ_{max}: 353 nm. Stokes shift: $\Delta\tilde{\nu}$ = *3800* cm^{-1}. Quantum yield: Φ_f = 76 % (ref.: *p*-terphenyl (Φ_f = 93 % in cyclohexane) [2]). Cyclic voltammetry (CH_2Cl_2): $E_{1/2}^{0/+1}$ = 1.24 V, reversible.

5. UV-absorption and fluorescence data of compounds 2a–2o

$E_{0\text{-}0}$ was determined with the cross-section of the absorption and emission spectra ($E_{0-0} = \lambda_{cross}$).

2,5-Diphenylfuran (2a)

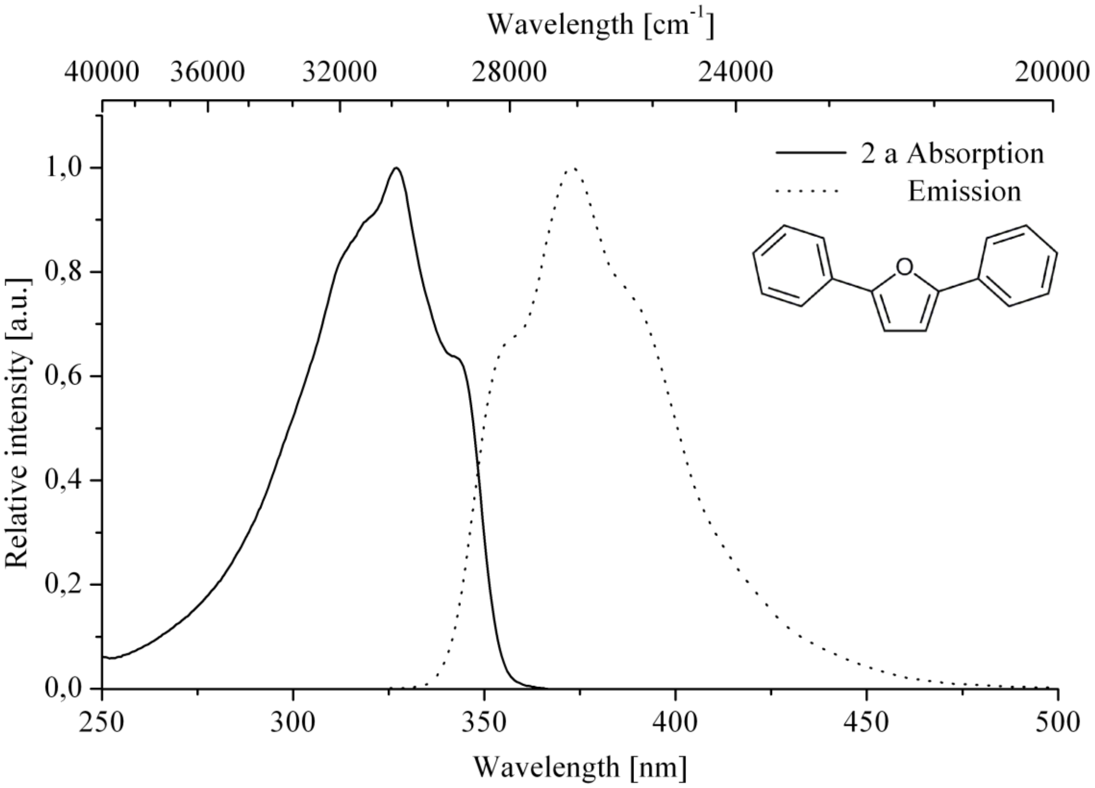

Normalized UV–vis and fluorescence spectra (recorded in dichloromethane at T = 20 °C).

2,5-Di-*o*-tolylfuran (2b)

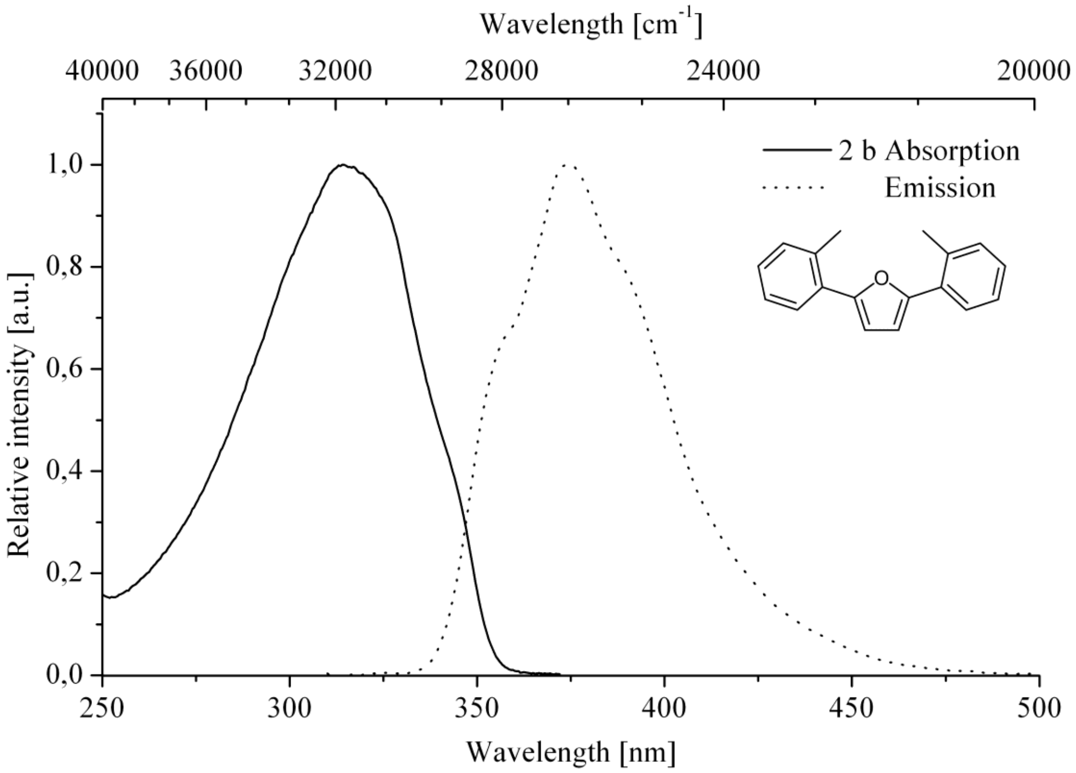

Normalized UV–vis and fluorescence spectra (recorded in dichloromethane at T = 20 °C).

2,5-Di-*m*-tolylfuran (2c)

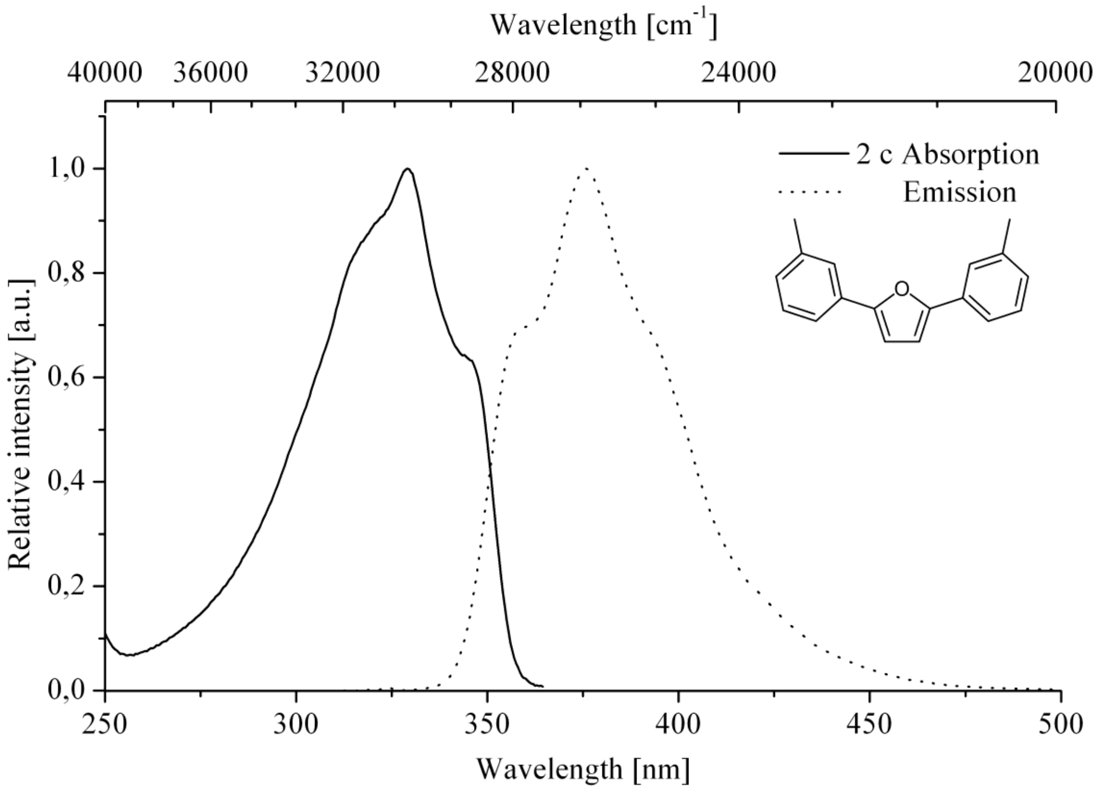

Normalized UV–vis and fluorescence spectra (recorded in dichloromethane at T = 20 °C).

2,5-Di-*p*-tolylfuran (2d)

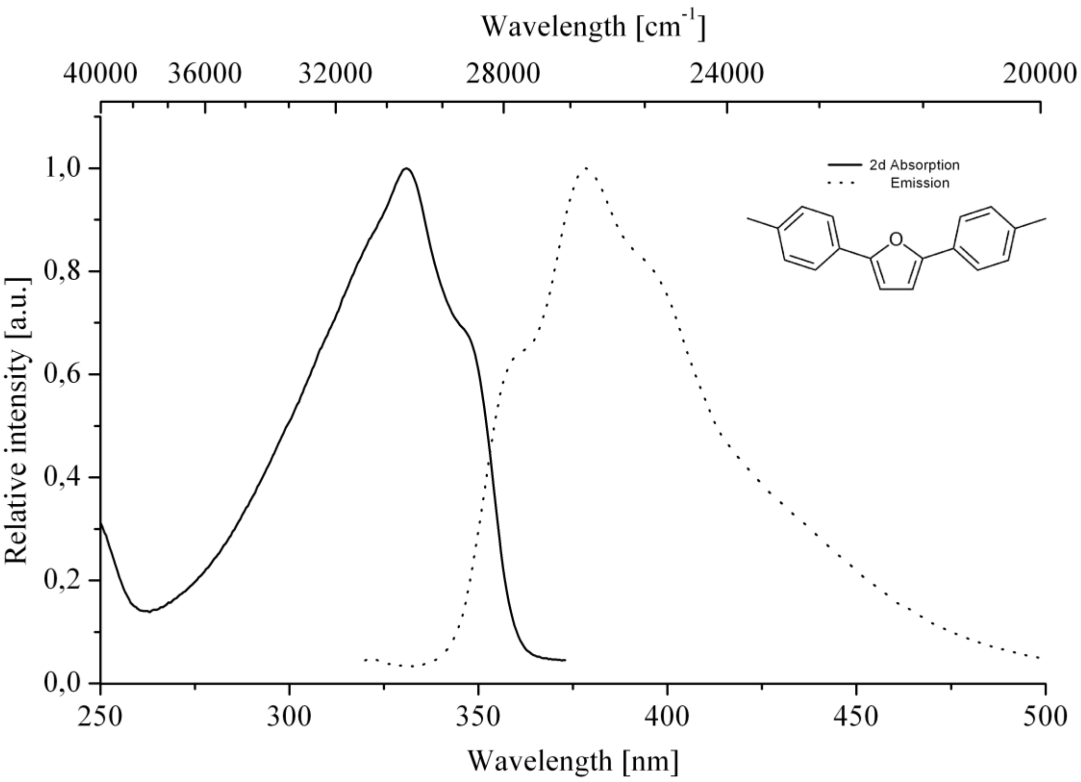

Normalized UV–vis and fluorescence spectra (recorded in dichloromethane at T = 20 °C).

2,5-Bis(3,5-dimethylphenyl)furan (2e)

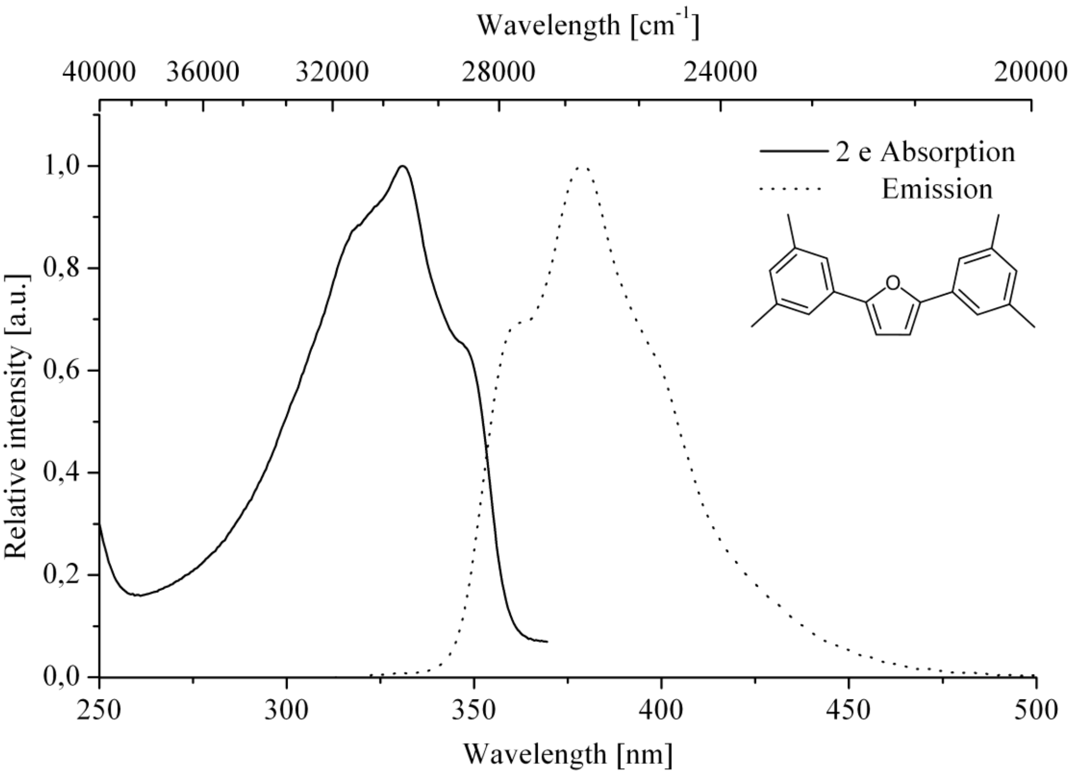

Normalized UV–vis and fluorescence spectra (recorded in dichloromethane at T = 20 °C).

2,5-Bis(3-methoxyphenyl)furan (2f)

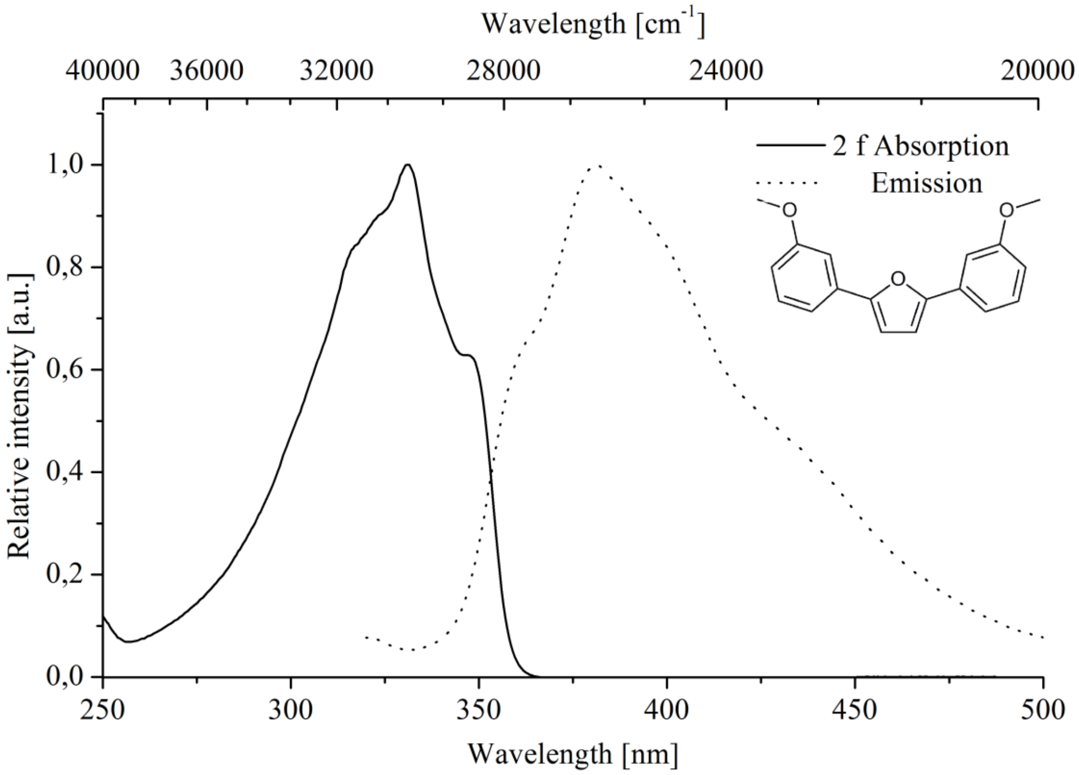

Normalized UV–vis and fluorescence spectra (recorded in dichloromethane at *T* = 20 °C).

2,5-Bis(3,4,5-trimethoxyphenyl)furan (2g)

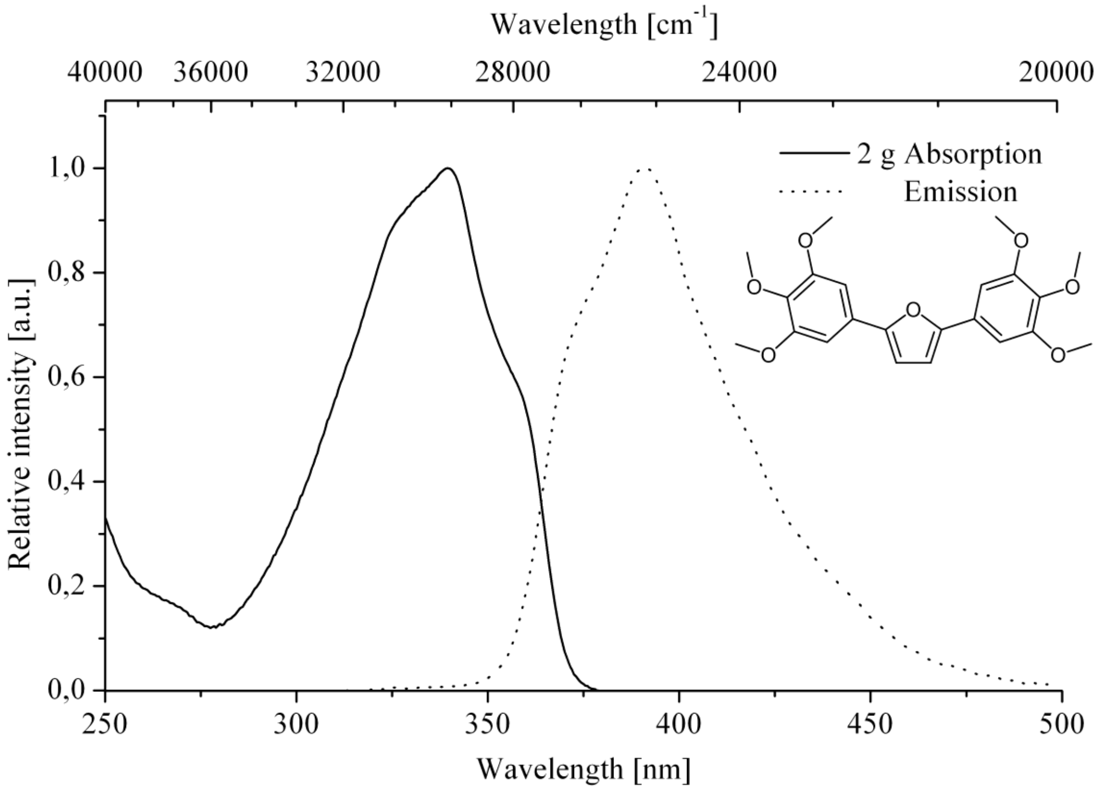

Normalized UV–vis and fluorescence spectra (recorded in dichloromethane at *T* = 20 °C).

2,5-Bis(2-methoxy-5-methylphenyl)furan (2h)

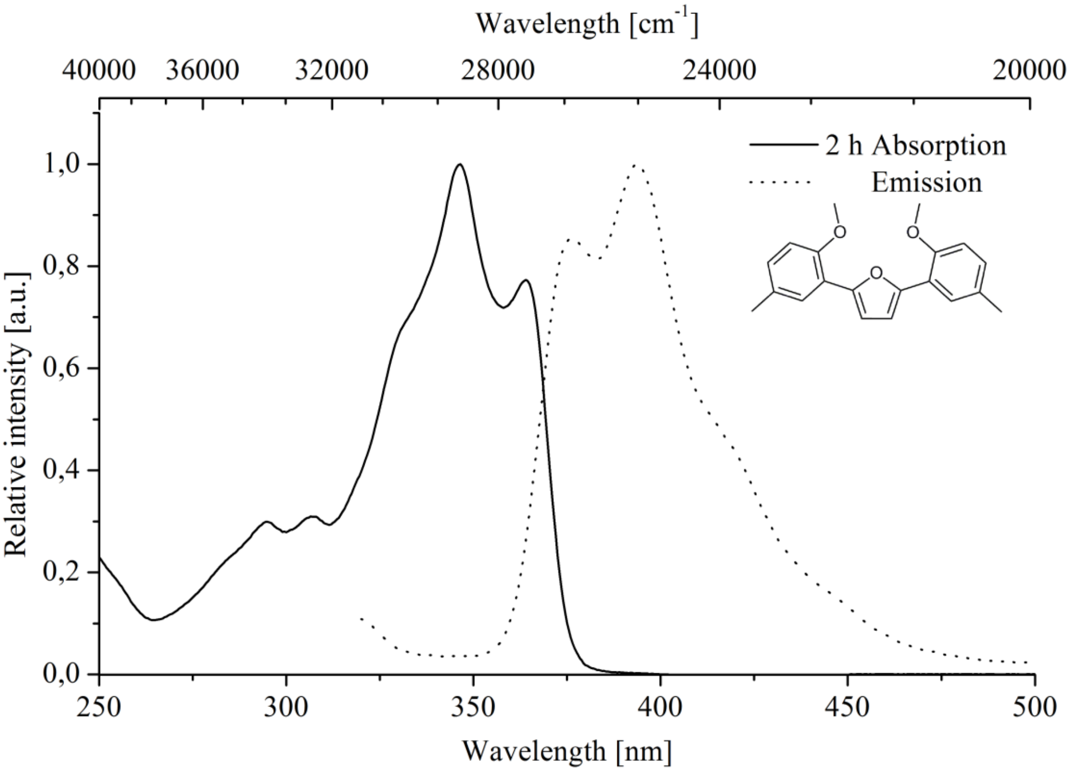

Normalized UV–vis and fluorescence spectra (recorded in dichloromethane at *T* = 20 °C).

2,5-Di(naphthalen-1-yl)furan (2i)

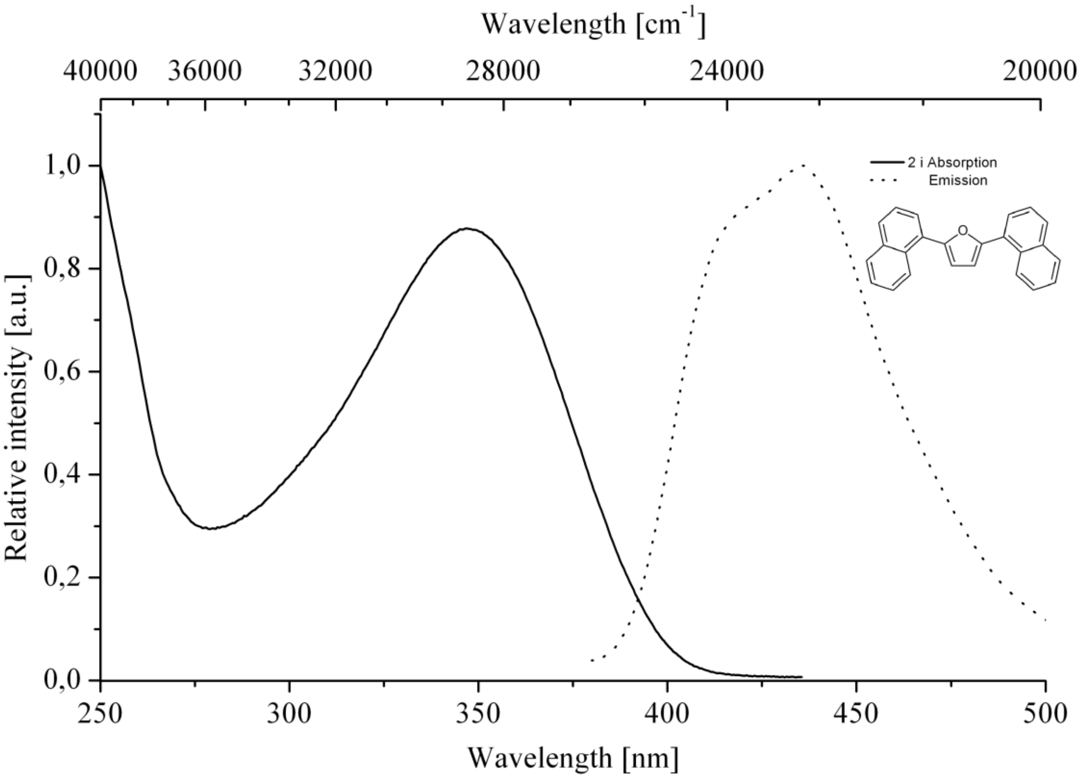

Normalized UV–vis and fluorescence spectra (recorded in dichloromethane at *T* = 20 °C).

2,5-Di(naphthalen-2-yl)furan (2j)

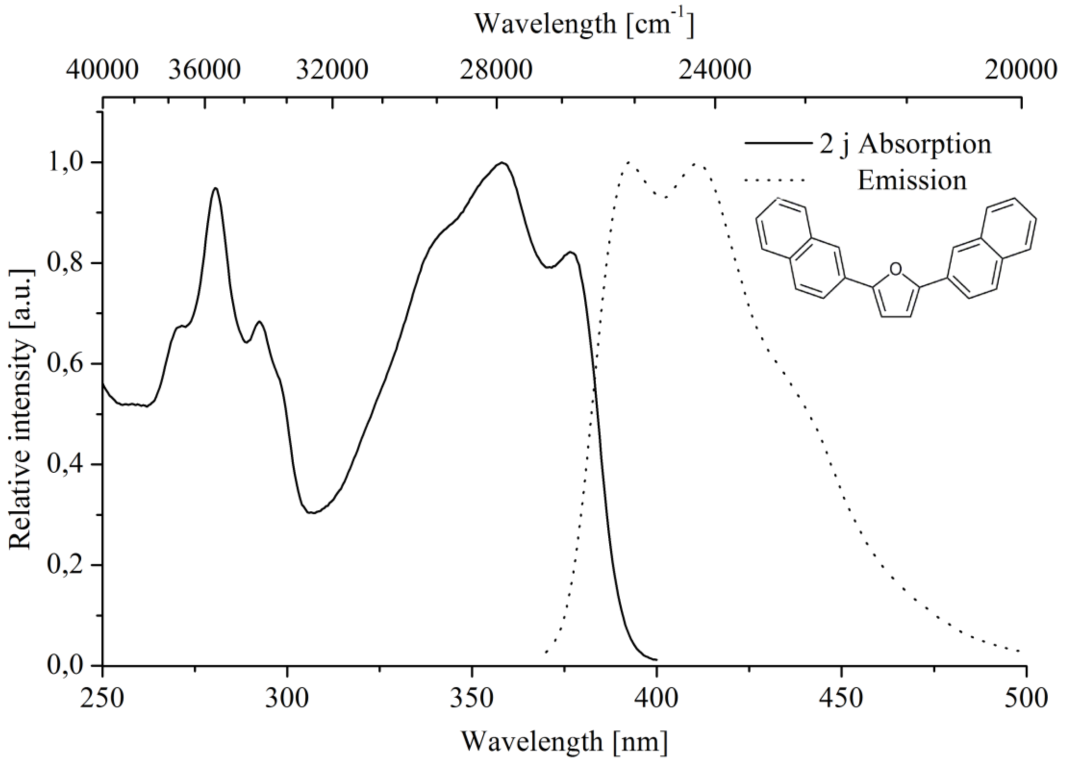

Normalized UV–vis and fluorescence spectra (recorded in dichloromethane at *T* = 20 °C).

2,5-Bis(3-hydroxymethylphenyl)furan (2k)

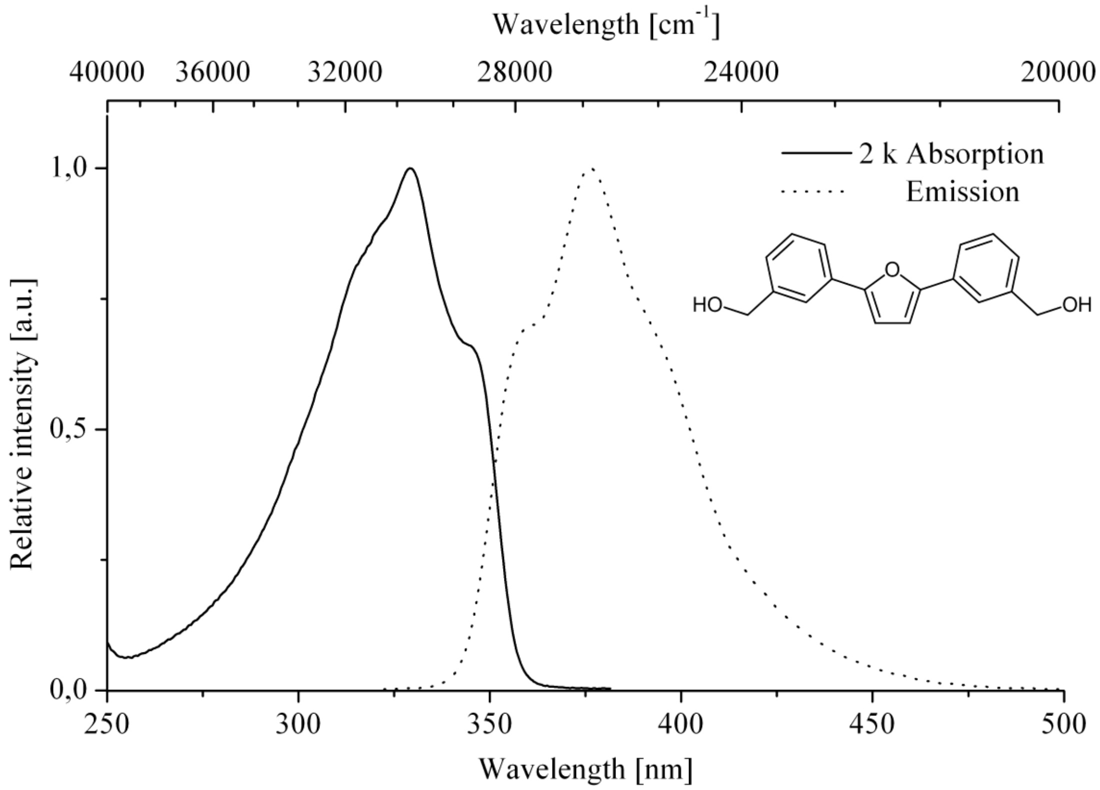

Normalized UV–vis and fluorescence spectra (recorded in dichloromethane at *T* = 20 °C).

2,5-Di(thiophen-2-yl)furan (2l)

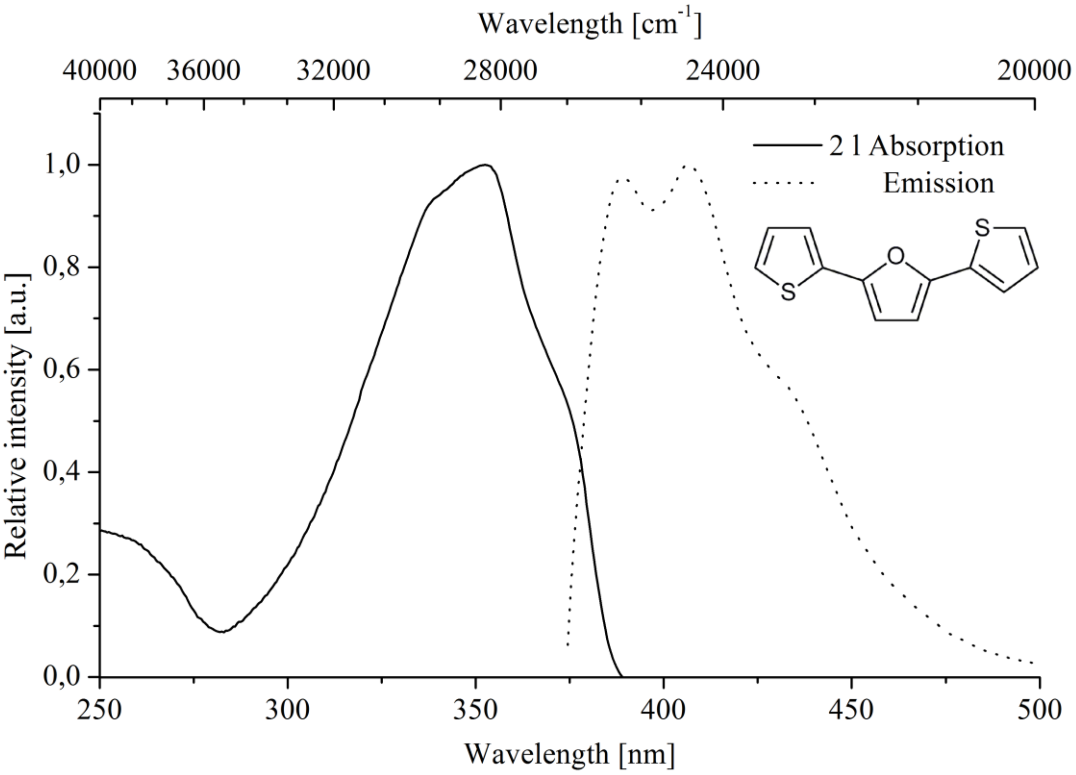

Normalized UV–vis and fluorescence spectra (recorded in dichloromethane at *T* = 20 °C).

2,5-Di(thiophen-3-yl)furan (2m)

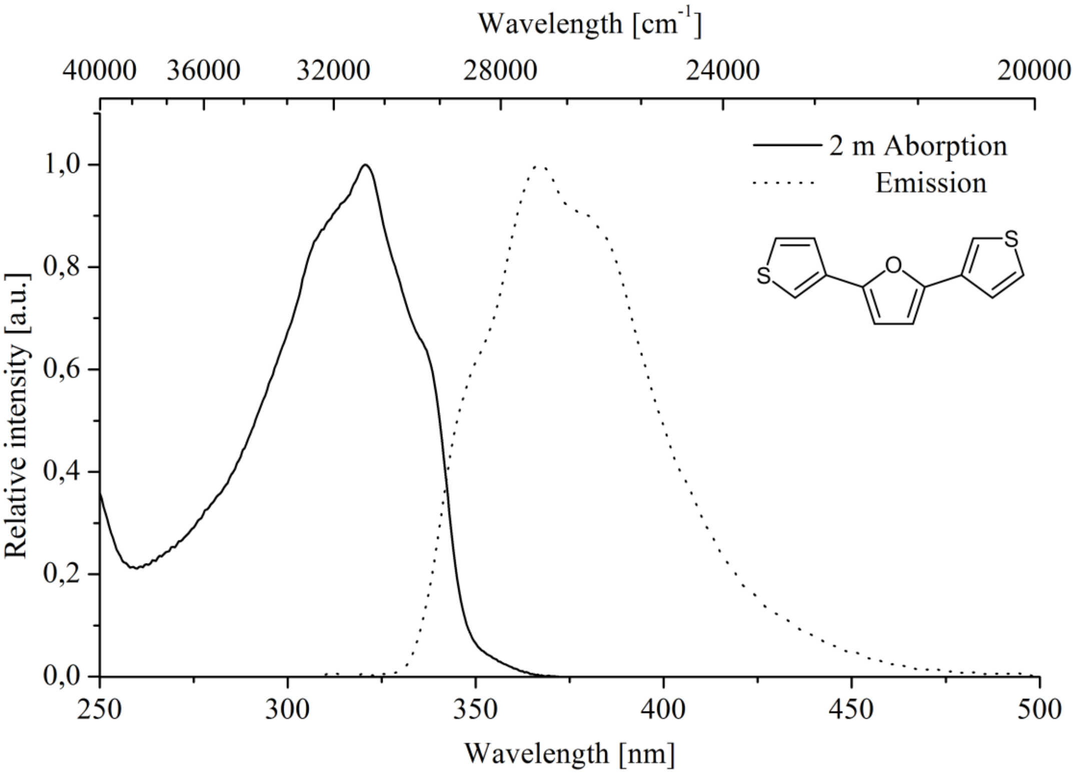

Normalized UV–vis and fluorescence spectra (recorded in dichloromethane at *T* = 20 °C).

2,5-Di(phenanthren-9-yl)furan (2n)

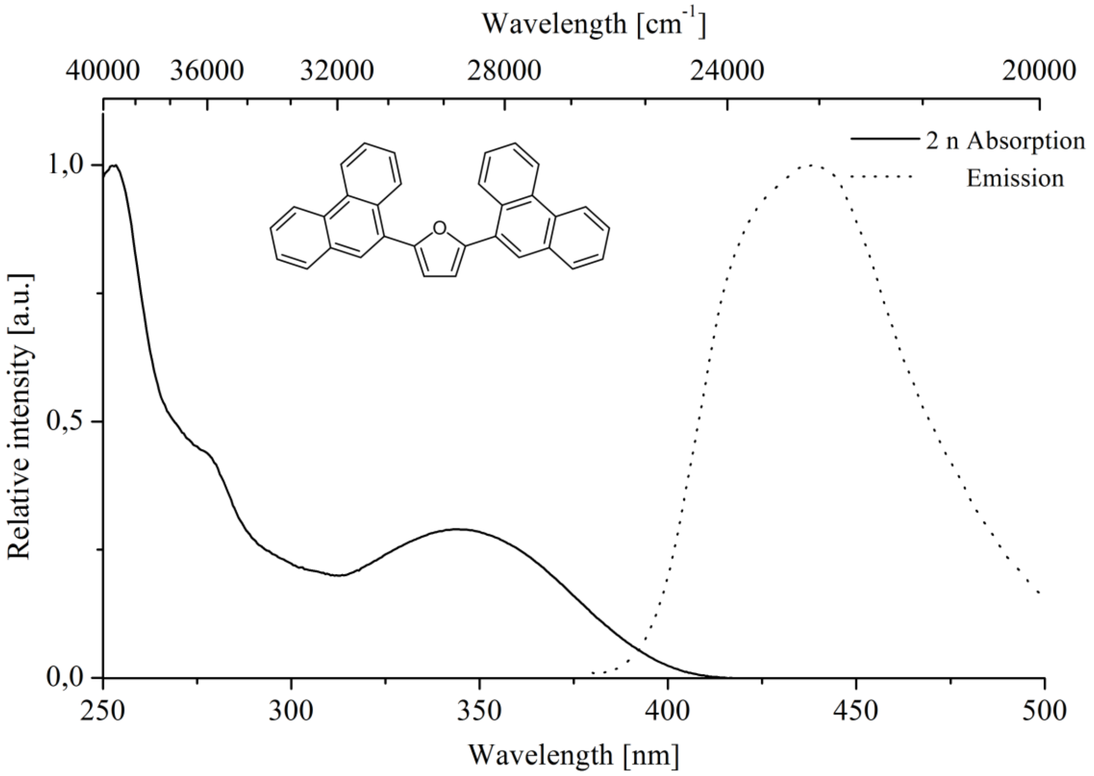

Normalized UV–vis and fluorescence spectra (recorded in dichloromethane at T = 20 °C).

2,2,5-bis(4-fluorophenyl)furan (2o)

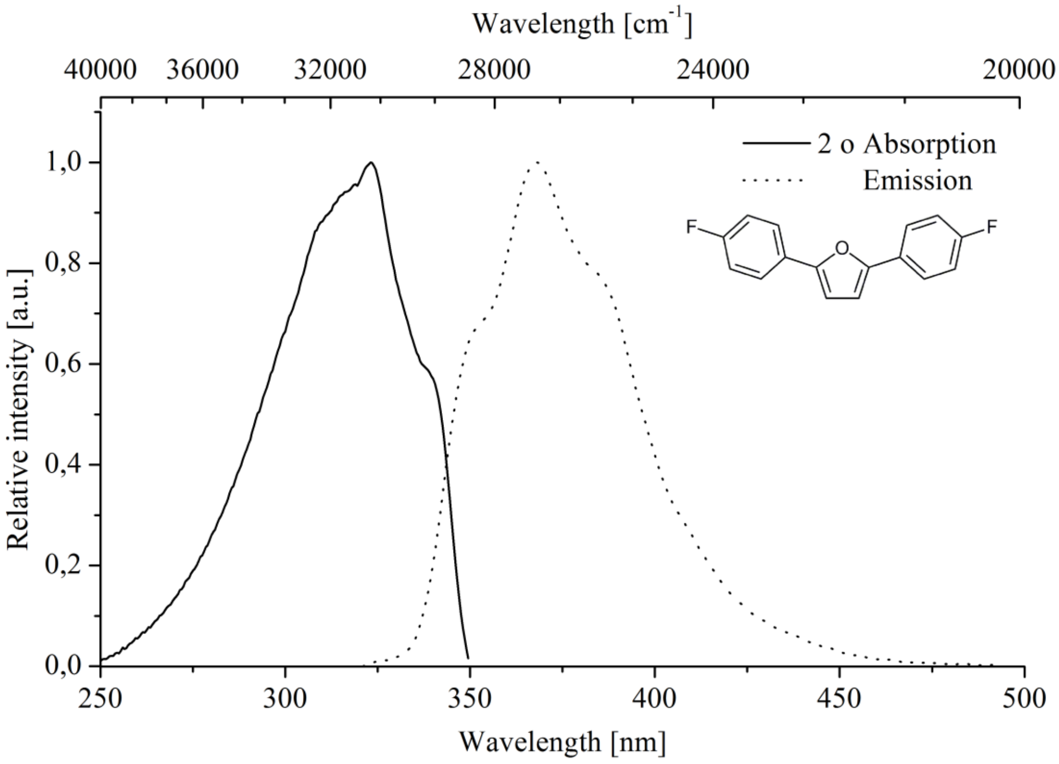

Normalized UV–vis and fluorescence spectra (recorded in dichloromethane at T = 20 °C).

6. NMR spectra of compounds 2a–2o

2,5-Diphenylfuran (2a)

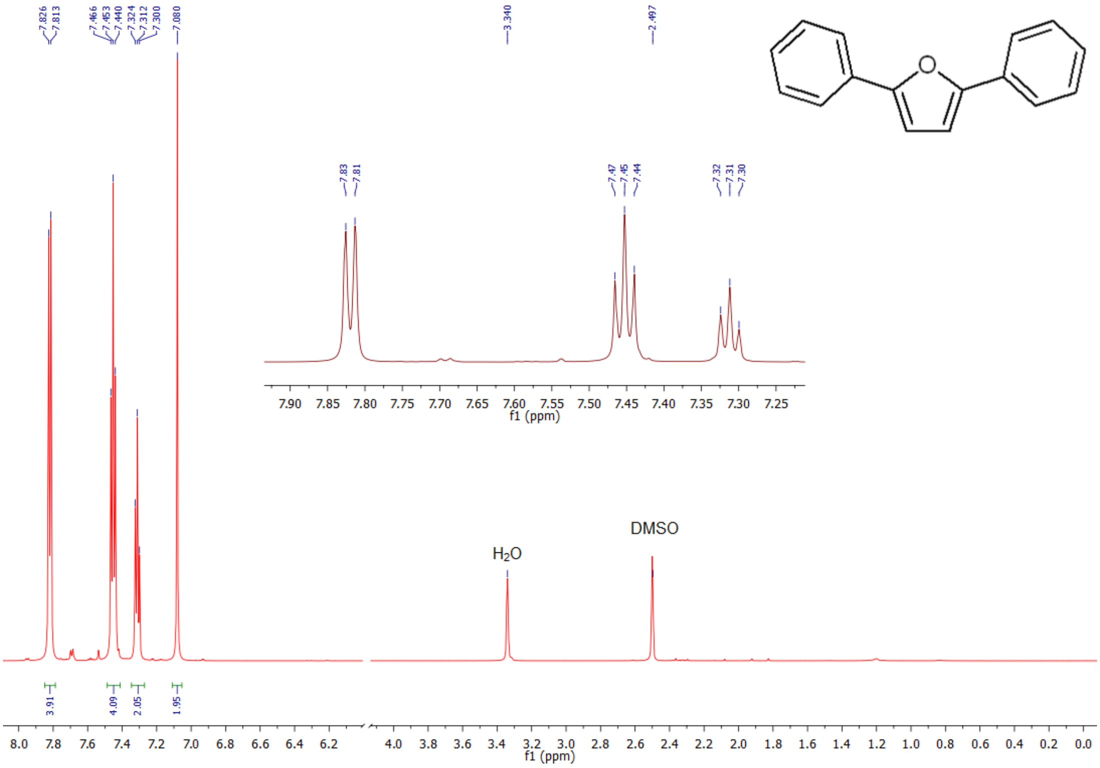

^{1}H NMR of **2a** in DMSO-d_6 at T = 298 K (600 MHz).

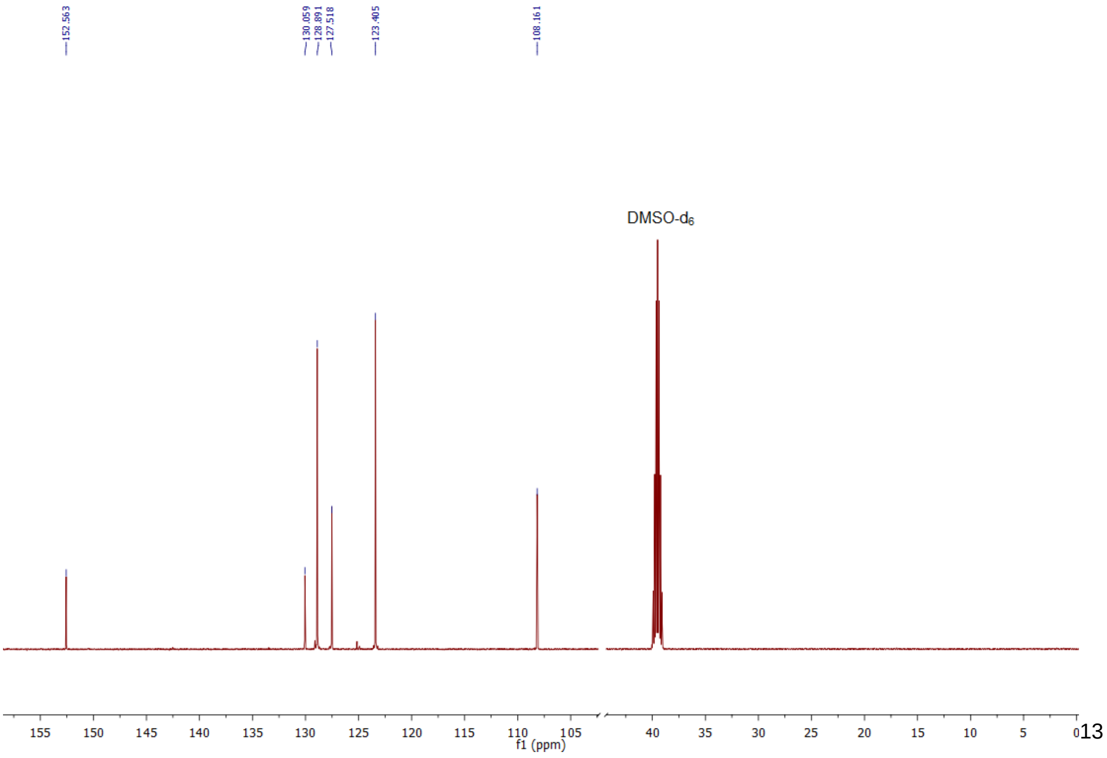

^{13}C NMR of **2a** in DMSO-d_6 at T = 298 K (150 MHz).

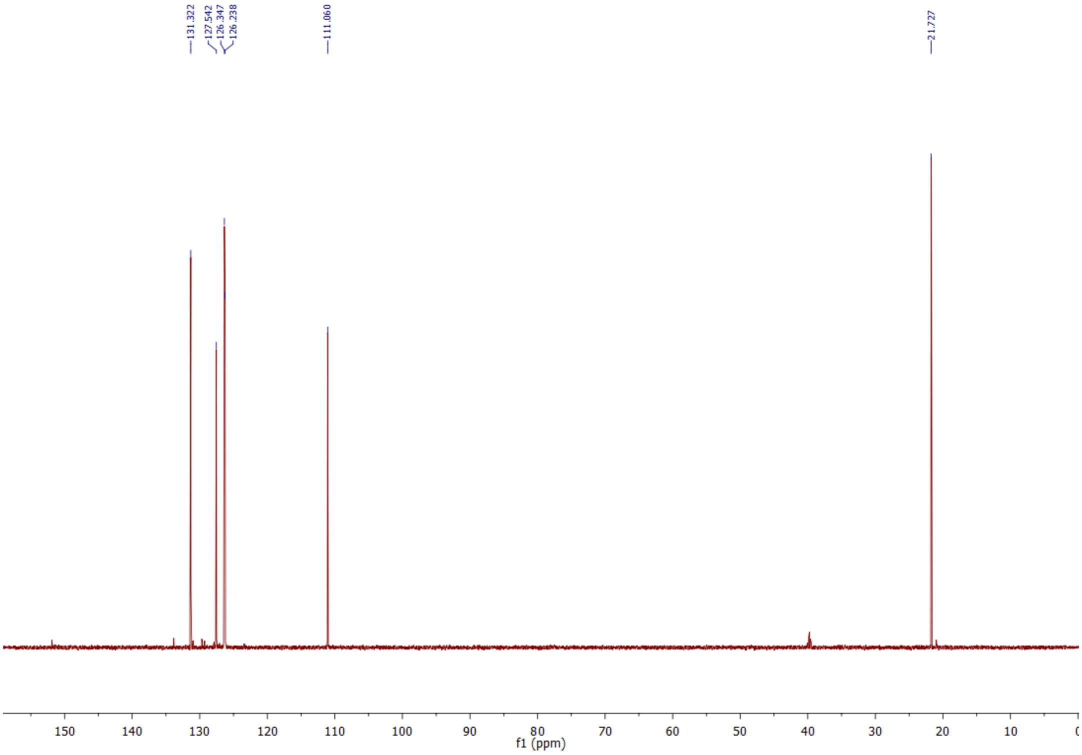

DEPT-135 NMR of **2a** in DMSO-d_6 at 298 K (150 MHz).

2,5-Di-*o*-tolylfuran (2b)

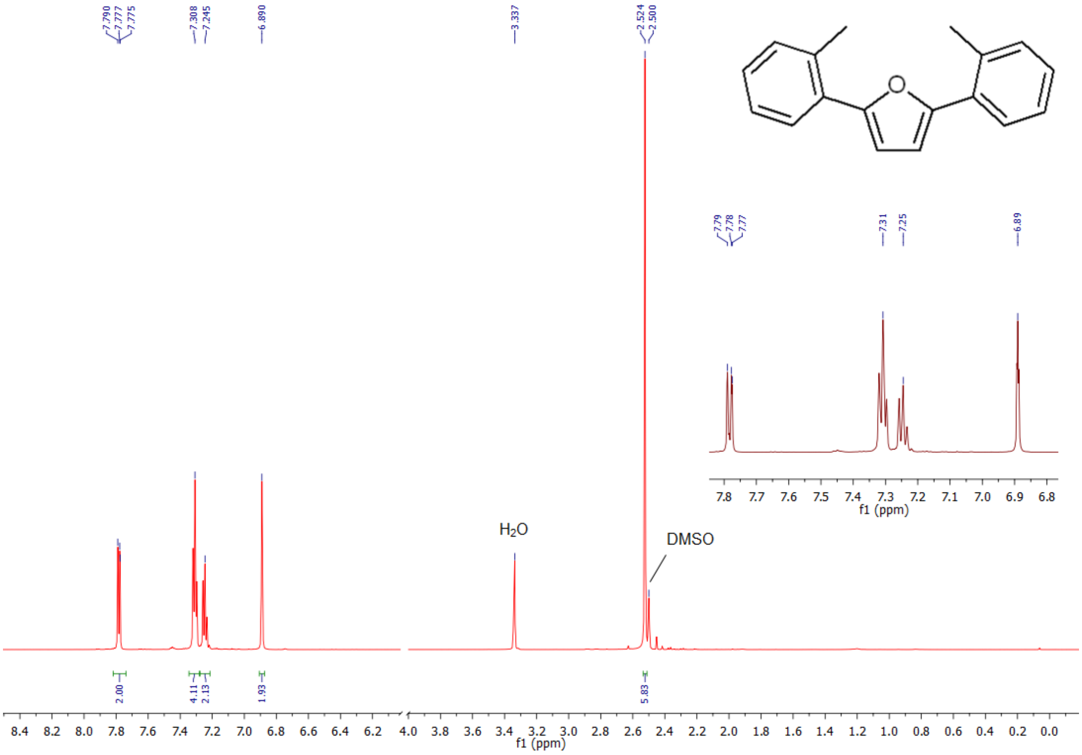

^{1}H NMR of **2b** in DMSO-d_6 at T = 298 K (600 MHz).

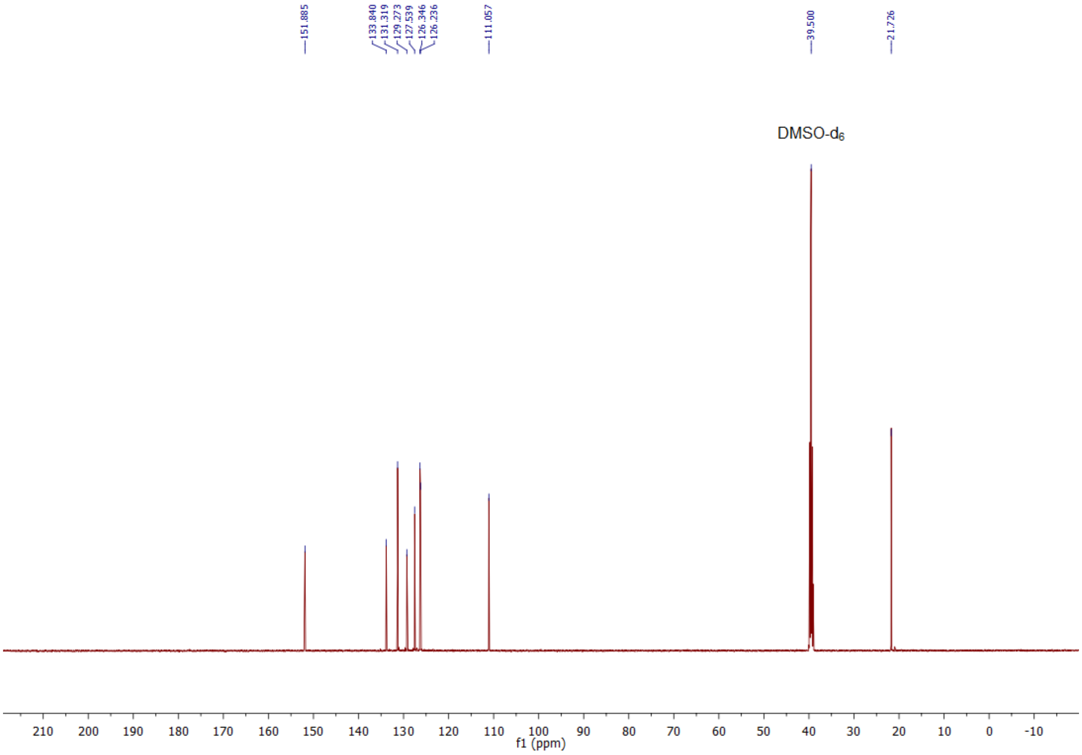

^{13}C NMR of **2b** in DMSO-d_6 at T = 298 K (150 MHz).

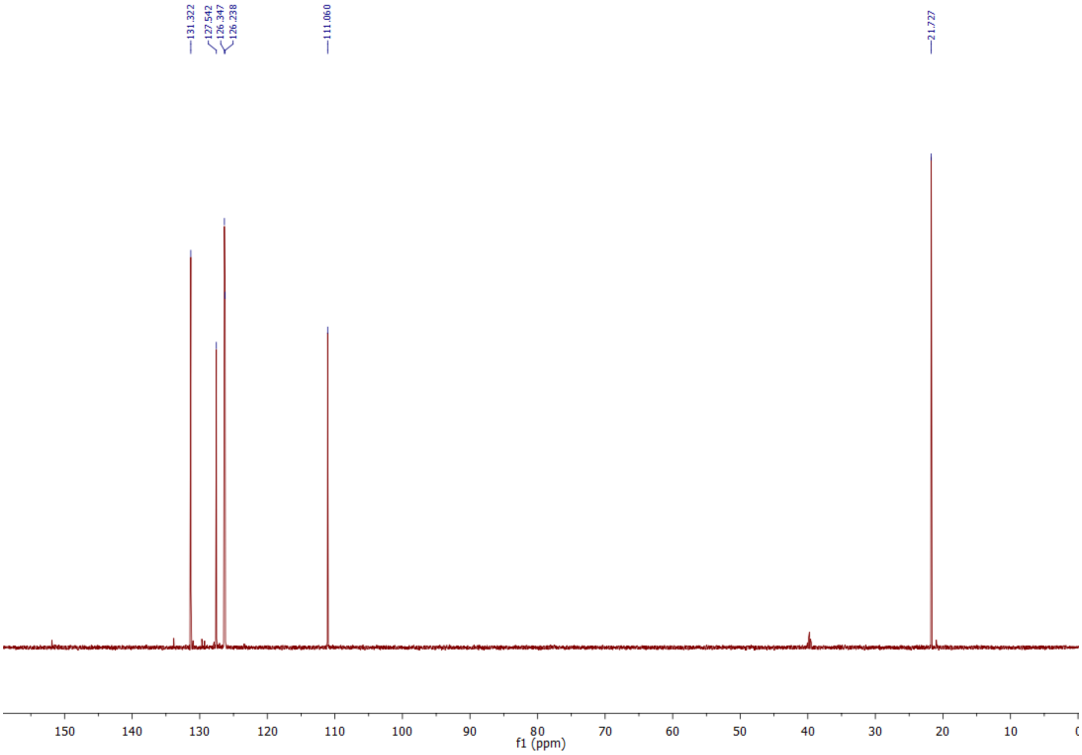

DEPT-135 NMR of **2b** in DMSO-d_6 at T = 298 K (150 MHz).

2,5-Di-*m*-tolylfuran (2c)

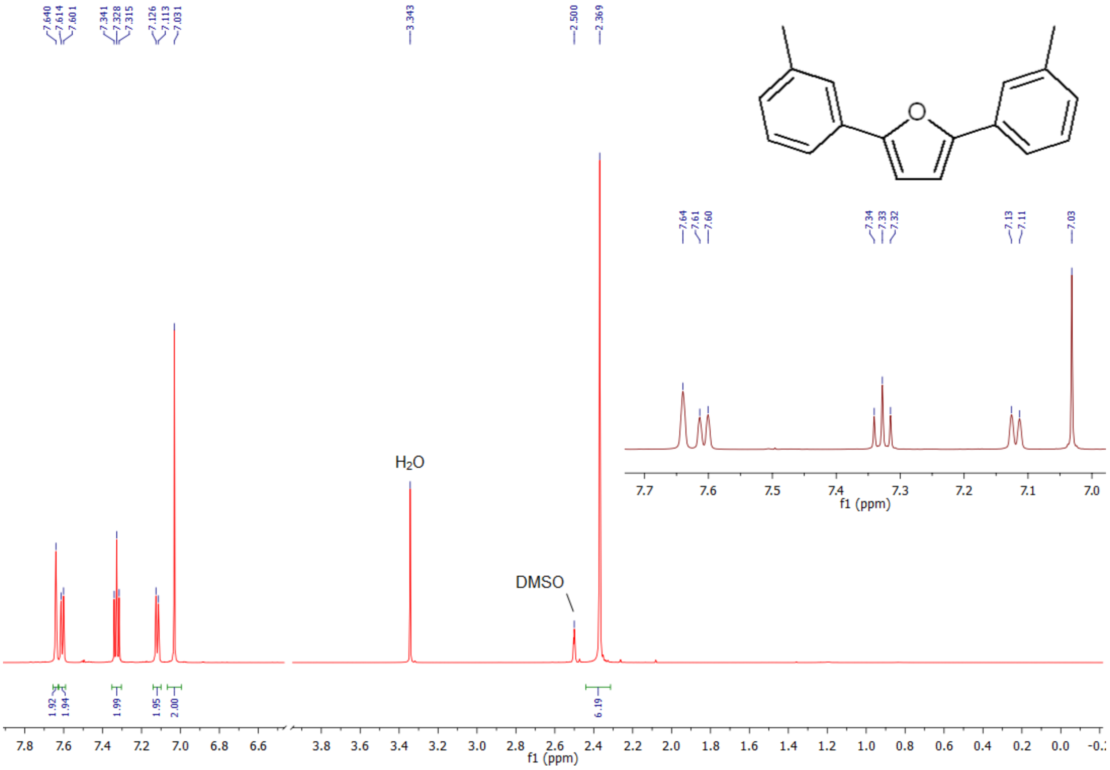

^{1}H NMR of **2c** in DMSO-d_6 at T = 298 K (600 MHz).

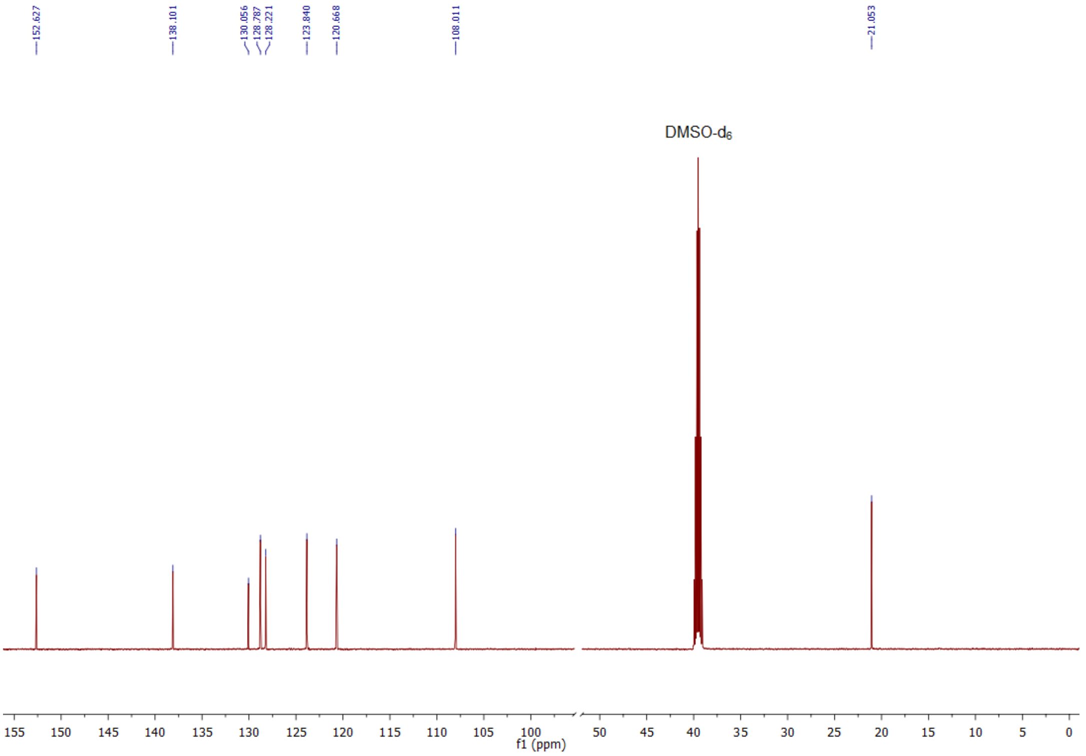

^{13}C NMR of **2c** in DMSO-d_6 at T = 298 K (150 MHz).

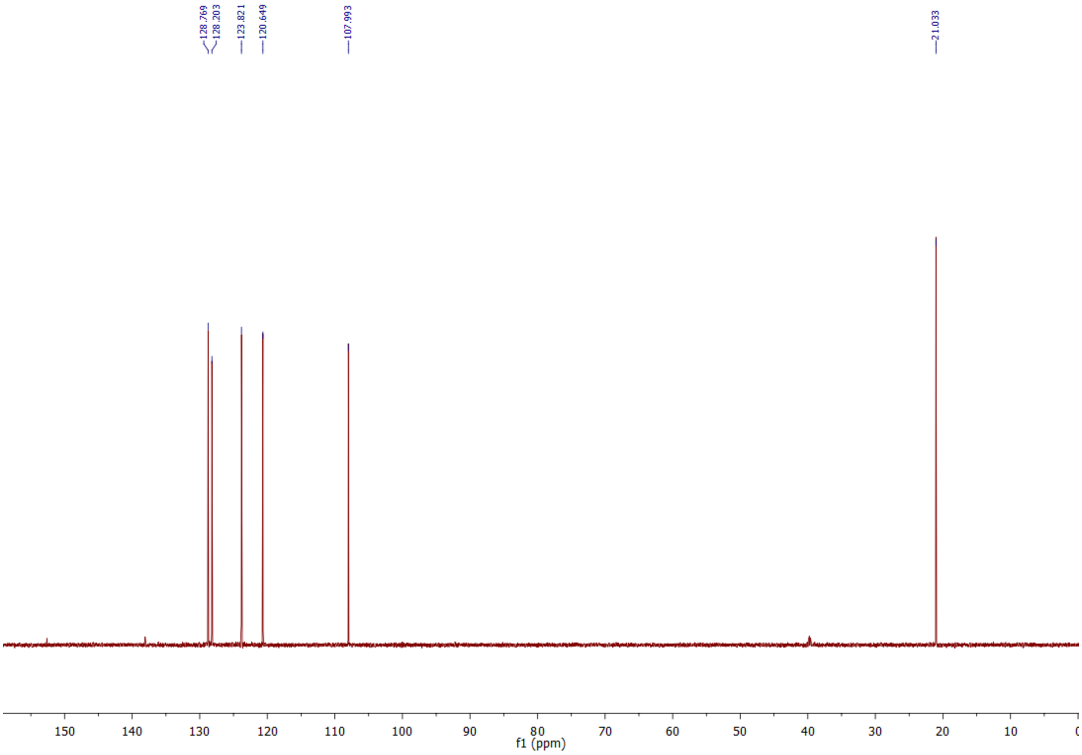

DEPT-135 NMR of **2c** in DMSO-d_6 at T = 298 K (150 MHz).

2,5-Di-*p*-tolylfuran (2d)

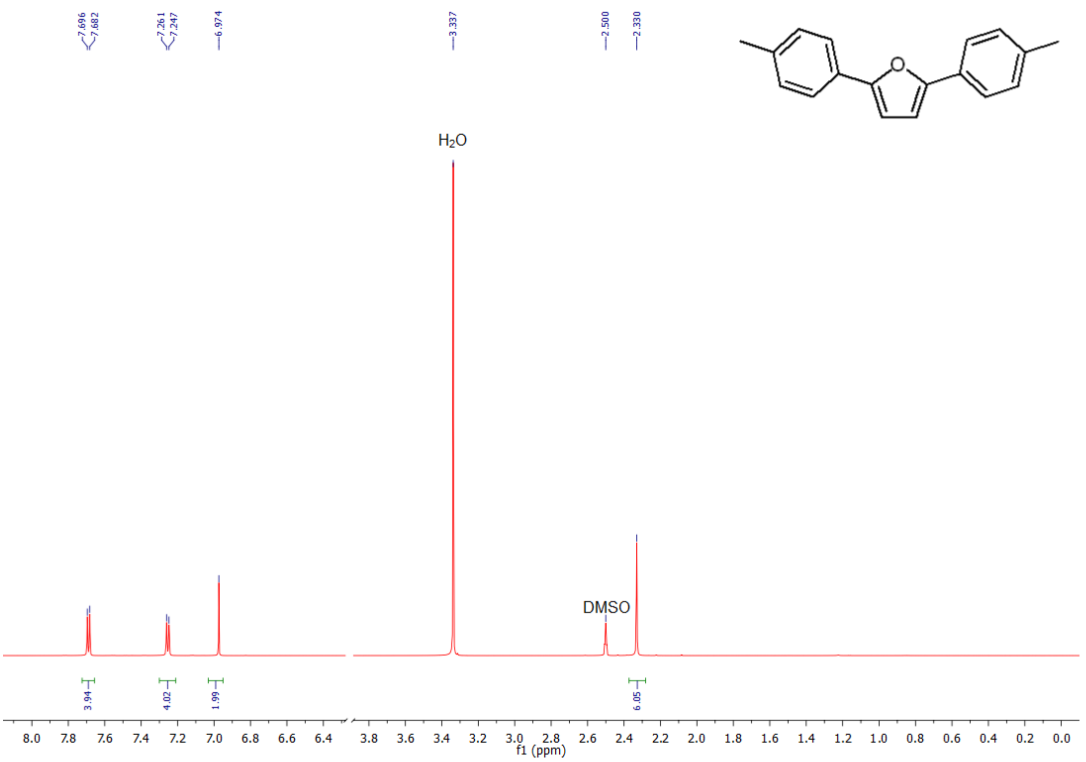

^{1}H NMR of **2d** in DMSO-d_6 at T = 298 K (600 MHz).

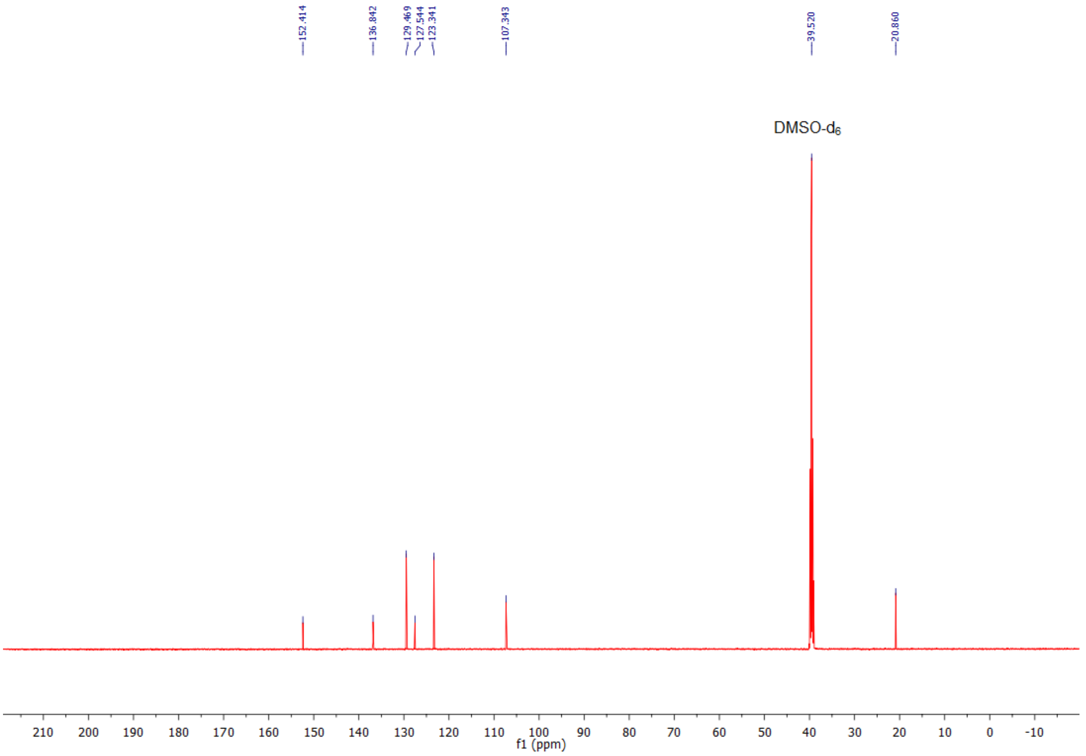

^{13}C NMR of **2d** in DMSO-d_6 at T = 298 K (150 MHz).

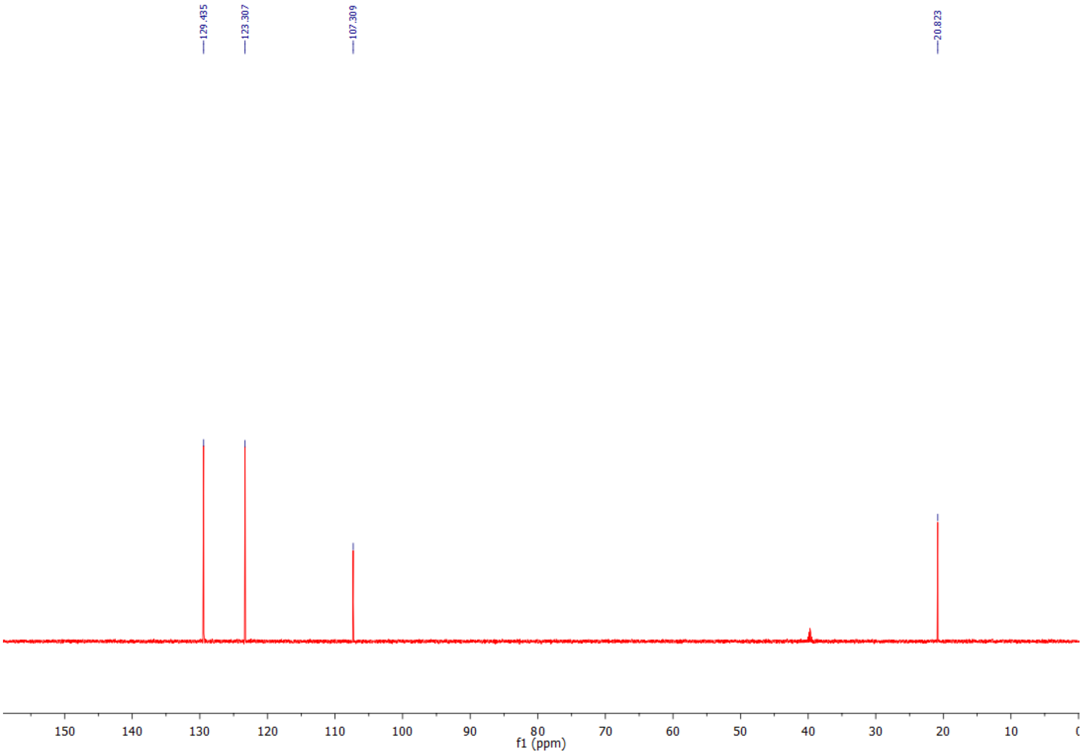

DEPT-135 NMR of **2d** in DMSO-d_6 at T = 298 K (150 MHz).

2,5-Bis(3,5-dimethylphenyl)furan (2e)

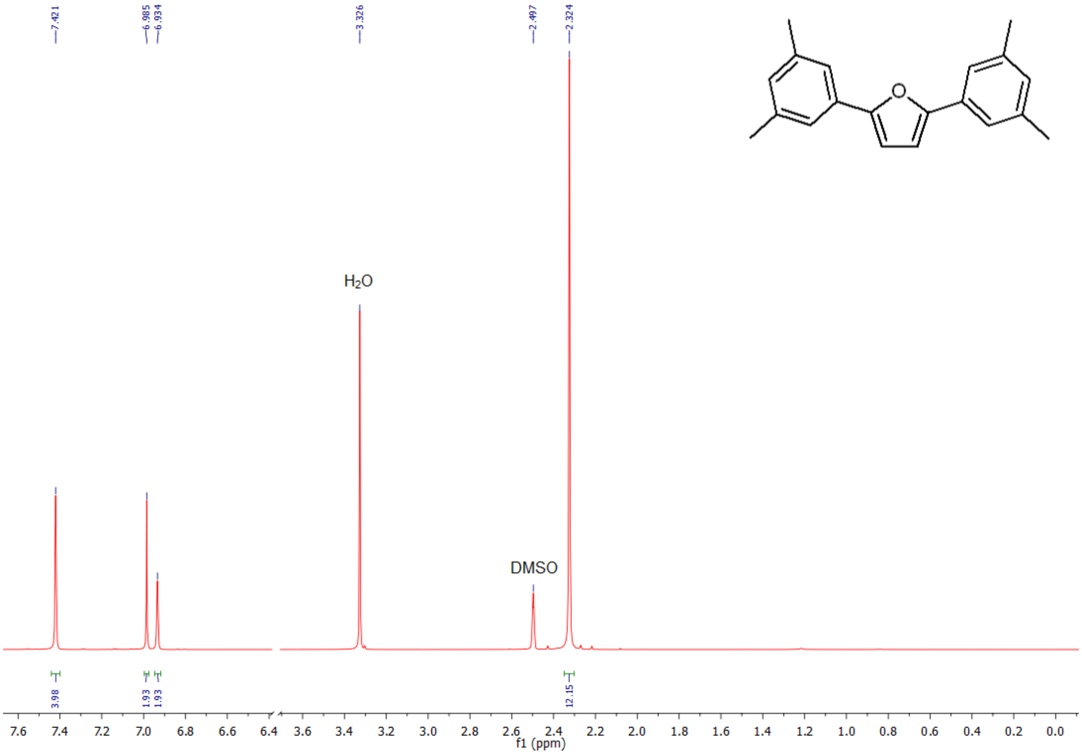

^{1}H NMR of **2e** in DMSO-d_6 at T = 298 K (600 MHz).

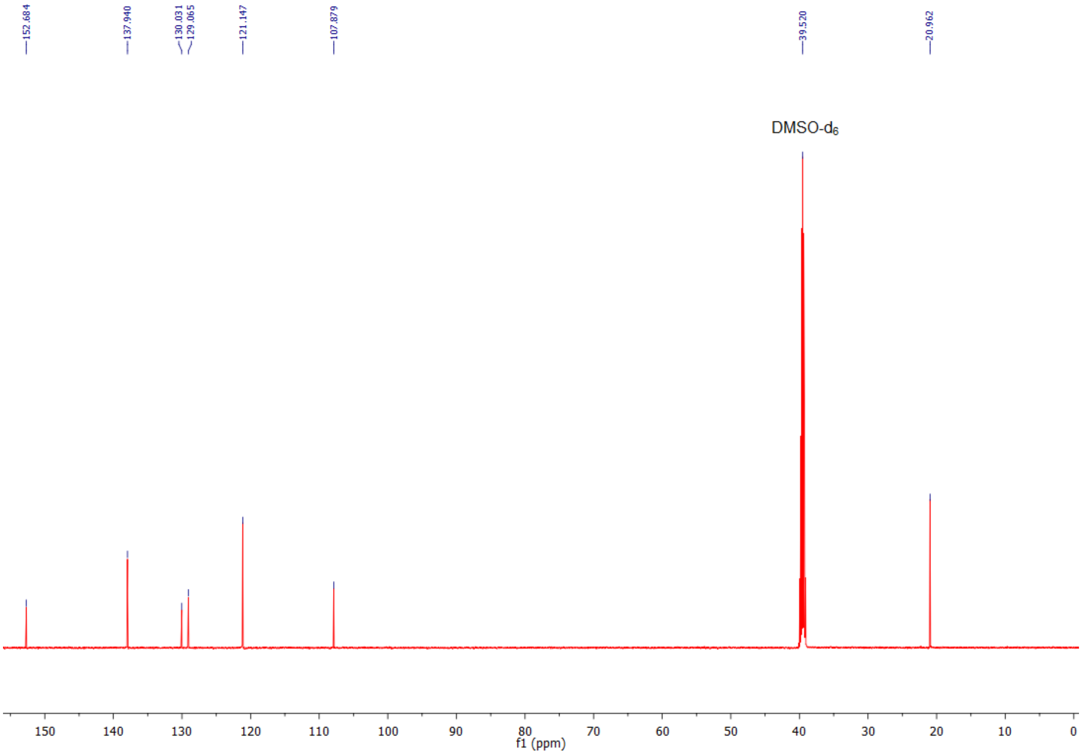

^{13}C NMR of **2e** in DMSO-d_6 at T = 298 K (150 MHz).

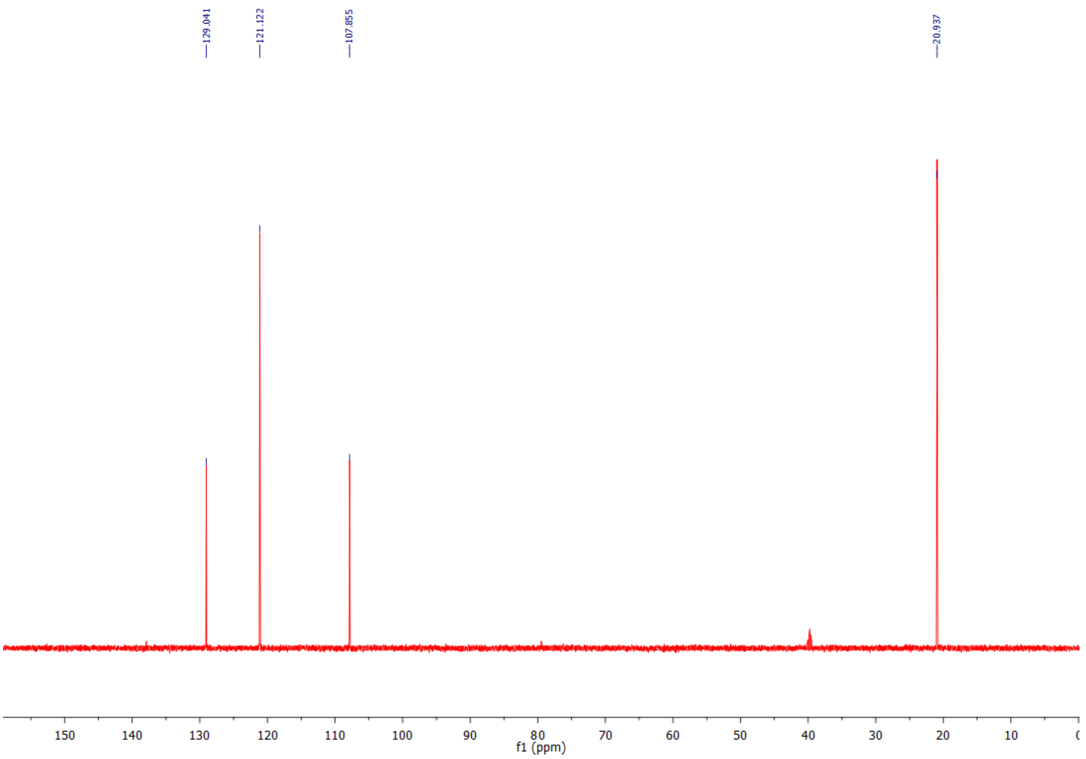

DEPT-135 NMR of **2e** in DMSO-d_6 at T = 298 K (150 MHz).

2,5-Bis(3-methoxyphenyl)furan (2f)

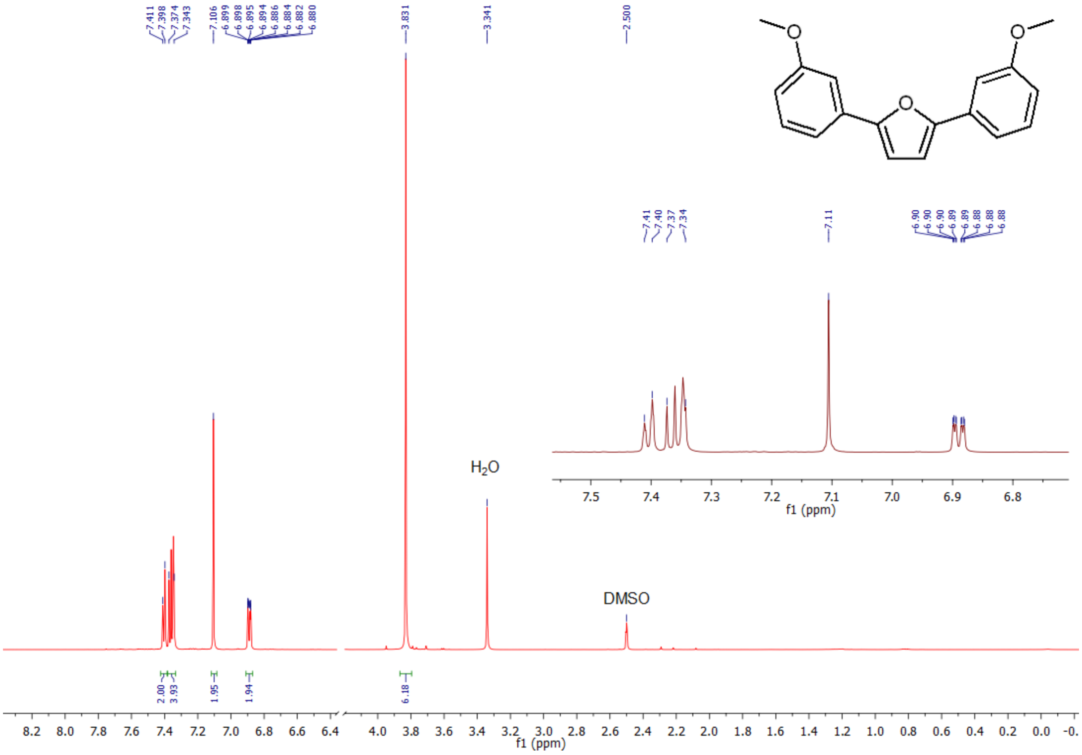

^{1}H NMR of **2f** in DMSO-d_6 at T = 298 K (600 MHz).

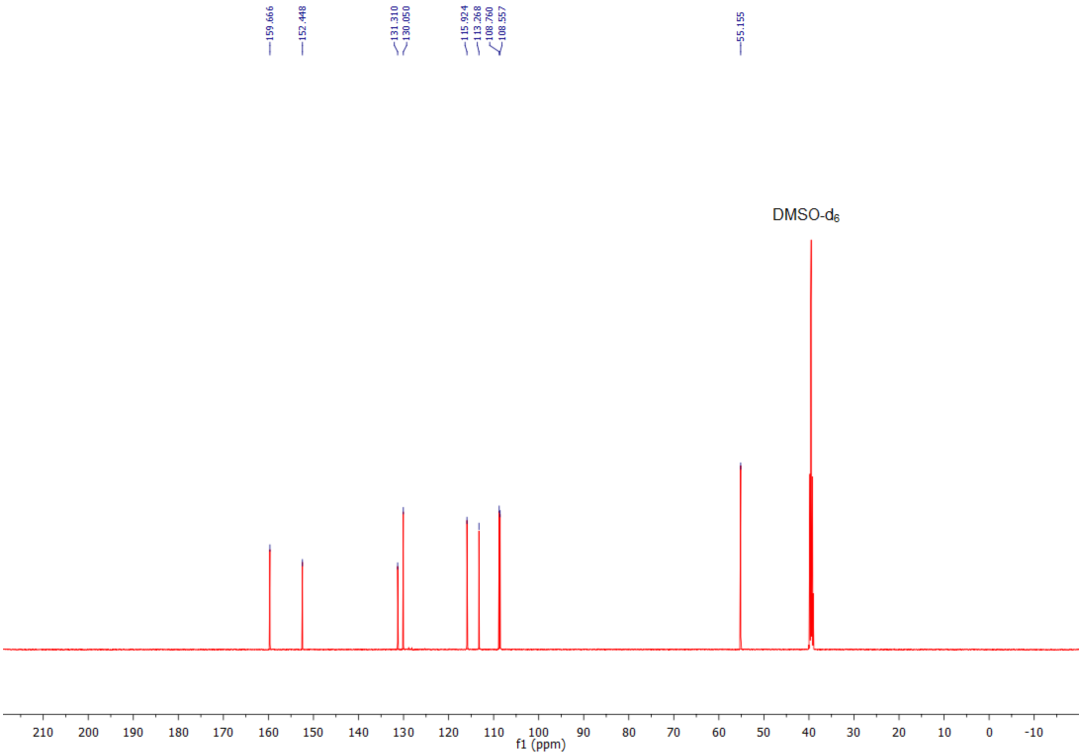

^{13}C NMR of **2f** in DMSO-d_6 at T = 298 K (150 MHz).

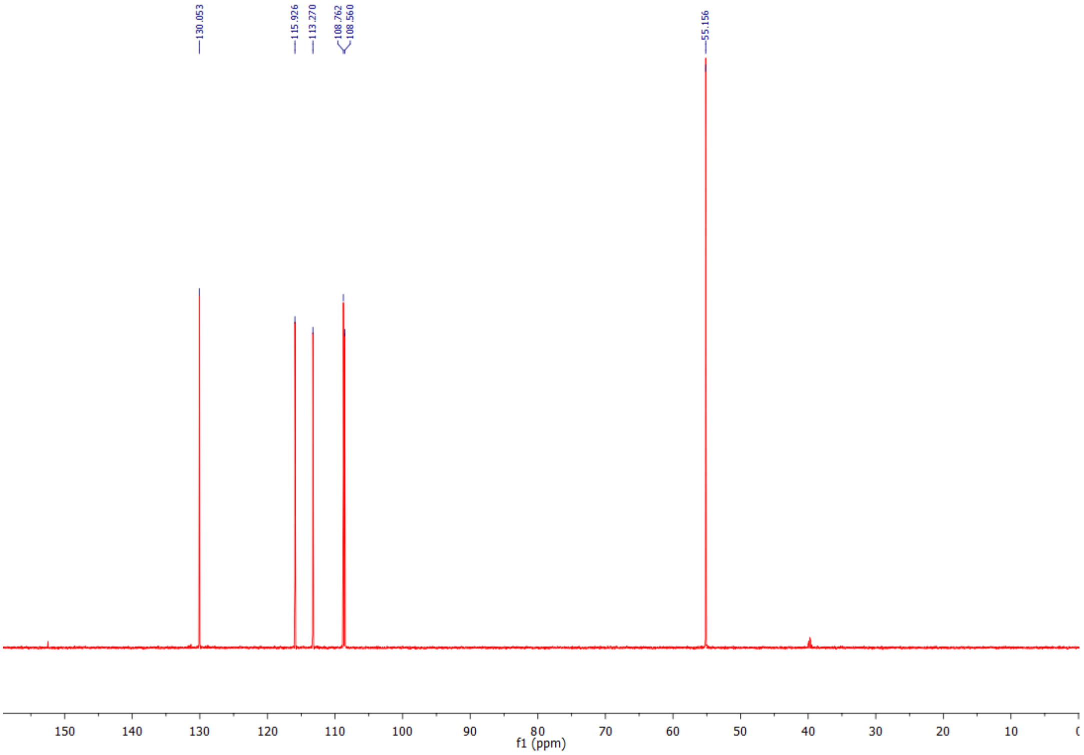

DEPT-135 NMR of **2f** in DMSO-d_6 at T = 298 K (150 MHz).

2,5-Bis(3,4,5-trimethoxyphenyl)furan (2g)

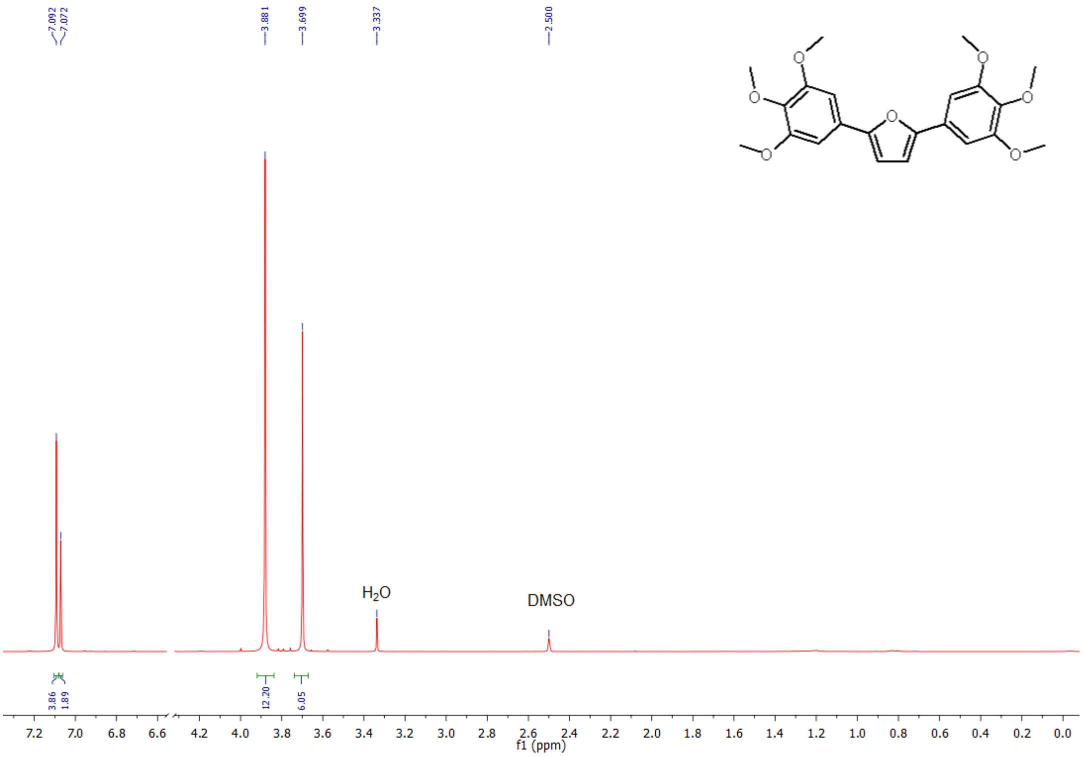

^{1}H NMR of **2g** in DMSO-d_6 at T = 298 K (600 MHz).

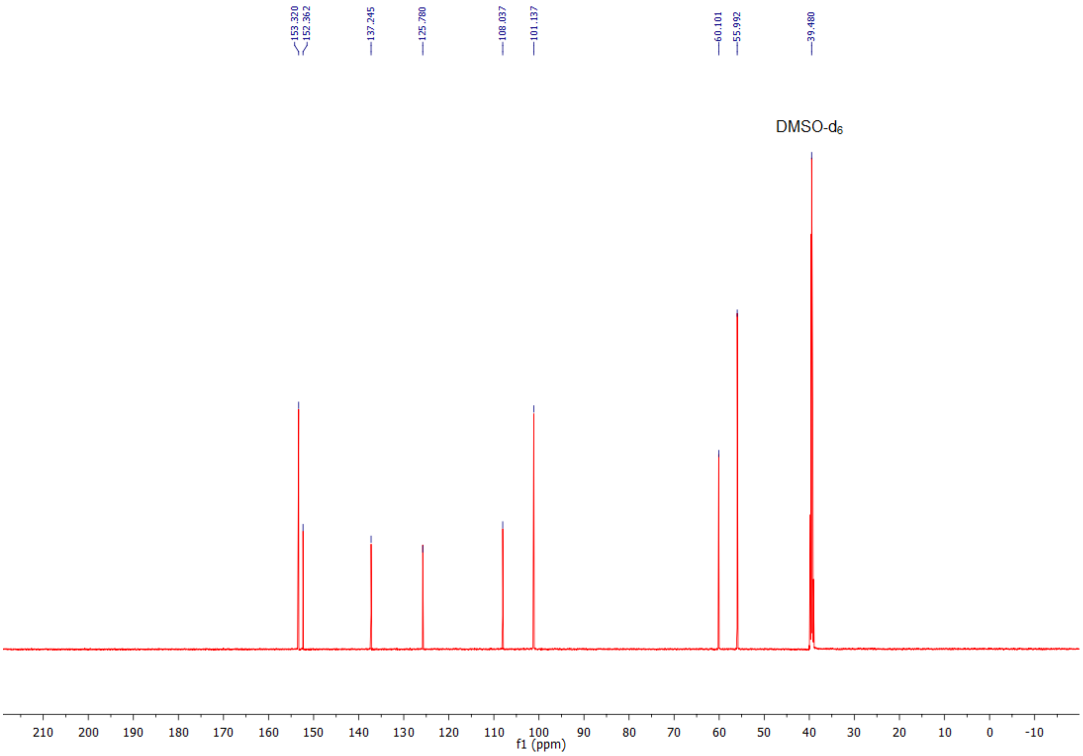

^{13}C NMR of **2g** in DMSO-d_6 at T = 298 K (150 MHz).

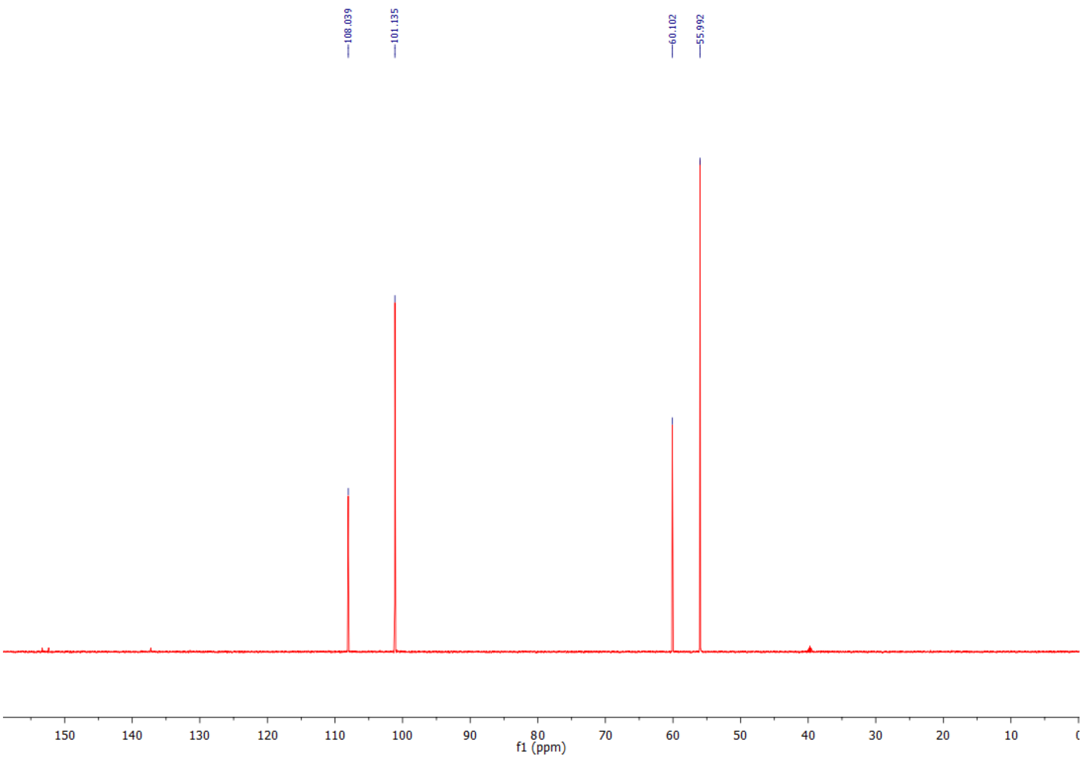

DEPT-135 NMR of **2g** in DMSO-d_6 at T = 298 K (150 MHz).

2,5-Bis(2-methoxy-5-methylphenyl)furan (2h)

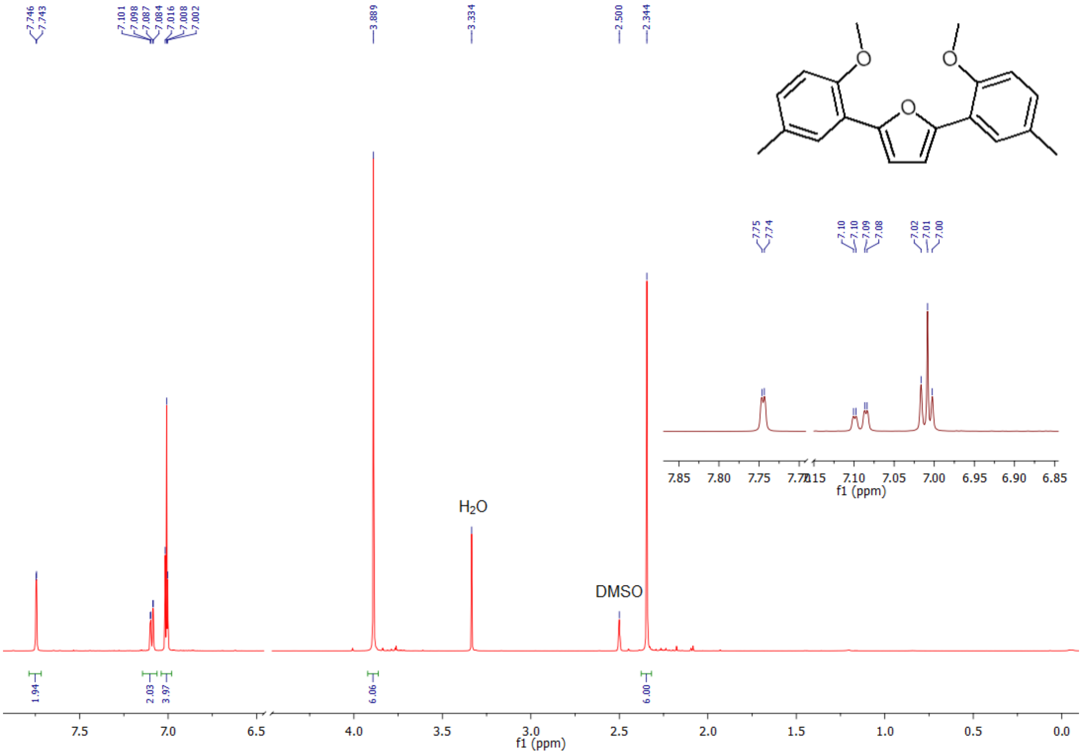

^{1}H NMR of **2h** in DMSO-d_6 at T = 298 K (600 MHz).

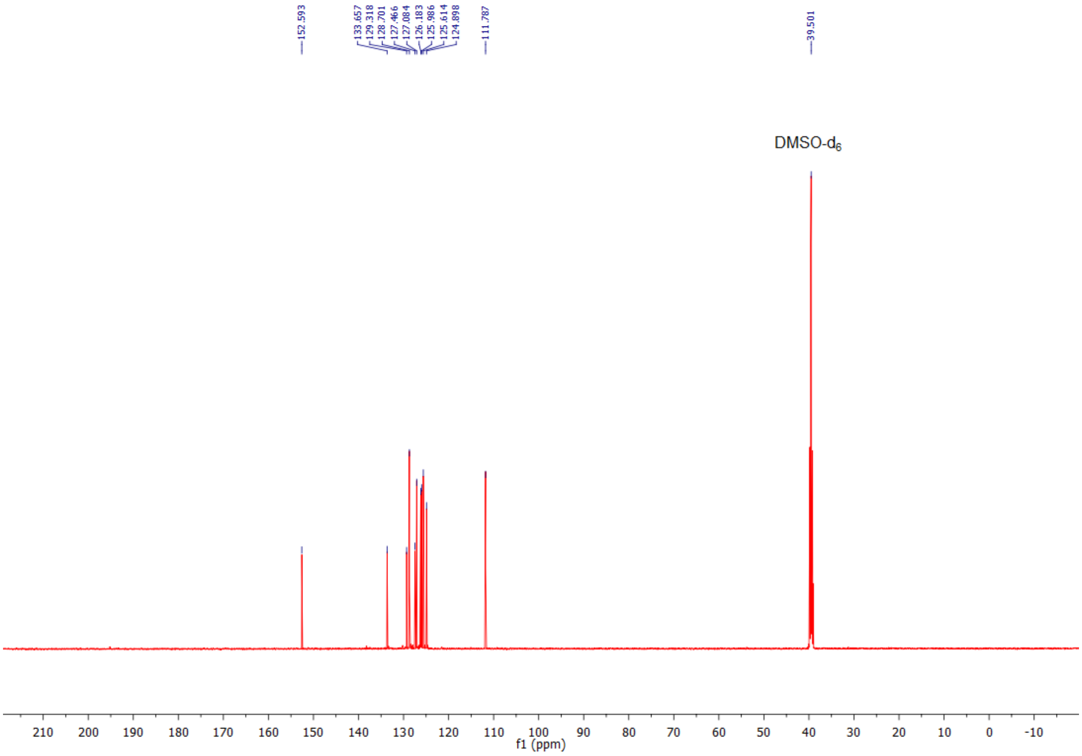

^{13}C NMR of **2h** in DMSO-d_6 at T = 298 K (150 MHz).

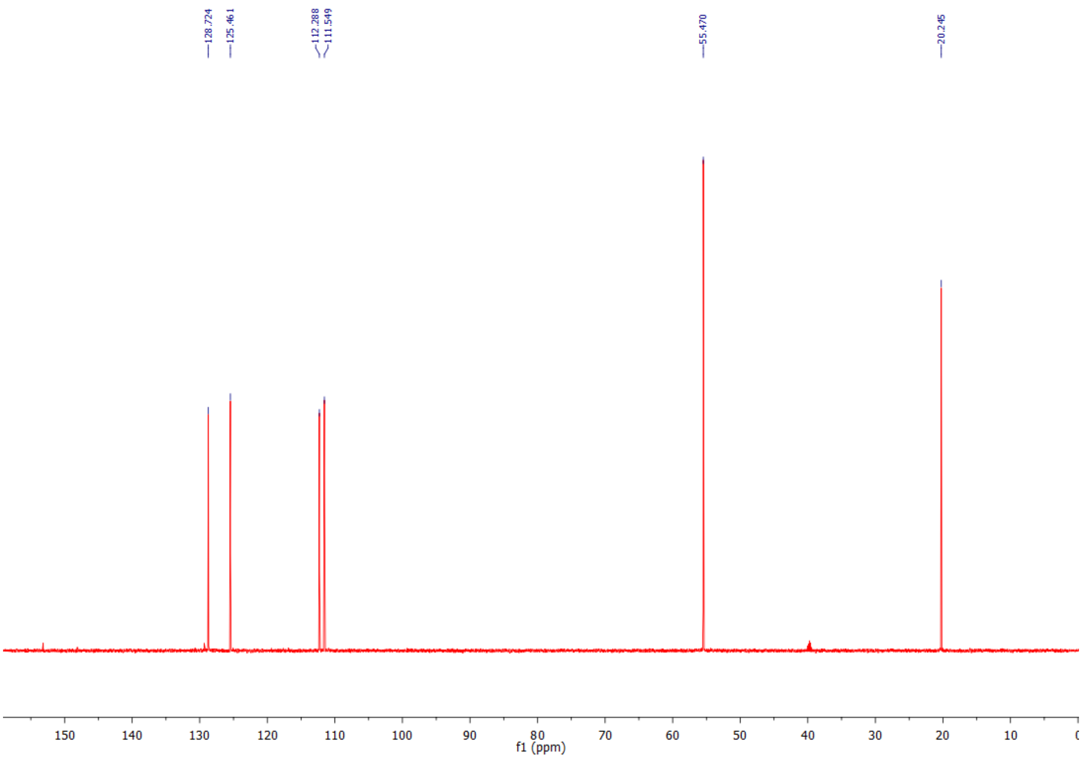

DEPT-135 NMR of **2h** in DMSO-d_6 at T = 298 K (150 MHz).

2,5-Bis(naphthalen-1-yl)furan (2i)

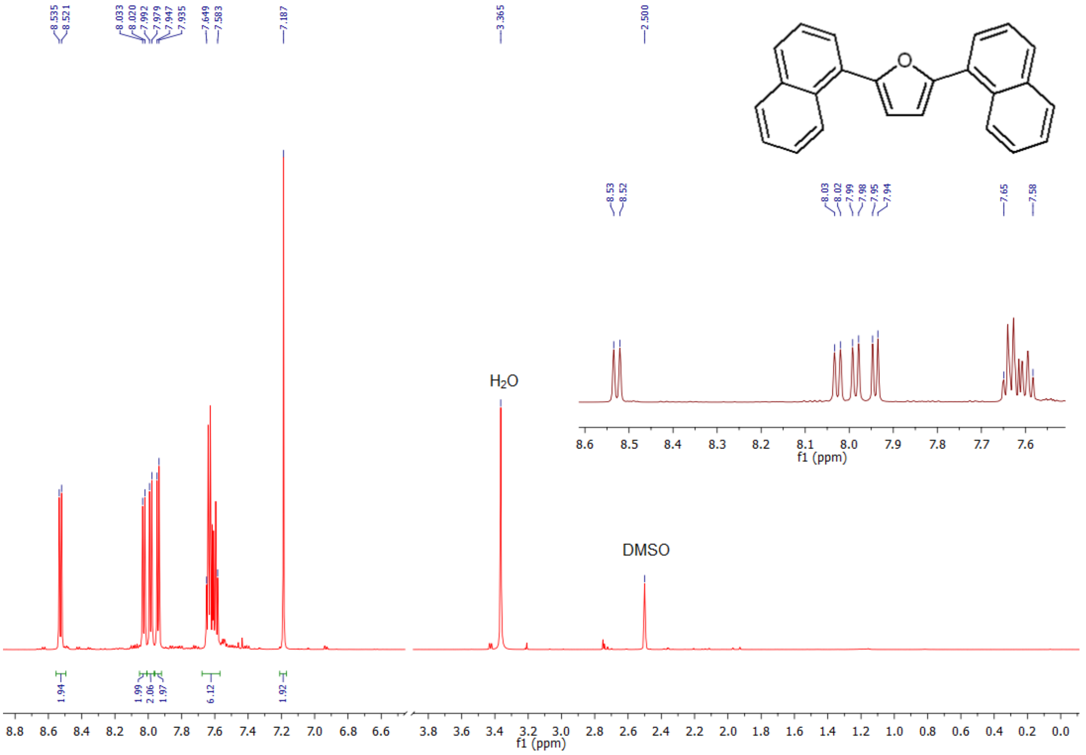

^{1}H NMR of **2i** in DMSO-d_6 at T = 298 K (600 MHz).

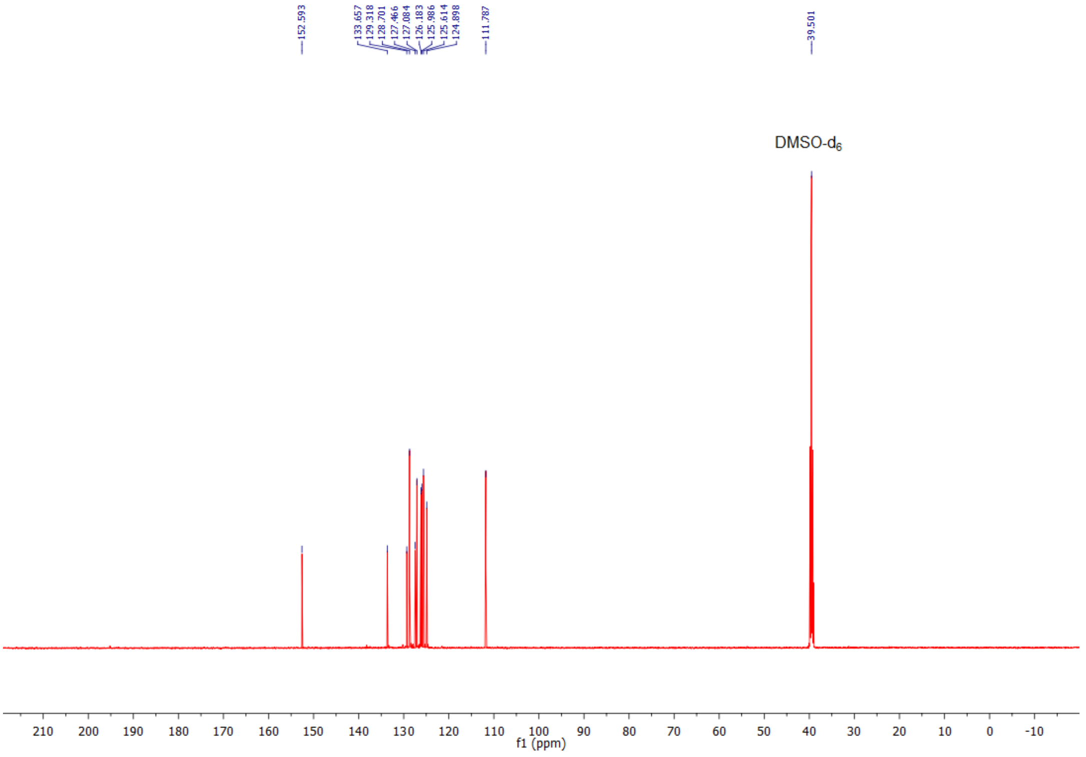

^{13}C NMR of **2i** in DMSO-d_6 at T = 298 K (150 MHz).

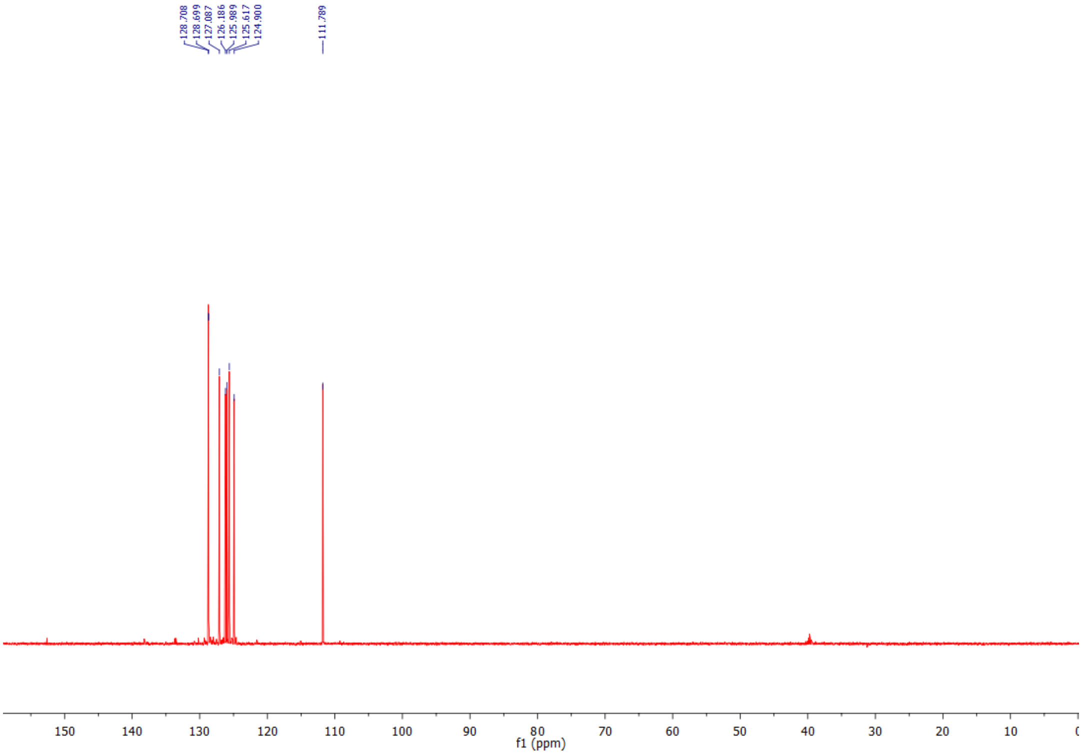

DEPT-135 NMR of **2i** in DMSO-d_6 at T = 298 K (150 MHz).

2,5-Bis(naphthalen-2-yl)furan (2j)

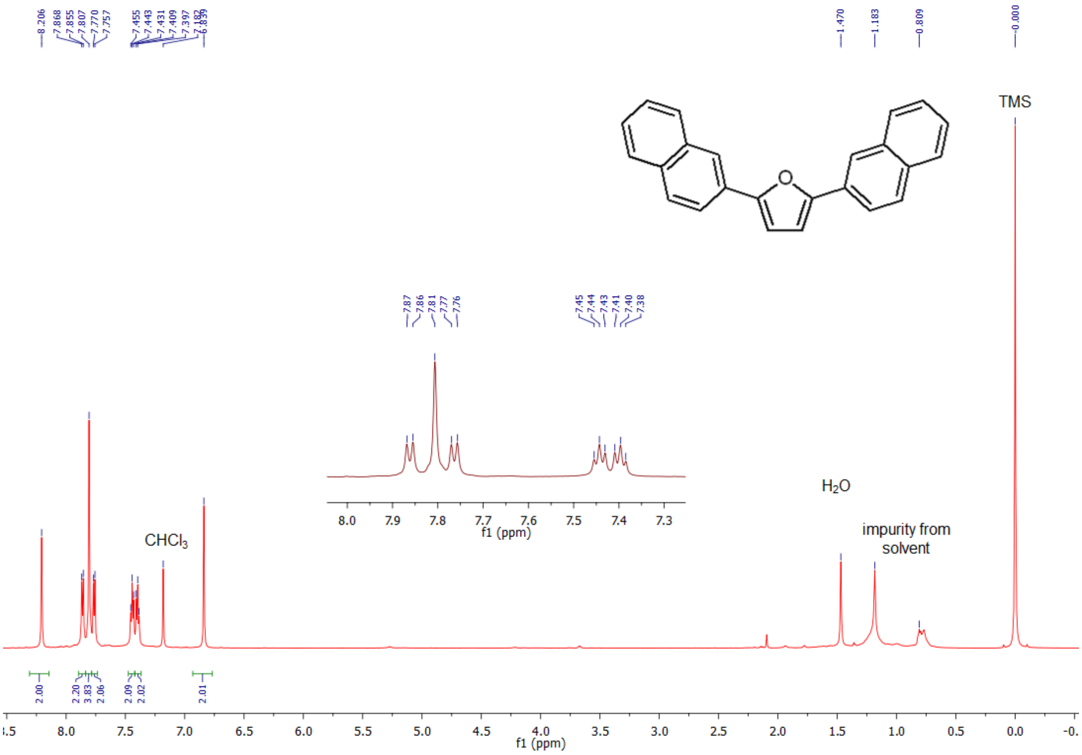

^{1}H NMR of **2j** in $CDCl_3$ at T = 298 K (600 MHz).

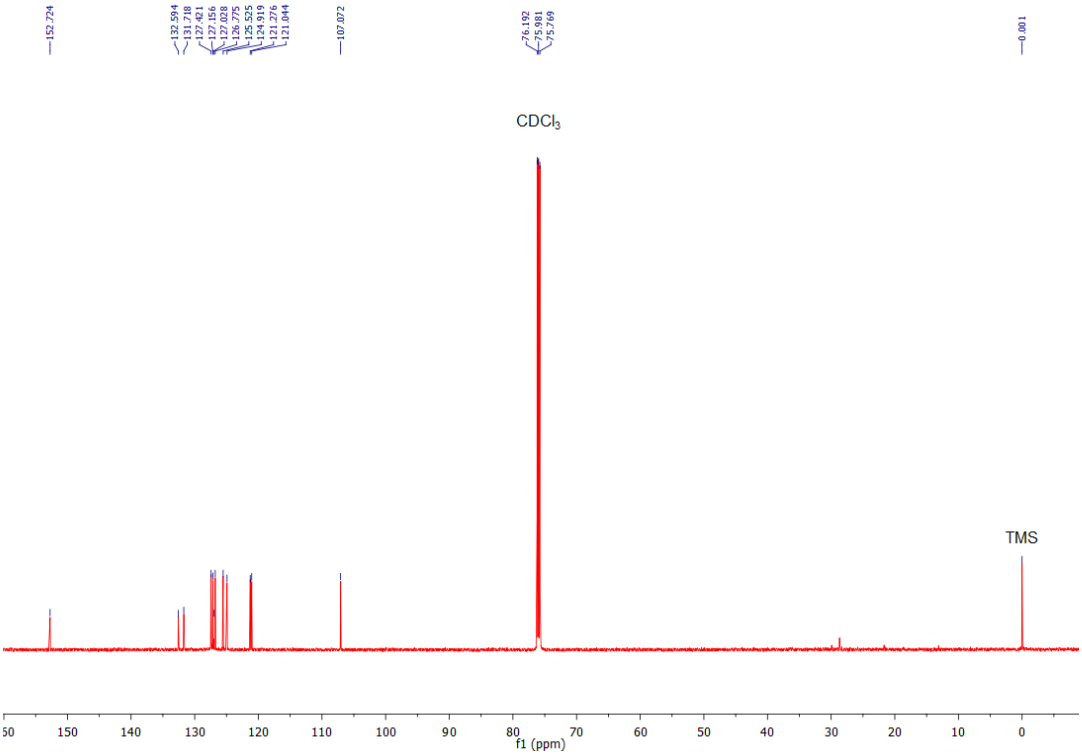

^{13}C NMR of **2j** in $CDCl_3$ at T = 298 K (150 MHz).

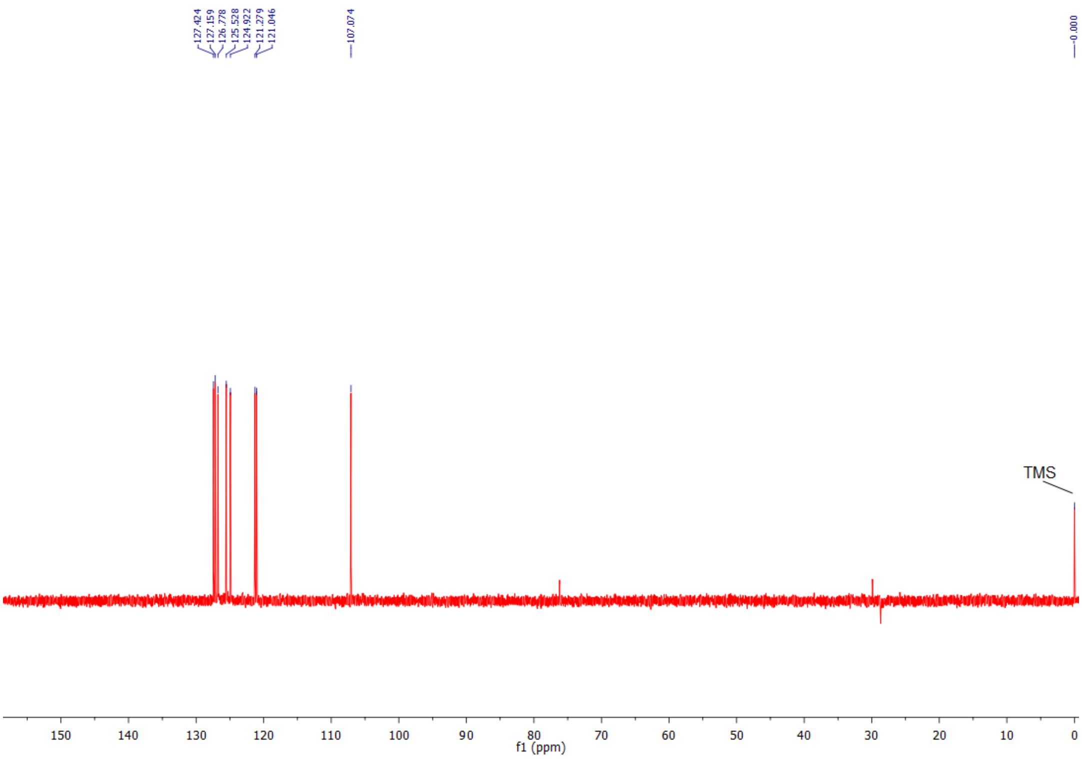

DEPT-135 NMR of **2j** in $CDCl_3$ at T = 298 K (150 MHz).

2,5-Bis(3-hydroxymethylphenyl)furan (2k)

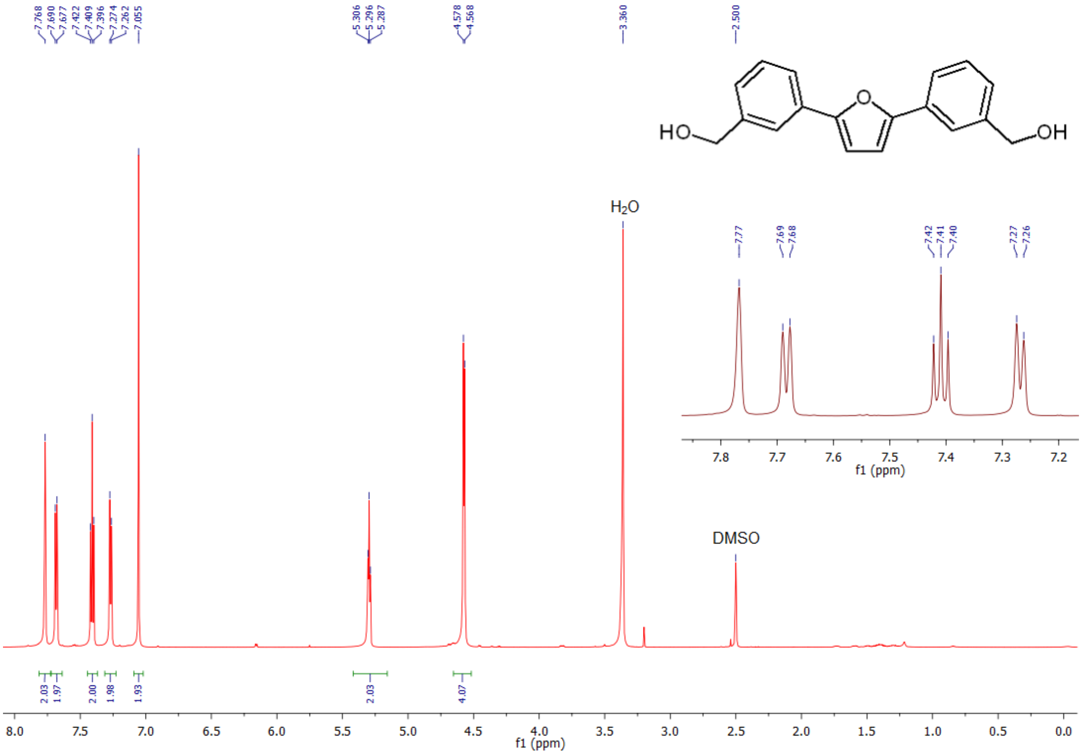

^{1}H NMR of **2k** in DMSO-d_6 at T = 298 K (600 MHz).

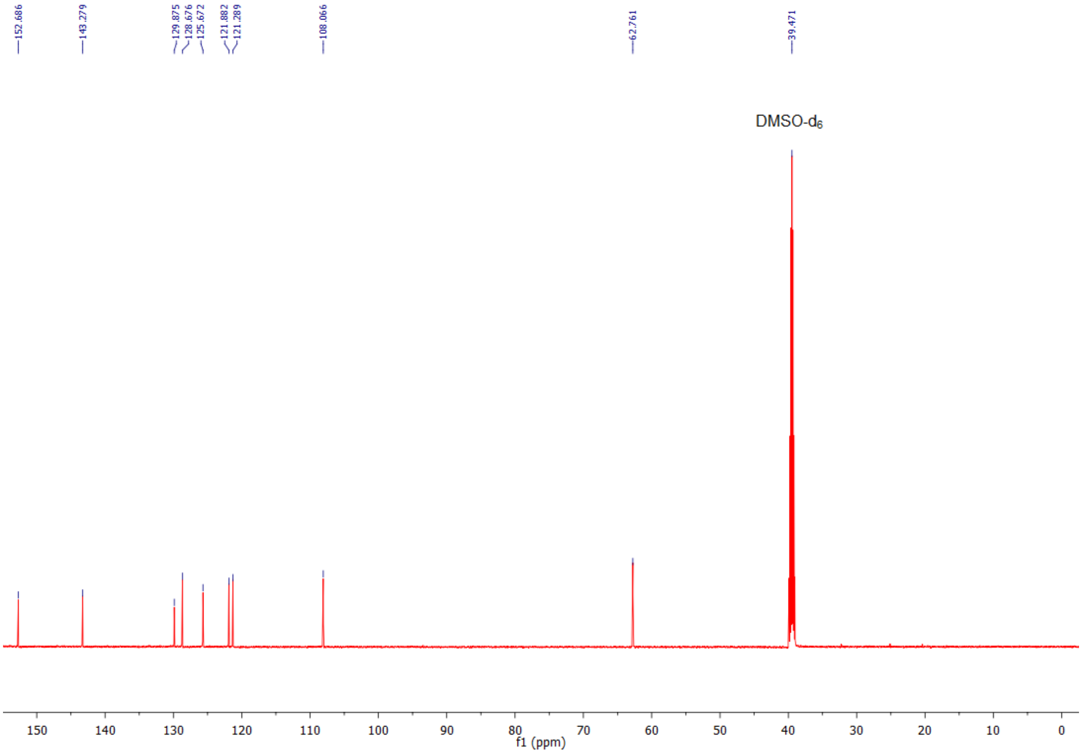

^{13}C NMR of **2k** in DMSO-d_6 at T = 298 K (150 MHz).

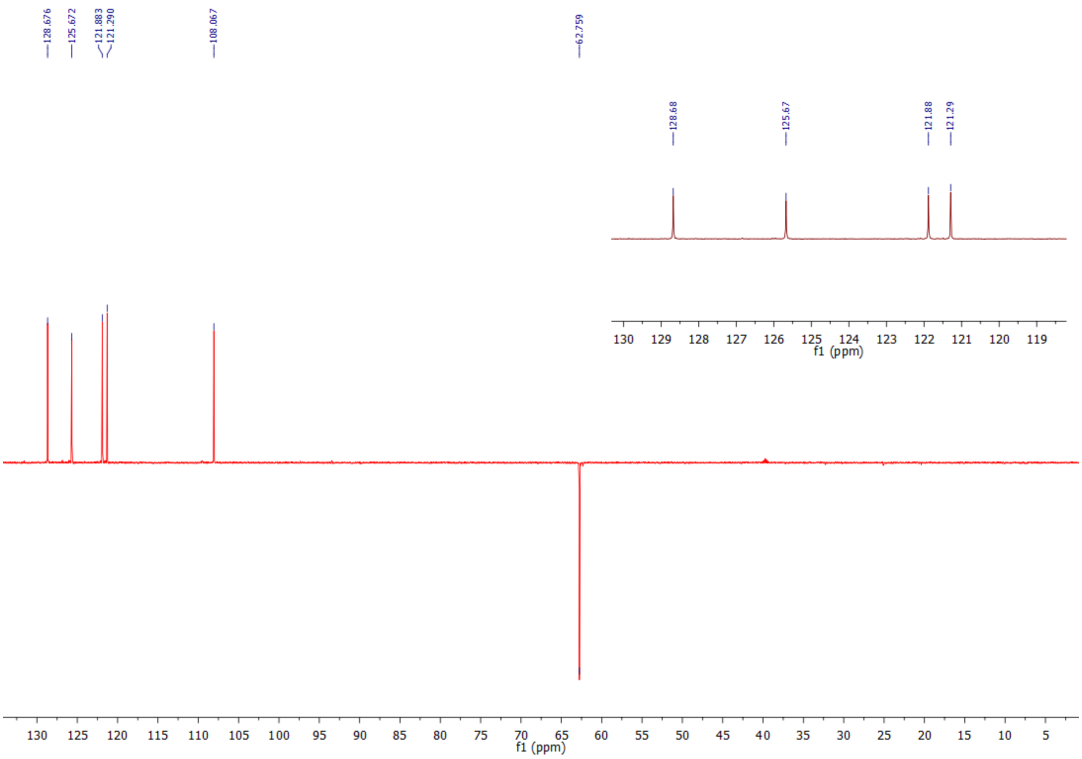

DEPT-135 NMR of **2k** in DMSO-d_6 at T = 298 K (150 MHz).

2,5-Bis(thiophen-2-yl)furan (2l)

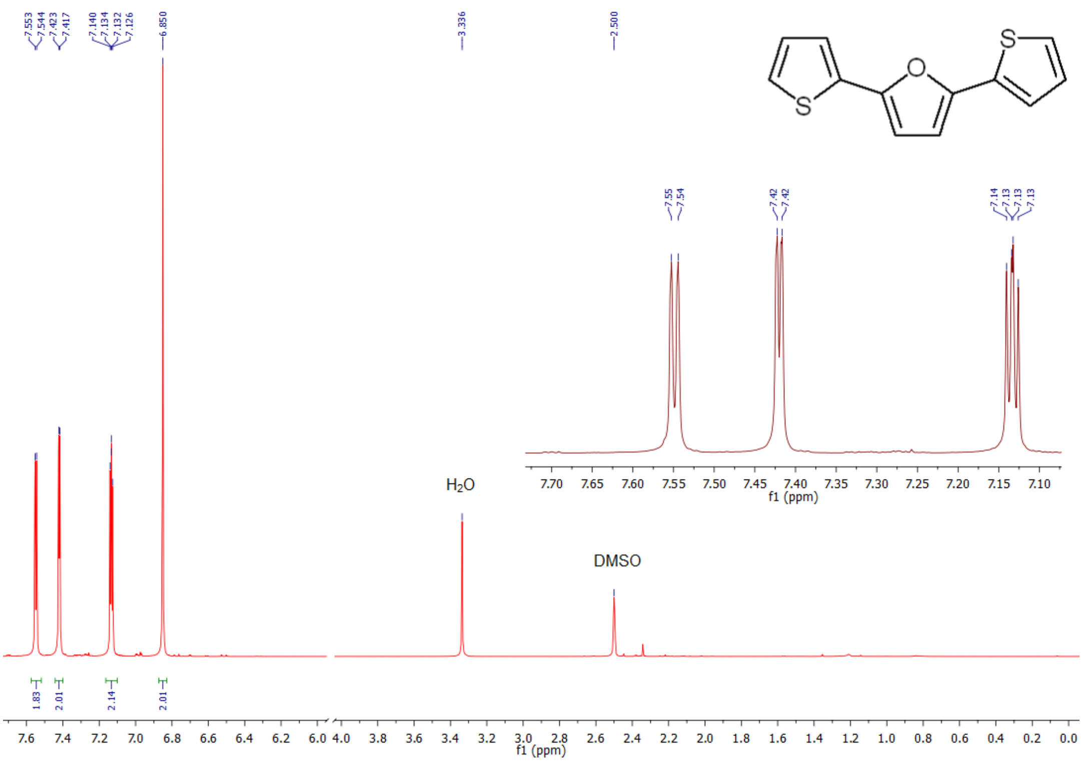

^{1}H NMR of **2l** in DMSO-d_6 at T = 298 K (600 MHz).

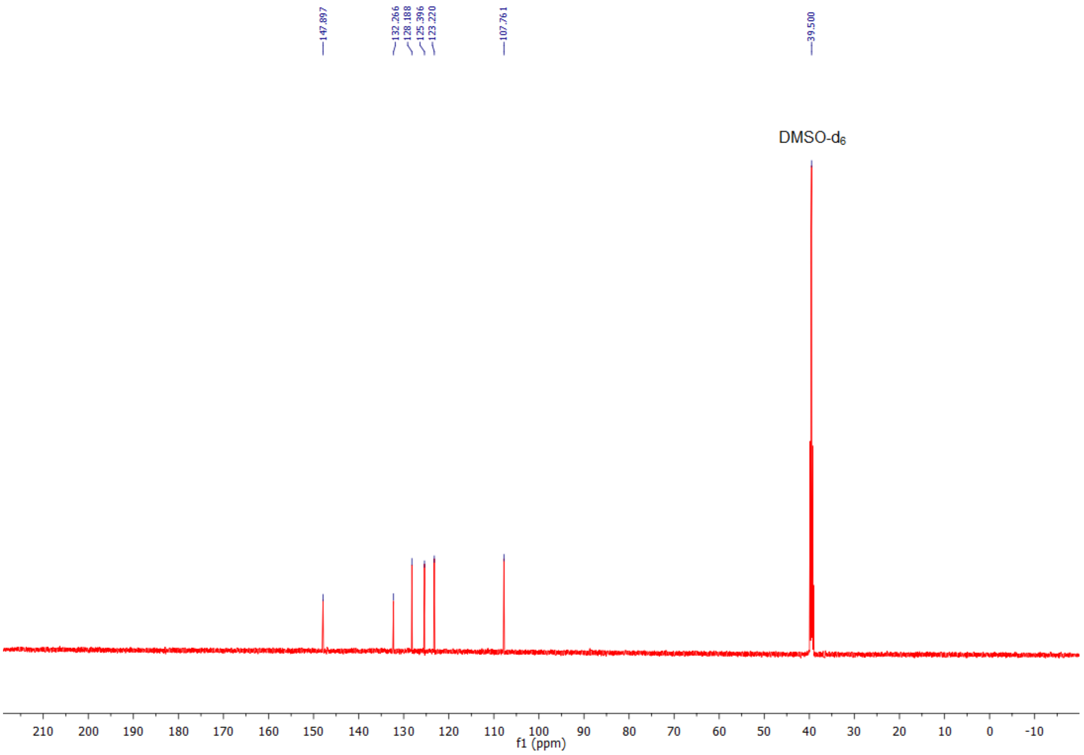

^{13}C NMR of **2l** in DMSO-d_6 at T = 298 K (150 MHz).

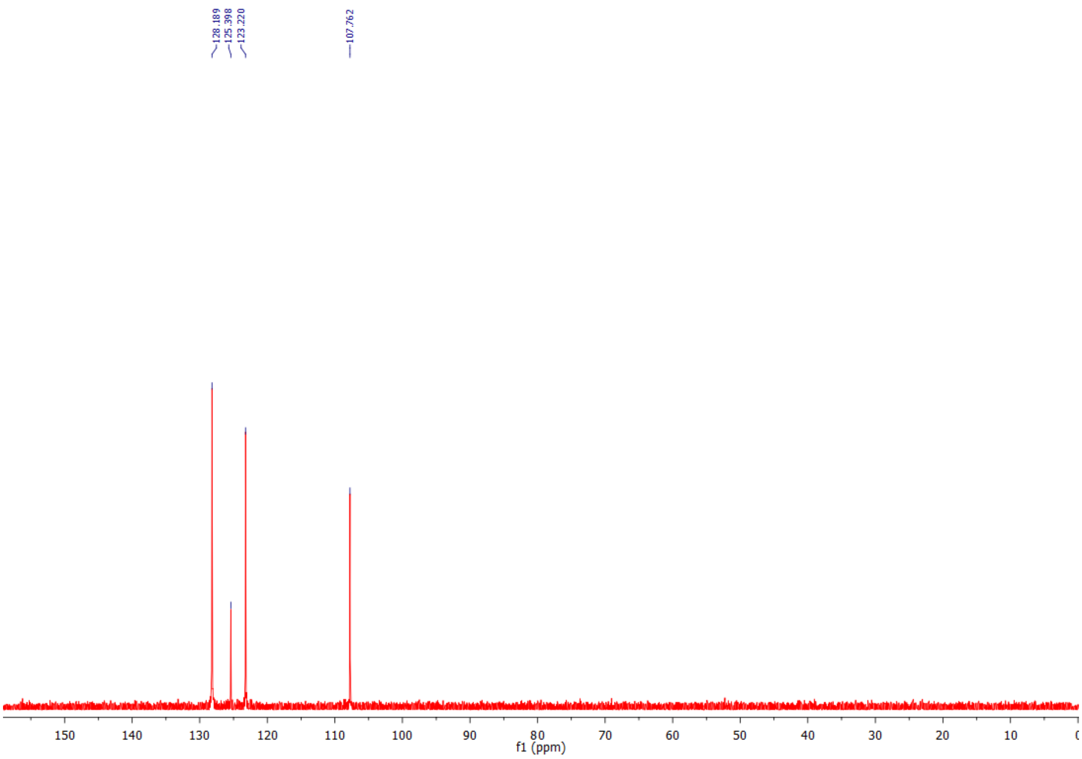

DEPT-135 NMR of **2l** in DMSO-d_6 at T = 298 K (150 MHz).

2,5-Bis(thiophen-3-yl)furan (2m)

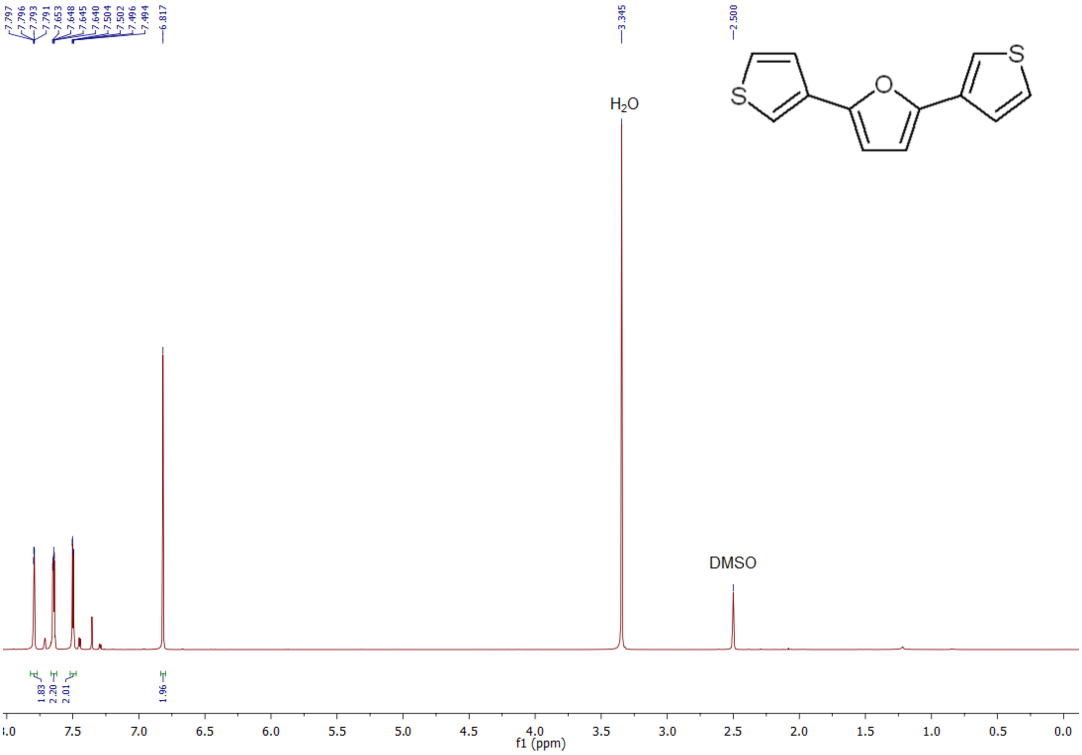

^{1}H NMR of **2m** in DMSO-d_6 at T = 298 K (600 MHz).

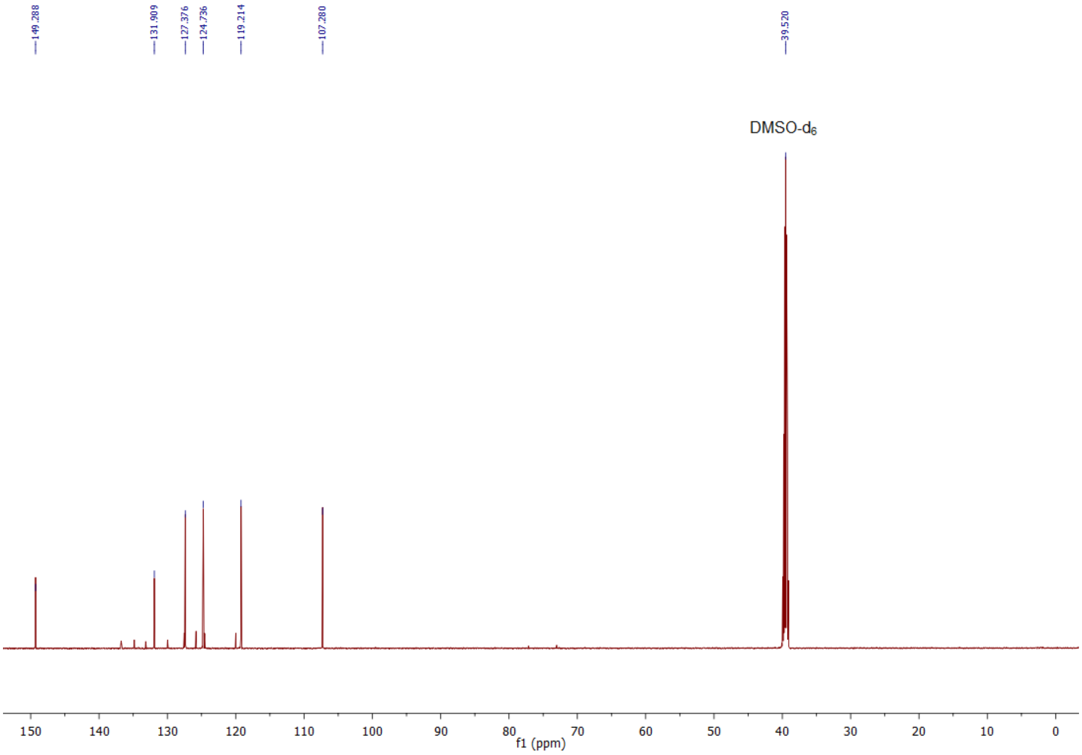

^{13}C NMR of **2m** in DMSO-d_6 at T = 298 K (150 MHz).

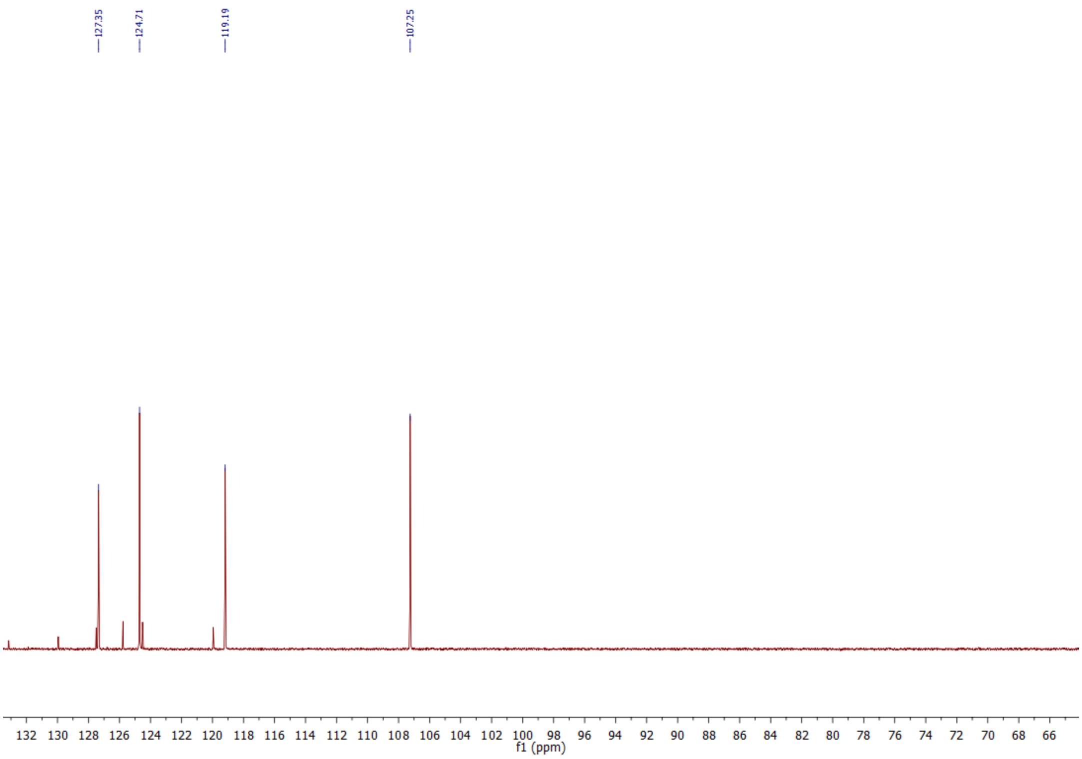

DEPT-135 NMR of **2m** in DMSO-d_6 at T = 298 K (150 MHz).

2,5-Bis(phenanthren-9-yl)furan (2n)

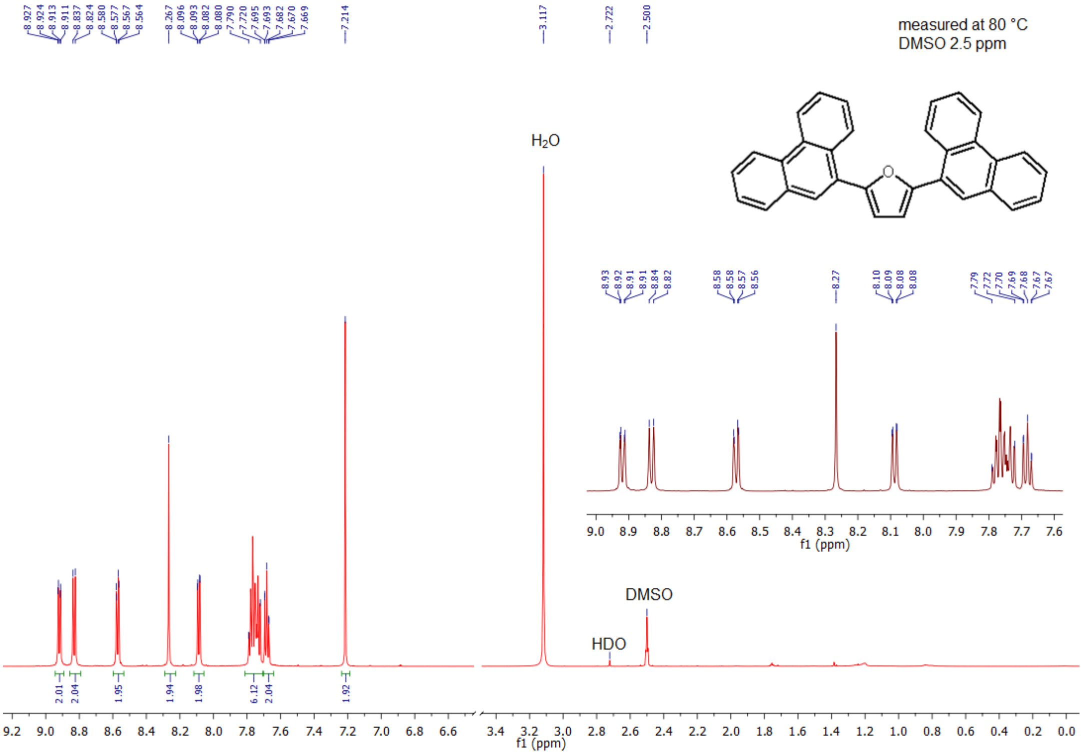

^{1}H NMR of **2n** in DMSO-d_6 at T = 378 K (600 MHz).

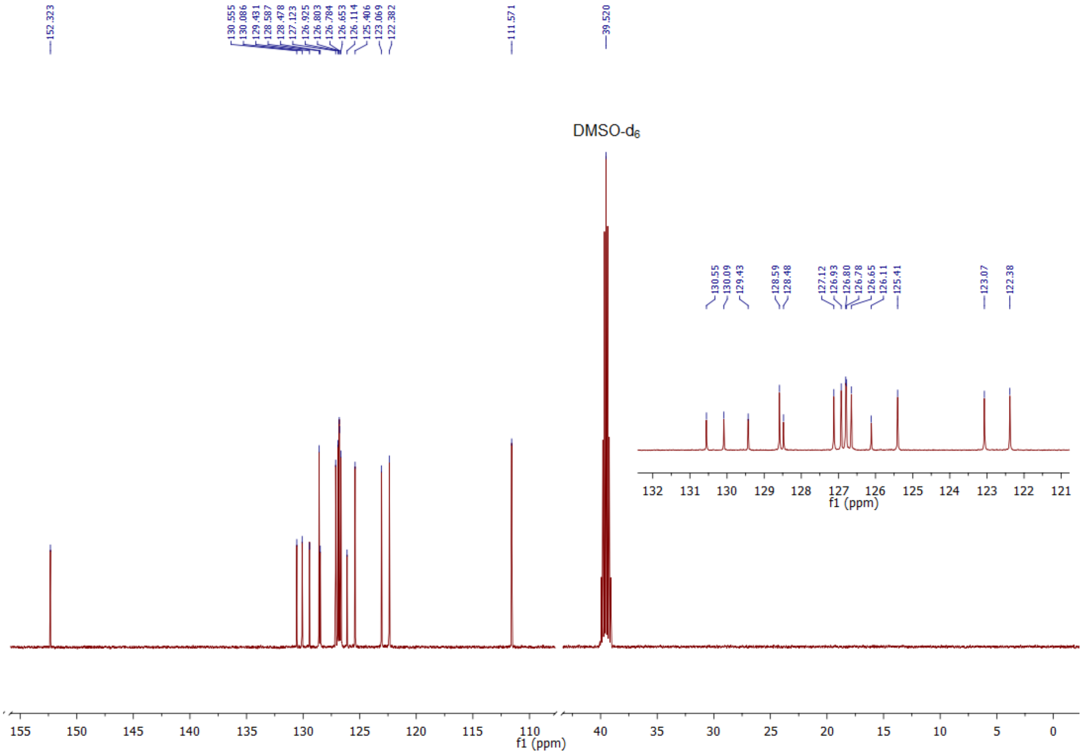

^{13}C NMR of **2n** in DMSO-d_6 at T = 378 K (150 MHz).

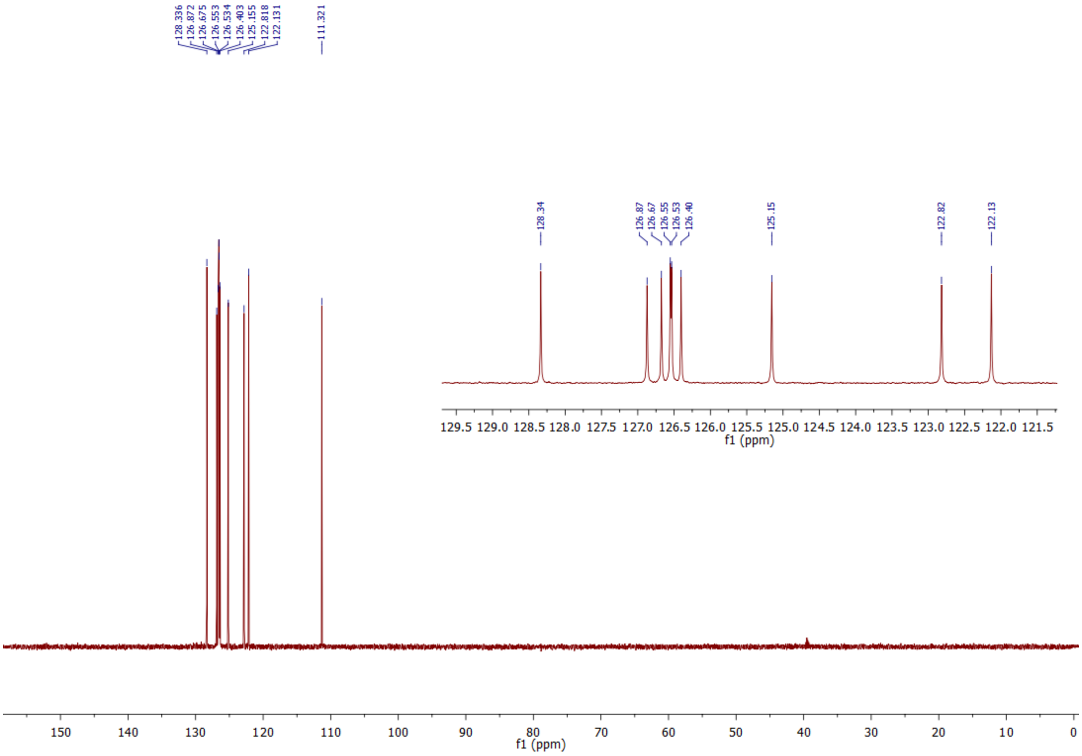

DEPT-135 NMR of **2n** in DMSO-d_6 at T = 378 K (150 MHz).

2,5-Bis(4-fluorophenyl)furan (2o)

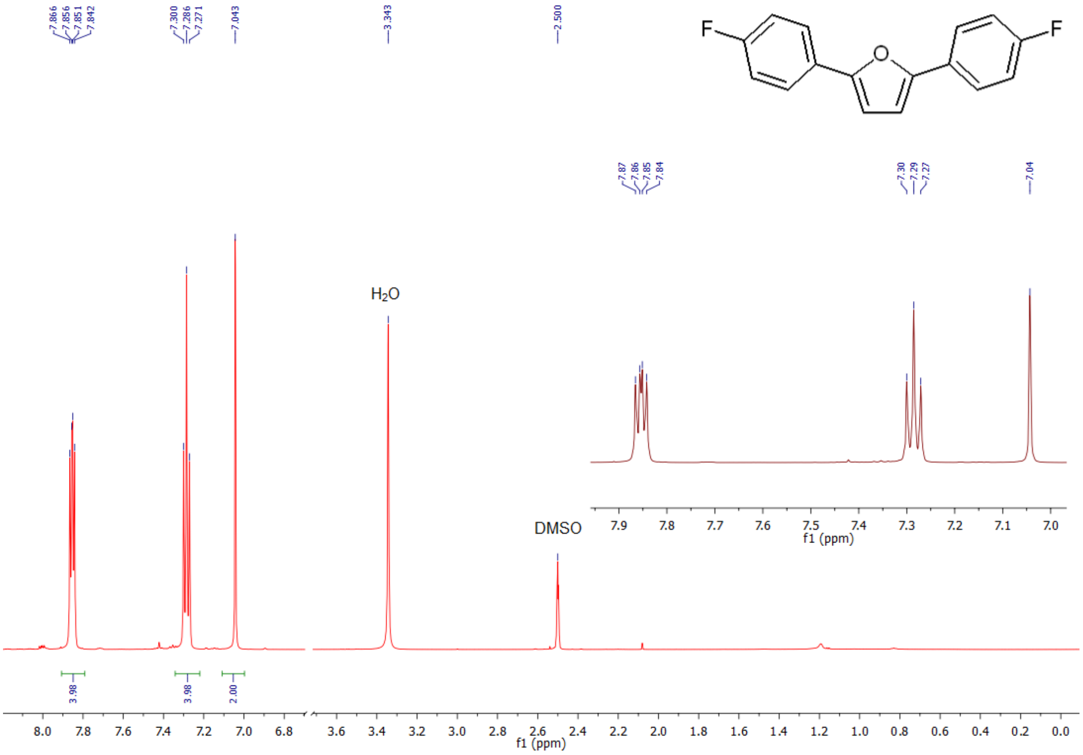

^{1}H NMR of **2o** in DMSO-d_6 at T = 298 K (600 MHz).

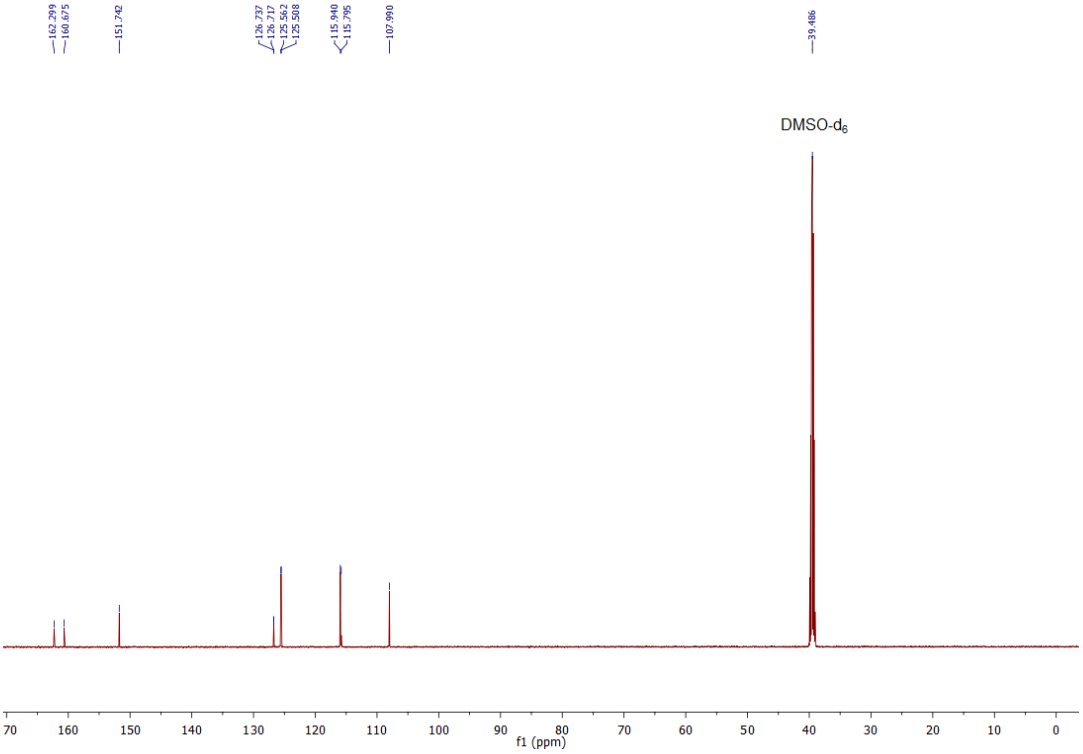

^{13}C NMR of **2o** in DMSO-d_6 at T = 298 K (150 MHz).

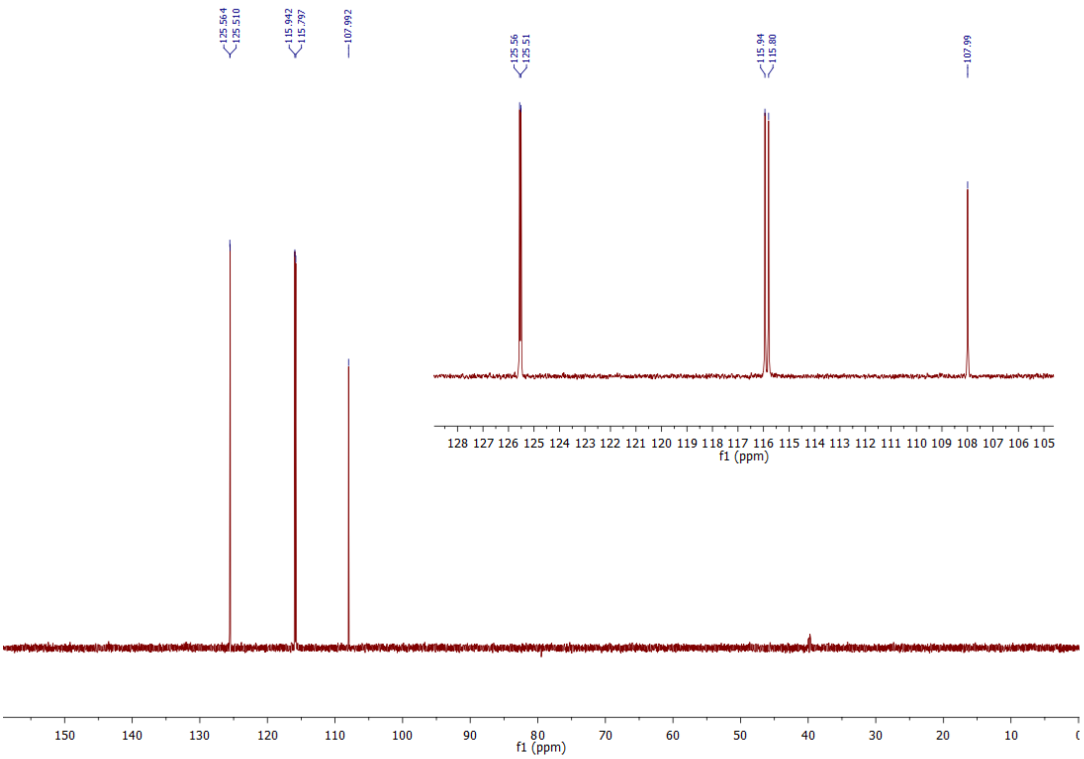

DEPT-135 NMR of **2o** in DMSO-d_6 at T = 298 K (150 MHz).

7. CV data of compounds 2a–2o

The oxidation potentials were determined with $E_{1/2}(Ag/Ag^{+}) = \frac{(E_{pa}-E_{pc})}{2}$ ($[FeCp_2]/[FeCp_2]^+$ ($E_0^{0/+1}$ = 450 mV) [4]). The half-wave potential against NHE were calculated with

$E_{1/2}(H_2/H^{+}) = E_{1/2}(Ag/Ag^{+}) + NHE$ (NHE = 0.198 V)

The HOMO energy of all compounds were calculated with the first oxidation potential (vs NHE) of the corresponding molecule.

$$E_{HOMO} = -\left(E_{\frac{1}{2}}(Ox.1) - E_{\frac{Fc}{Fc+}}\right) - 4.6\ eV$$

The summand 4.6 eV is the ferrocene HOMO energy value related to the vacuum level [18].

2,5-Diphenylfuran (2a)

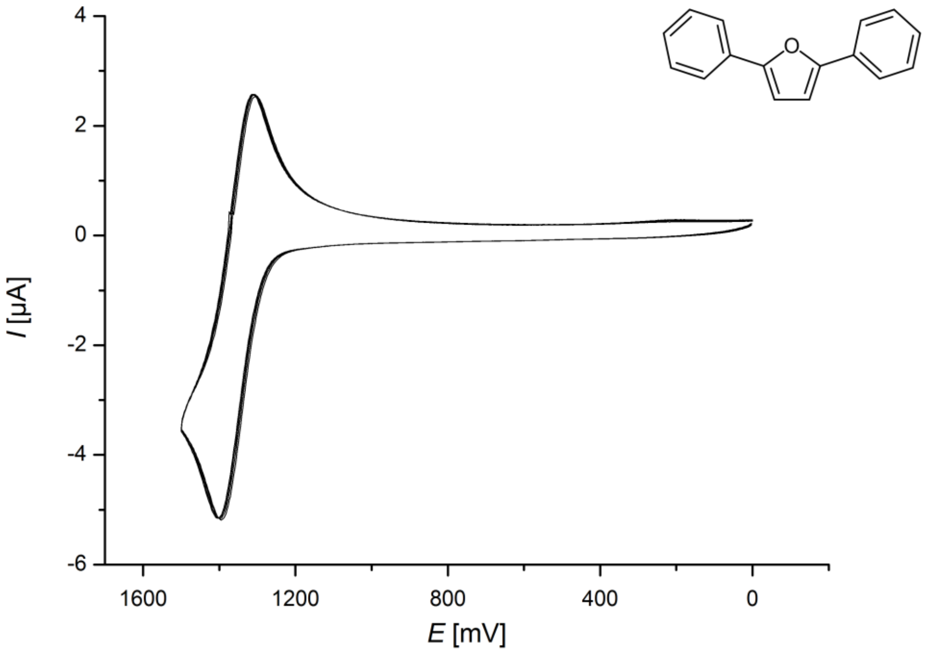

Recorded in dichloromethane, T = 293 K, ν = 100 mV/s, 0.1 M electrolyt: $[Bu_4N][PF_6]$, Pt working electrode, Pt counter electrode, Ag/AgCl (in KCl) reference electrode.

2,5-Di-*o*-tolylfuran (2b)

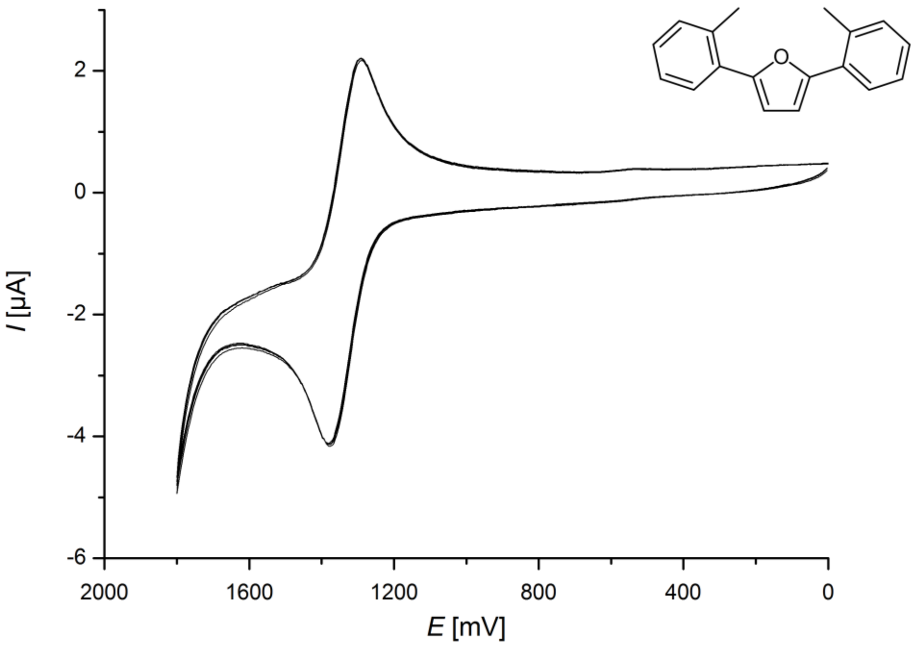

Recorded in dichloromethane, *T* = 293 K, ν = 100 mV/s, 0.1 M electrolyt: $[Bu_4N][PF_6]$, Pt working electrode, Pt counter electrode, Ag/AgCl (in KCl) reference electrode.

2,5-Di-*m*-tolylfuran (2c)

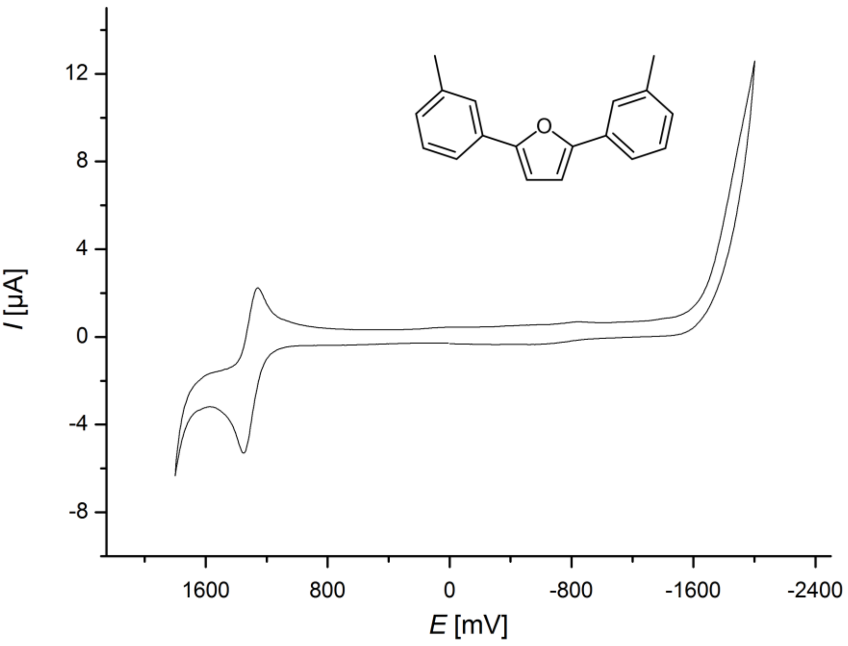

Recorded in dichloromethane, *T* = 293 K, ν = 100 mV/s, 0.1 M electrolyt: $[Bu_4N][PF_6]$, Pt working electrode, Pt counter electrode, Ag/AgCl (in KCl) reference electrode.

2,5-Di-*p*-tolylfuran (2d)

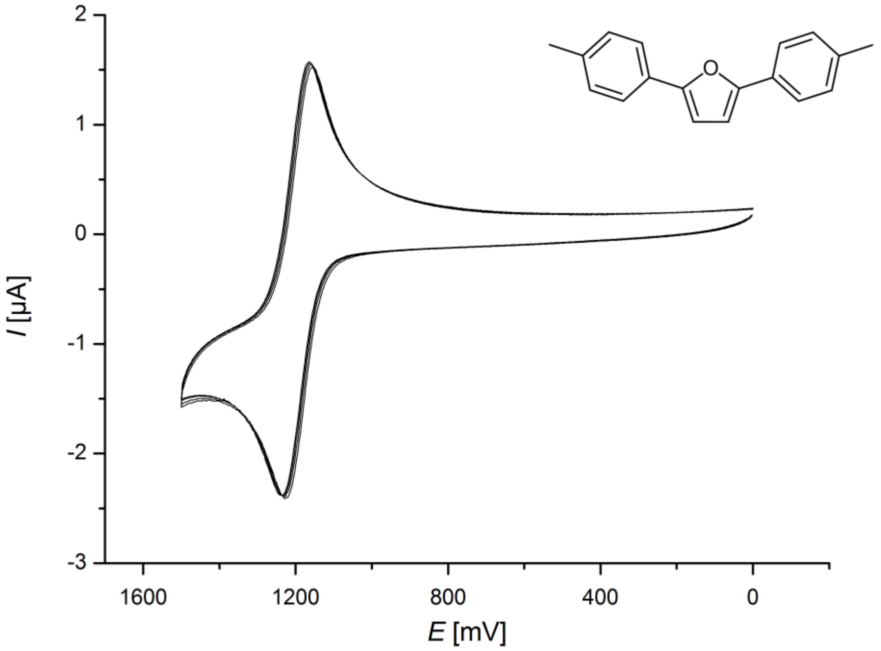

Recorded in dichloromethane, *T* = 293 K, ν = 100 mV/s, 0.1 M electrolyt: $[Bu_4N][PF_6]$, Pt working electrode, Pt counter electrode, Ag/AgCl (in KCl) reference electrode.

2,5-Bis(3,5-dimethylphenyl)furan (2e)

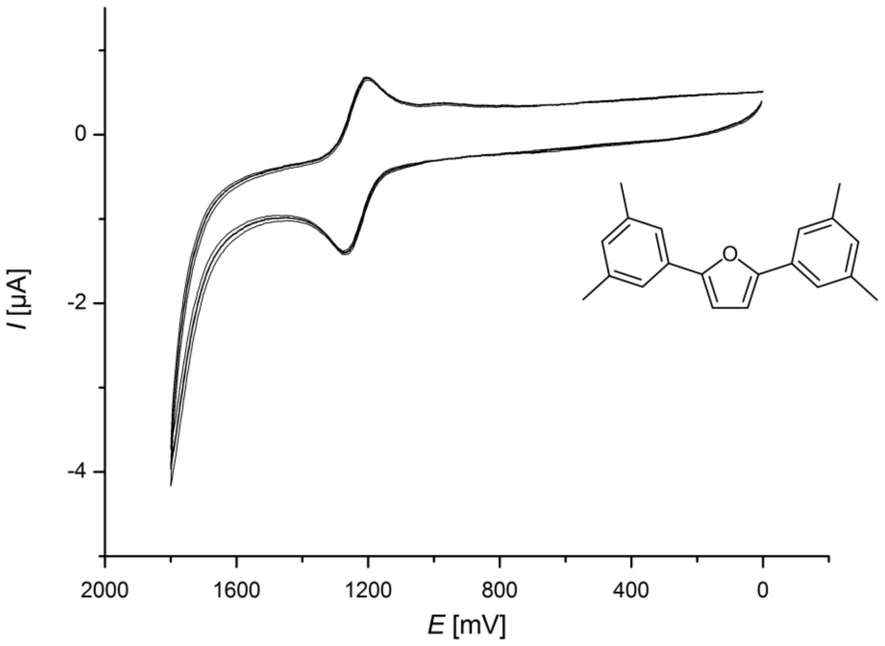

Recorded in dichloromethane, T = 293 K, ν = 100 mV/s, 0.1 M electrolyt: $[Bu_4N][PF_6]$, Pt working electrode, Pt counter electrode, Ag/AgCl (in KCl) reference electrode.

2,5-Bis(2-methoxy-5-methylphenyl)furan (2h)

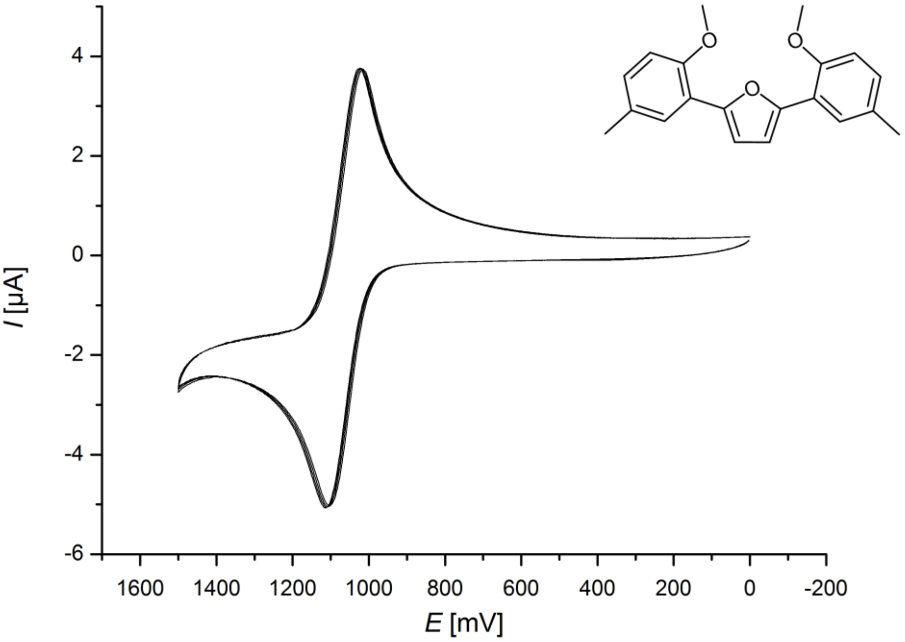

Recorded in dichloromethane, T = 293 K, ν = 100 mV/s, 0.1 M electrolyt: $[Bu_4N][PF_6]$, Pt working electrode, Pt counter electrode, Ag/AgCl (in KCl) reference electrode.

2,5-Di(naphthalen-1-yl)furan (2i)

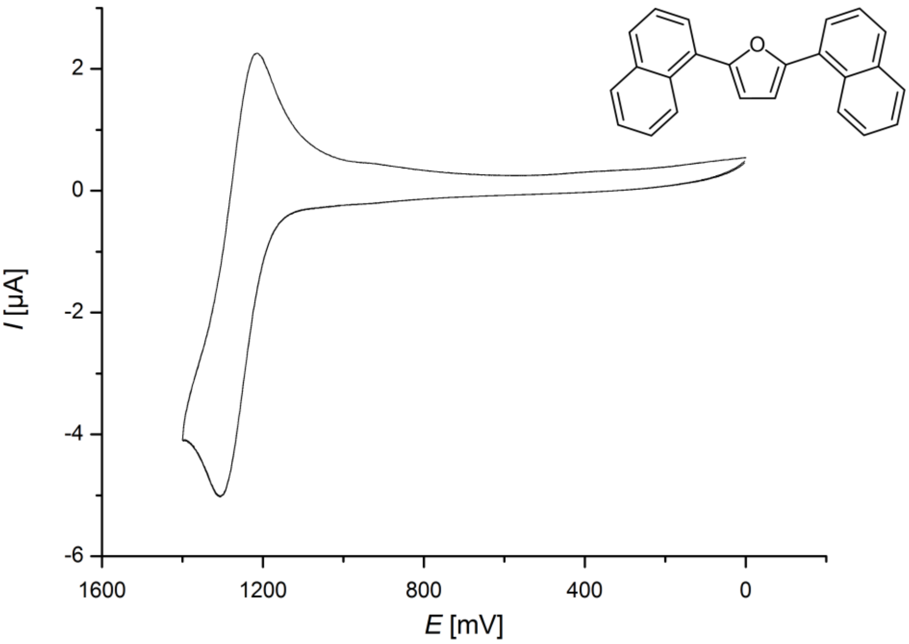

0.1 M

Recorded in dichloromethane, T = 293 K, ν = 100 mV/s, 0.1 M electrolyt: $[Bu_4N][PF_6]$, Pt working electrode, Pt counter electrode, Ag/AgCl (in KCl) reference electrode.

2,5-Di(naphthalen-2-yl)furan (2j)

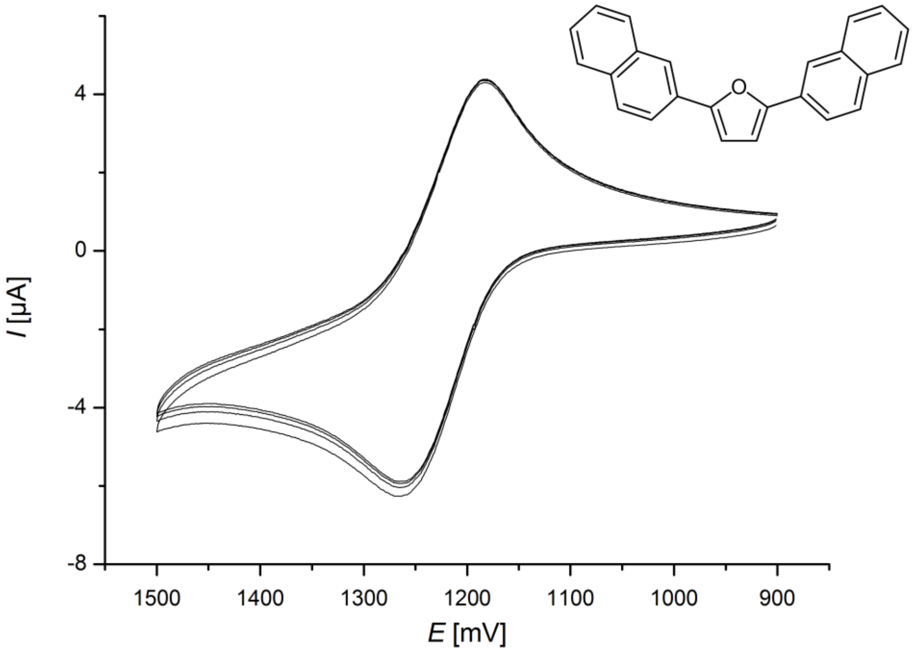

Recorded in dichloromethane, T = 293 K, ν = 100 mV/s, 0.1 M electrolyt: $[Bu_4N][PF_6]$, Pt working electrode, Pt counter electrode, Ag/AgCl (in KCl) reference electrode.

2,5-Bis(3-hydroxymethylphenyl)furan (2k)

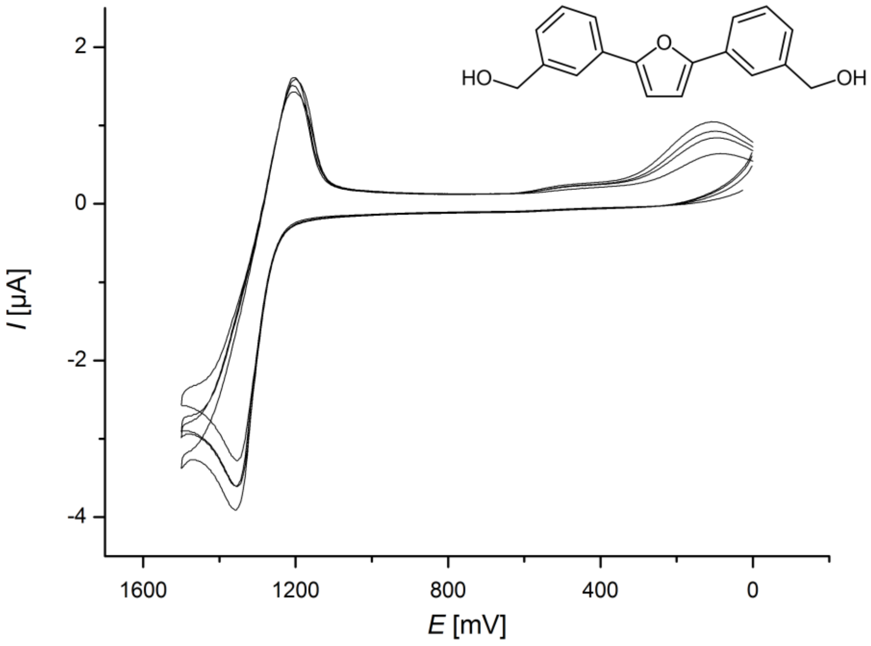

Recorded in dichloromethane, T = 293 K, ν = 100 mV/s, 0.1 M electrolyt: $[Bu_4N][PF_6]$, Pt working electrode, Pt counter electrode, Ag/AgCl (in KCl) reference electrode.

2,5-Di(phenanthren-9-yl)furan (2n)

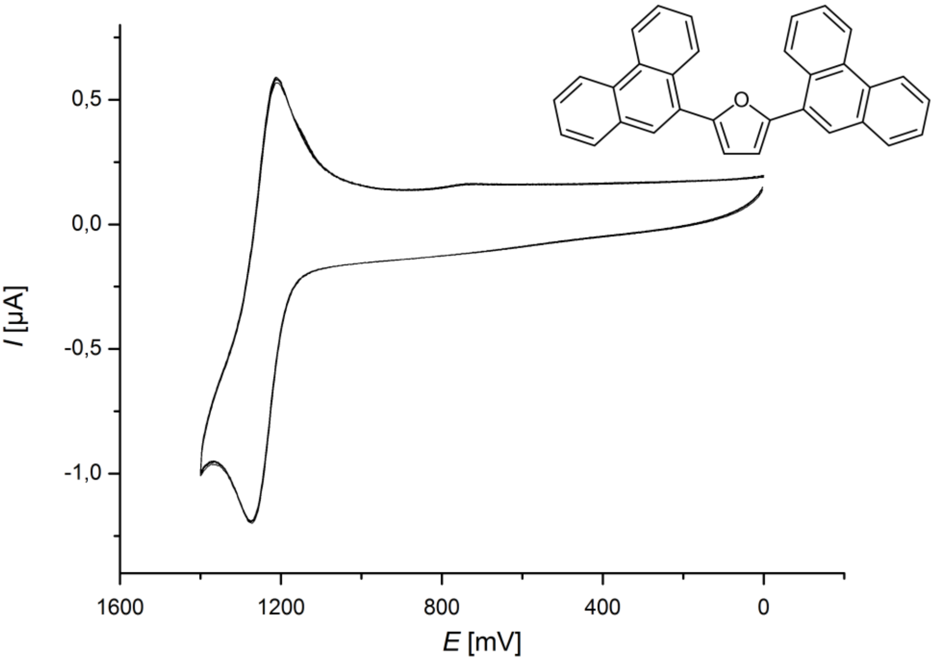

Recorded in dichloromethane, T = 293 K, ν = 100 mV/s, 0.1 M electrolyt: $[Bu_4N][PF_6]$, Pt working electrode, Pt counter electrode, Ag/AgCl (in KCl) reference electrode.

2,2,5-Bis(4-fluorophenyl)furan (2o)

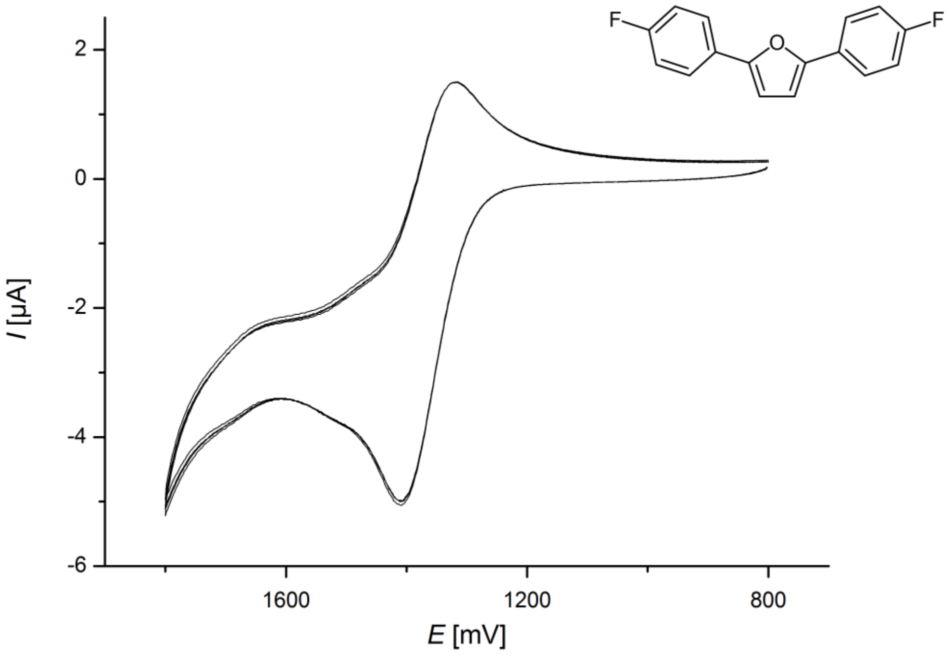

Recorded in dichloromethane, T = 293 K, ν = 100 mV/s, 0.1 M electrolyt: $[Bu_4N][PF_6]$, Pt working electrode, Pt counter electrode, Ag/AgCl (in KCl) reference electrode.

8. xyz-Coordinates and dihedral angles of compounds 2a–2o

The geometries of the furans were optimized in DFT calculations with the B3LYP [5] functional and the 6-311G(d,p) [6-9] basis set as implemented in the program package *Gaussian09* [10]. The minima were confirmed by analytical frequency analysis. To find the global minima the DFT calculation started from the "*trans*" configuration with a dihedral angle of 90° (Fig. 1).

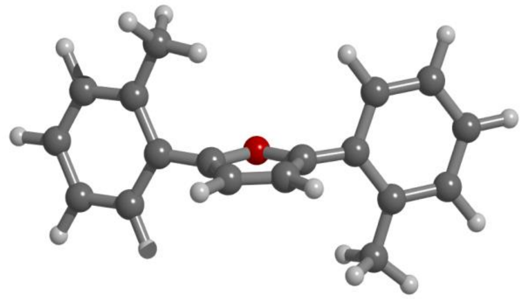

Figure s1: Example of starting configuration.

x,y,z-Coordinates of 2,5-diphenylthiophene (2a)

Dehedral Angle θ = 1°

SCF Done: E(RB3LYP) = -692.322306636 Hartrees A.U. after 8 cycles

Sum of electronic and zero-point Energies= -692.091336 Hartrees

Sum of electronic and thermal Energies= -692.078387 Hartrees

Sum of electronic and thermal Enthalpies= -692.077443 Hartrees

Sum of electronic and thermal Free Energies= -692.133352 Hartrees

C	3.591982185	0.915900996	-0.014631071
C	2.411688379	0.151660635	0.000463657
C	2.519532027	-1.249185169	0.016804460
C	3.768472388	-1.862492877	0.017992581
C	4.932494748	-1.095482053	0.003041257
C	4.836000063	0.296950670	-0.013177591
C	1.110560862	0.805300162	-0.000497609
C	0.710709117	2.117158245	-0.000328752
C	-0.710709551	2.117158113	0.000313276
C	-1.110560798	0.805299982	0.000492960
O	0.000000084	0.002479290	0.000000689
C	-2.411688442	0.151660424	-0.000463602
C	-2.519532095	-1.249185275	-0.016812339
C	-3.768472489	-1.862492851	-0.017997787
C	-4.932494679	-1.095481857	-0.003035744
C	-4.835999936	0.296950689	0.013192171
C	-3.591981878	0.915900906	0.014643333

H	-1.353092973	2.982934659	0.000078964
H	1.353092079	2.982935093	-0.000103642
H	-1.621097925	-1.852719513	-0.029262101
H	-3.831668965	-2.944807726	-0.030786576
H	-5.904167121	-1.575582130	-0.003942727
H	-5.734595580	0.903407783	0.025171722
H	-3.535635328	1.997987383	0.028060365
H	1.621097845	-1.852719497	0.029245613
H	3.831668547	-2.944807853	0.030774658
H	5.904167174	-1.575582384	0.003949970
H	5.734595791	0.903407831	-0.025147849
H	3.535636403	1.997987627	-0.028039117

x,y,z-Coordinates of 2,5-di-*o*-tolylfuran (2b)

Dehedral Angle θ = 35°

SCF Done: E(RB3LYP) = -770.969862564 Hartrees A.U. after 14 cycles

Sum of electronic and zero-point Energies= -770.683129 Hartrees

Sum of electronic and thermal Energies= -770.667350 Hartrees

Sum of electronic and thermal Enthalpies= -770.666406 Hartrees

Sum of electronic and thermal Free Energies= -770.727485 Hartrees

C	-1.720353194	-1.672183815	-1.440860207
C	-2.740051899	-0.893366851	-0.643287162
C	-4.051084075	-1.375850453	-0.568293430
C	-5.053661570	-0.694415826	0.116379128
C	-4.755298602	0.508906453	0.751296045
C	-3.458700147	1.004210112	0.701588651
C	-2.437966700	0.323828019	0.013740586
C	-1.110985759	0.942950688	-0.005822200
O	-0.000011020	0.139447488	0.000020685
C	-0.711675759	2.253545965	-0.003794761
C	0.711651380	2.253546092	0.003852304
C	1.110963349	0.942951493	0.005868312
C	2.437950454	0.323835152	-0.013725313
C	3.458647573	1.004215362	-0.701633715
C	4.755247171	0.508924341	-0.751396986
C	5.053651182	-0.694389642	-0.116480917
C	4.051113179	-1.375822396	0.568247032
C	2.740080279	-0.893344938	0.643304609
C	1.720451737	-1.672155986	1.440974403
H	-1.133381123	-1.020702189	-2.091863685
H	-2.218408527	-2.419587118	-2.060886796
H	-1.010462270	-2.191010679	-0.791519853
H	-4.290584902	-2.305610584	-1.073788606
H	-6.058647170	-1.099564252	0.150227238
H	-5.521845153	1.052243519	1.291775917
H	-3.217514680	1.925503867	1.218863126
H	-1.360565053	3.114705093	-0.018998713
H	1.360539330	3.114706267	0.019056832
H	3.217429503	1.925500633	-1.218907910
H	5.521762495	1.052261993	-1.291920514
H	6.058637274	-1.099532586	-0.150377476
H	4.290642640	-2.305580310	1.073733879
H	1.133363543	-1.020637428	2.091836147
H	2.218584055	-2.419372711	2.061166402
H	1.010666532	-2.191206057	0.791700265

x,y,z-Coordinates of 2,5-di-*m*-tolylfuran (2c)

Dehedral Angle θ = 0°

SCF Done: E(RB3LYP) = -770.977563472 Hartrees A.U. after 12 cycles

Sum of electronic and zero-point Energies= -770.692451 Hartrees

Sum of electronic and thermal Energies= -770.675649 Hartrees

Sum of electronic and thermal Enthalpies= -770.674704 Hartrees

Sum of electronic and thermal Free Energies= -770.741144 Hartrees

C	1.110983230	-0.644280702	-0.000125982
C	0.710806681	-1.956102558	-0.000147759
C	-0.710804099	-1.956102685	-0.000080765
C	-1.110981526	-0.644280676	-0.000014881
O	0.000000042	0.158328282	-0.000045217
C	2.411843528	0.010406776	-0.000191883
C	-2.411840861	0.010406393	0.000130272
C	3.590798961	-0.757342856	0.000051279
C	4.850709003	-0.163490862	-0.000069342
C	4.931309635	1.236831183	-0.000599778
C	3.774587288	2.010156306	-0.000830345
C	2.519061535	1.408985860	-0.000606493
C	-2.519059207	1.408983108	0.000691678
C	-3.774587478	2.010154406	0.000865897
C	-4.931308233	1.236829347	0.000485269
C	-4.850707453	-0.163495313	-0.000092278
C	-3.590799009	-0.757344073	-0.000260008
C	6.106216796	-1.002957090	0.001126753
C	-6.106224521	-1.002950470	-0.000592954
H	1.353110685	-2.821945363	-0.000215290
H	-1.353109018	-2.821945919	-0.000054962
H	3.522753880	-1.839682375	0.000292159
H	5.903727375	1.718378544	-0.000892092
H	3.849059727	3.092039955	-0.001295601
H	1.623944384	2.017155201	-0.000891324
H	-1.623942764	2.017154608	0.001022414
H	-3.849060732	3.092037822	0.001318033
H	-5.903725682	1.718376104	0.000640745
H	-3.522751541	-1.839682999	-0.000707361
H	5.873265883	-2.069158293	-0.007558019
H	6.725908992	-0.785441789	-0.873939451
H	6.715660315	-0.797886302	0.886428409
H	-6.721321186	-0.791410027	0.879209816
H	-6.720297282	-0.791871157	-0.881230330
H	-5.873249055	-2.069180889	-0.000197478

x,y,z-Coordinates of 2,5-di-*p*-tolylfuran (2d)

Dehedral Angle θ = 0°

SCF Done: E(RB3LYP) = -770.977840193 Hartrees A.U. after 6 cycles

Sum of electronic and zero-point Energies= -770.692763 Hartrees

Sum of electronic and thermal Energies= -770.675950 Hartrees

Sum of electronic and thermal Enthalpies= -770.675006 Hartrees

Sum of electronic and thermal Free Energies= -770.741305 Hartrees

C	-1.111313098	1.031636340	0.000299966
C	-0.711063925	2.343253918	0.002919863
C	0.711062977	2.343255214	0.002922216
C	1.111314251	1.031638462	0.000305127
O	0.000001435	0.228595731	-0.001287452
C	-2.411627211	0.377894509	-0.000645323
C	2.411629746	0.377899192	-0.000638660
C	-2.526497081	-1.020521866	-0.005156930
C	-3.777006944	-1.630902680	-0.007954983
C	-4.955902734	-0.879699818	-0.005170390
C	-4.836707869	0.517385518	-0.003195302
C	-3.595421619	1.137131829	-0.000321966
C	2.526500340	-1.020519607	-0.005197873
C	3.777007971	-1.630898135	-0.008021488
C	4.955905549	-0.879692557	-0.005205486
C	4.836710552	0.517389552	-0.003185452
C	3.595421259	1.137136258	-0.000289542
C	6.311576101	-1.541583487	0.012954657
C	-6.311583245	-1.541574010	0.012925553
H	-1.353675931	3.208943933	0.004594250
H	1.353674285	3.208945780	0.004594306
H	-1.631755401	-1.629999116	-0.009013157
H	-3.835583060	-2.714520135	-0.013944739
H	-5.733155689	1.129362208	-0.005622663
H	-3.544100647	2.219789208	-0.000459999
H	1.631758100	-1.629995861	-0.009085225
H	3.835587521	-2.714515235	-0.014070310
H	5.733157700	1.129366220	-0.005604633
H	3.544099509	2.219793743	-0.000398295
H	6.238788969	-2.603335068	-0.230127545
H	6.992370673	-1.073607495	-0.703301760
H	6.776930069	-1.458746569	1.001149389
H	-6.238665989	-2.603564133	-0.229066763
H	-6.777484964	-1.457735593	1.000773150
H	-6.991986914	-1.074319519	-0.704180311

x,y,z-Coordinates of 2,5-bis(3,5-dimethylphenyl)furan (2e)

Dehedral Angle θ = 0°

SCF Done: E(RB3LYP) = -849.632693976 Hartrees A.U. after 5 cycles

Sum of electronic and zero-point Energies= -849.293442 Hartrees

Sum of electronic and thermal Energies= -849.272790 Hartrees

Sum of electronic and thermal Enthalpies= -849.271846 Hartrees

Sum of electronic and thermal Free Energies= -849.348126 Hartrees

C	3.590011991	-1.170443023	0.003474591
C	2.413543768	-0.398686704	-0.000462216
C	2.521046071	0.997648270	-0.006584900
C	3.770997187	1.624282316	-0.007028772
C	4.919280627	0.831899953	-0.005518340
C	4.844081331	-0.568704178	0.000260985
C	1.111204082	-1.051323725	-0.000581867
C	0.710531805	-2.362956797	0.000193292
C	-0.711254793	-2.362692415	0.000530685
C	-1.111451414	-1.050914242	-0.000114268
O	0.000004148	-0.248609725	-0.000792236
C	-2.413665876	-0.397948992	-0.000140404
C	-2.521280856	0.997939370	-0.001176459
C	-3.771771664	1.624599394	-0.001190339
C	-4.919464585	0.832189703	0.000051475
C	-4.844096095	-0.568882055	0.001196618
C	-3.590313447	-1.170209773	0.001050549
C	-3.864881135	3.132567452	-0.003068135
C	-6.106121014	-1.398891930	0.002676726
C	3.866205628	3.131942779	0.010335482
C	6.105970940	-1.399005698	-0.000008032
H	-1.353830895	-3.228338646	0.001080256
H	1.352810483	-3.228826621	0.000342419
H	-1.620959153	1.600565169	-0.001913230
H	-5.894732954	1.309964328	0.000178465
H	-3.523546412	-2.252382125	0.001965750
H	1.620714515	1.600098440	-0.012550120
H	5.894457200	1.310018923	-0.010270565
H	3.523612083	-2.252639220	0.006306820
H	-3.367598019	3.559673151	0.872986615
H	-3.379398179	3.556574243	-0.887271018
H	-4.903890334	3.466652148	0.003149320
H	6.715199138	-1.189337034	-0.884452679
H	6.723393073	-1.177873526	0.875870614
H	5.880459939	-2.466860854	0.007908543
H	-6.718653552	-1.182908639	0.883303259
H	-6.720202665	-1.183707644	-0.877057701

H	-5.880821843	-2.466821097	0.002979796
H	3.166618796	3.582448480	-0.698576861
H	3.621994930	3.528222278	1.001564185
H	4.872905380	3.469897808	-0.242586008

x,y,z-Coordinates of 2,5-bis(3-methoxyphenyl)furan (2f)

Dehedral Angle θ = 0°

SCF Done: E(RB3LYP) = -921.432298309 Hartrees A.U. after 14 cycles

Sum of electronic and zero-point Energies= -921.137070 Hartrees

Sum of electronic and thermal Energies= -921.118846 Hartrees

Sum of electronic and thermal Enthalpies= -921.117902 Hartrees

Sum of electronic and thermal Free Energies= -921.185718 Hartrees

C	1.110023657	-1.602775404	-0.000026077
C	0.710791626	-2.914685633	-0.000049149
C	-0.710791420	-2.914685849	-0.000050669
C	-1.110023911	-1.602775711	-0.000028620
O	-0.000000211	-0.800244219	-0.000015582
C	2.412319761	-0.950870404	-0.000002151
C	-2.412320047	-0.950870716	-0.000007070
C	2.511041842	0.443338448	-0.000024238
C	3.760881254	1.071751619	-0.000002163
C	4.930105196	0.305760631	0.000040259
C	4.825508113	-1.088256041	0.000063021
C	3.592447411	-1.720543079	0.000043799
C	-2.511041911	0.443338177	-0.000022160
C	-3.760881132	1.071751677	-0.000001257
C	-4.930105281	0.305761040	0.000034907
C	-4.825508478	-1.088255649	0.000049904
C	-3.592447956	-1.720543036	0.000030457
O	3.729546467	2.434403513	-0.000026610
O	-3.729545964	2.434403587	-0.000017999
C	-4.967526022	3.143822978	0.000008654
C	4.967526705	3.143822581	-0.000008420
H	1.353696415	-3.780027836	-0.000066552
H	-1.353695722	-3.780028407	-0.000068836
H	1.623325982	1.061726180	-0.000057761
H	5.906424855	0.769903046	0.000056327
H	5.731851956	-1.683297998	0.000097947
H	3.540571158	-2.802011710	0.000066147
H	-1.623325966	1.061725773	-0.000049171
H	-5.906424855	0.769903687	0.000052184
H	-5.731852485	-1.683297416	0.000079153
H	-3.540572306	-2.802011683	0.000046055
H	-4.703133529	4.199429755	-0.000011408
H	-5.556857143	2.917374794	-0.894036858
H	-5.556806448	2.917395982	0.894092924
H	4.703134439	4.199429406	-0.000029432
H	5.556811846	2.917397617	0.894073244
H	5.556852963	2.917372031	-0.894056580

x,y,z-Coordinates of 2,5-bis(3,4,5-trimethoxyphenyl)furan (2g)

Dehedral Angle θ = 2°

SCF Done: E(RB3LYP) = -1379.62157593 Hartrees A.U. after 5 cycles

Sum of electronic and zero-point Energies= -1379.199270 Hartrees

Sum of electronic and thermal Energies= -1379.169269 Hartrees

Sum of electronic and thermal Enthalpies= -1379.168325 Hartrees

Sum of electronic and thermal Free Energies= -1379.263836 Hartrees

C	-3.526309086	-1.573525553	-0.036135968
C	-2.362168021	-0.792149976	0.030830450
C	-2.487667200	0.601080370	0.125471343
C	-3.743350223	1.198844085	0.183198015
C	-4.906280547	0.415043072	0.132883840
C	-4.781100669	-0.979849423	0.014500016
C	-1.051884504	-1.423761127	-0.007272287
C	-0.631686565	-2.728274716	-0.048653439
C	0.789643941	-2.704467265	-0.064724177
C	1.167969973	-1.386596191	-0.032137823
O	0.044814886	-0.602702943	0.002810001
C	2.456723924	-0.709610349	-0.033655344
C	2.531348311	0.686671675	-0.046355098
C	3.772084534	1.322156778	-0.042415497
C	4.955551302	0.578664617	-0.043010835
C	4.882158225	-0.831044945	-0.033830778
C	3.643382216	-1.465829842	-0.031071071
O	3.809055501	2.694965172	0.015703108
C	4.259223153	3.352087074	-1.181203203
O	6.172606125	1.211516112	-0.082350776
C	6.813427565	1.356420740	1.196761382
O	6.080697552	-1.473717532	-0.020182942
C	6.087836256	-2.901442078	-0.004674555
O	-3.814641541	2.558021811	0.364672075
C	-4.352774529	3.308838792	-0.737629028
O	-6.143657815	1.009909894	0.192131966
C	-6.755123267	0.974578984	1.495700851
O	-5.893659730	-1.787382007	0.016085312
C	-6.719676875	-1.704143248	-1.159366835
H	1.445348337	-3.559550435	-0.097822562
H	-1.260722302	-3.603725612	-0.063053305
H	1.637150123	1.294657280	-0.044621424
H	3.589429737	-2.544779813	-0.020579633
H	-1.613312302	1.235602720	0.179059690
H	-3.479215773	-2.652083716	-0.118272140
H	4.221533325	4.420517636	-0.972005252

H	5.281322093	3.059838772	-1.427882247
H	3.592082549	3.122223528	-2.018292106
H	7.755211810	1.871262261	1.010094015
H	6.195449479	1.955985473	1.871202639
H	7.013595169	0.378976994	1.642512189
H	7.136903199	-3.189823556	0.002768520
H	5.596379574	-3.292399861	0.891478964
H	5.604846883	-3.310474849	-0.897222069
H	-7.534782443	-2.410013639	-1.005867315
H	-7.120526177	-0.697189073	-1.286753403
H	-6.148873277	-1.994343222	-2.046940828
H	-7.715681918	1.478296199	1.395911494
H	-6.911217094	-0.057048048	1.819720675
H	-6.135954585	1.506135857	2.223404038
H	-4.313837045	4.354937037	-0.436688954
H	-3.739675893	3.165163628	-1.632994665
H	-5.385140900	3.020801512	-0.941366062

x,y,z-Coordinates of 2,5-bis(2-methoxy-5-methylphenyl)furan (2h)

Dehedral Angle θ = 38°

SCF Done: E(RB3LYP) = -1000.07380556 Hartrees A.U. after 13 cycles

Sum of electronic and zero-point Energies= -999.724094 Hartrees

Sum of electronic and thermal Energies= -999.702128 Hartrees

Sum of electronic and thermal Enthalpies= -999.701184 Hartrees

Sum of electronic and thermal Free Energies= -999.777456 Hartrees

C	-1.105499273	-1.058739701	0.066561518
C	-0.710567636	-2.370706118	0.043137700
C	0.710559455	-2.370708229	-0.043173584
C	1.105495770	-1.058743114	-0.066580551
O	-0.000000243	-0.255463886	-0.000004363
C	-2.436695146	-0.450977649	0.098144071
C	2.436693622	-0.450984140	-0.098151371
C	-3.482973581	-1.093550822	-0.574359995
C	-4.795158965	-0.611950496	-0.572842379
C	-5.045105196	0.570058109	0.121887953
C	-4.028928677	1.240542074	0.804147455
C	-2.726616211	0.739793415	0.809936937
C	3.482965638	-1.093561162	0.574359217
C	4.795151737	-0.611963948	0.572852079
C	5.045107752	0.570040741	-0.121881840
C	4.028938134	1.240527998	-0.804147905
C	2.726622837	0.739785927	-0.809942007
C	-5.890017405	-1.347657558	-1.308992442
C	5.889993697	-1.347646424	1.309051434
O	-1.697863611	1.317217583	1.487605459
O	1.697876618	1.317212090	-1.487618488
C	-1.948103852	2.509588584	2.227509446
C	1.948124416	2.509583700	-2.227518902
H	-1.359740193	-3.230429726	0.093410528
H	1.359729028	-3.230433425	-0.093457145
H	-3.251464051	-1.993344781	-1.134578608
H	-6.047469470	0.985210779	0.136153056
H	-4.266563891	2.149937384	1.338580776
H	3.251450071	-1.993356423	1.134573237
H	6.047474921	0.985186617	-0.136146311
H	4.266581565	2.149917831	-1.338587004
H	-5.619577847	-1.524889043	-2.354096781
H	-6.087325547	-2.324940166	-0.856628330
H	-6.823233268	-0.781252951	-1.295543445
H	6.087005448	-2.325131548	0.857000770
H	6.823325398	-0.781441756	1.295230728
H	5.619715275	-1.524446370	2.354272552

H	-2.688318180	2.343531434	3.016828467
H	-0.995089583	2.780966870	2.677332359
H	-2.285318186	3.321158668	1.575127340
H	2.688341596	2.343525603	-3.016835082
H	0.995113126	2.780966436	-2.677345493
H	2.285338850	3.321151138	-1.575133526

x,y,z-Coordinates of 2,5-bis(naphthalen-1-yl)furan (2i)

Dehedral Angle θ = 42°

SCF Done: E(RB3LYP) = -999.665375672 Hartrees A.U. after 14 cycles

Sum of electronic and zero-point Energies= -999.340809 Hartrees

Sum of electronic and thermal Energies= -999.322733 Hartrees

Sum of electronic and thermal Enthalpies= -999.321789 Hartrees

Sum of electronic and thermal Free Energies= -999.388425 Hartrees

C	-1.103700066	1.591602149	0.138538253
C	-0.706267935	2.901019568	0.087515967
C	0.706265083	2.901019732	-0.087514645
C	1.103697807	1.591602398	-0.138535725
O	-0.000000910	0.786373299	0.000001925
C	-2.418188334	0.981357651	0.348297447
C	2.418186238	0.981358461	-0.348296542
C	-3.295738481	1.636974690	1.199378983
C	-4.592933805	1.148754804	1.447977452
C	-5.016512763	-0.015553697	0.856931147
C	-4.163148216	-0.727507184	-0.024859916
C	-2.845322852	-0.230524319	-0.301810980
C	2.845323350	-0.230522602	0.301811952
C	4.163148221	-0.727504967	0.024857437
C	5.016509746	-0.015552015	-0.856936983
C	-4.603417813	-1.922546534	-0.654313771
C	-3.796218773	-2.598571062	-1.535043733
C	-2.507871760	-2.099687043	-1.829506624
C	-2.045450565	-0.950595243	-1.231476864
C	4.592928466	1.148755693	-1.447983088
C	3.295733660	1.636975129	-1.199381131
C	2.045454513	-0.950593200	1.231481103
C	2.507878745	-2.099683408	1.829511572
C	3.796225282	-2.598566734	1.535045601
C	4.603420707	-1.922543116	0.654311565
H	-1.352120277	3.761868492	0.154590255
H	1.352116843	3.761868995	-0.154590250
H	-2.965480756	2.536496531	1.704835497
H	-5.244457523	1.690713128	2.123646050
H	-6.008489022	-0.407317080	1.054299202
H	6.008485688	-0.407314942	-1.054307352
H	-5.599332065	-2.288475171	-0.428189245
H	-4.145123468	-3.508315280	-2.010202355
H	-1.876878739	-2.627255525	-2.535837727
H	-1.054944370	-0.591624754	-1.468315809
H	5.244449940	1.690713800	-2.123654004
H	2.965474274	2.536496415	-1.704837513

H	1.054948640	-0.591623458	1.468322451
H	1.876888429	-2.627251186	2.535845622
H	4.145132369	-3.508309808	2.010204657
H	5.599334499	-2.288471504	0.428184240

x,y,z-Coordinates of 2,5-bis(naphthalen-2-yl)furan (2j)

Dehedral Angle θ = 0°

SCF Done: E(RB3LYP) = -999.678490130 Hartrees A.U. after 8 cycles

Sum of electronic and zero-point Energies= -999.354343 Hartrees

Sum of electronic and thermal Energies= -999.336142 Hartrees

Sum of electronic and thermal Enthalpies= -999.335198 Hartrees

Sum of electronic and thermal Free Energies= -999.403481 Hartrees

C	-6.250714820	-0.742977875	-0.000281790
C	-4.982644180	-0.109340576	-0.000273362
C	-3.800972926	-0.916387114	0.000294018
C	-3.939548872	-2.330758340	0.000838760
C	-5.183817183	-2.915256535	0.000817033
C	-6.350976927	-2.115138853	0.000248992
C	-4.834663552	1.304675903	-0.000805639
C	-3.596479962	1.888535487	-0.000767675
C	-2.411222041	1.093181250	-0.000190676
C	-2.532637582	-0.286394572	0.000314864
C	-1.110314736	1.741589723	-0.000124214
C	-0.710570922	3.054124661	-0.000084130
C	0.710570996	3.054124598	0.000074696
C	1.110314646	1.741589607	0.000120629
O	-0.000000076	0.938339075	-0.000000127
C	2.411221930	1.093181080	0.000189816
C	3.596479761	1.888535403	0.000772966
C	4.834663415	1.304675956	0.000813352
C	4.982644143	-0.109340507	0.000277694
C	3.800972958	-0.916387156	-0.000295591
C	2.532637545	-0.286394725	-0.000318854
C	3.939549041	-2.330758366	-0.000843803
C	5.183817410	-2.915256440	-0.000819722
C	6.350977033	-2.115138657	-0.000245705
C	6.250714820	-0.742977690	0.000288502
H	1.352303081	3.920235594	0.000122180
H	-1.352302944	3.920235684	-0.000136177
H	3.515190130	2.968739497	0.001209244
H	5.725443040	1.923859931	0.001270553
H	1.643608278	-0.905312413	-0.000776296
H	7.143927072	-0.126994540	0.000725967
H	7.325688946	-2.589520791	-0.000233359
H	5.275964584	-3.995475060	-0.001240055
H	3.044623071	-2.944007161	-0.001288461
H	-1.643608262	-0.905312191	0.000767630
H	-5.725443199	1.923859804	-0.001257940
H	-3.515190570	2.968739608	-0.001200564

H	-3.044622838	-2.944007050	0.001278836
H	-5.275964256	-3.995475160	0.001234703
H	-7.325688735	-2.589521082	0.000238494
H	-7.143927124	-0.126994816	-0.000714705

x,y,z-Coordinates of 2,5-bis(3-hydroxymethylphenyl)furan (2k)

Dehedral Angle θ = 0°

SCF Done: E(RB3LYP) = -921.443480123 Hartrees A.U. after 6 cycles

Sum of electronic and zero-point Energies= -921.147991 Hartrees

Sum of electronic and thermal Energies= -921.129156 Hartrees

Sum of electronic and thermal Enthalpies= -921.128212 Hartrees

Sum of electronic and thermal Free Energies= -921.199242 Hartrees

C	1.110893286	-1.622882733	-0.012304728
C	0.710825694	-2.933885777	-0.052453589
C	-0.710861768	-2.933879105	-0.052305427
C	-1.110908812	-1.622872483	-0.012074658
O	-0.000001496	-0.820334096	0.012763501
C	2.412891124	-0.970787695	0.009323902
C	-2.412896458	-0.970764914	0.009736088
C	2.519677349	0.427338436	0.048584704
C	3.764119050	1.058296489	0.073240212
C	4.924539847	0.283188063	0.044992601
C	4.830529844	-1.108540427	-0.002484937
C	3.592456153	-1.736062125	-0.017138163
C	-2.519669232	0.427390624	0.048013799
C	-3.764099793	1.058361022	0.072835683
C	-4.924531677	0.283226457	0.045735991
C	-4.830540300	-1.108533437	-0.000783115
C	-3.592469880	-1.736061189	-0.015581386
C	3.830353664	2.566016257	0.165284871
C	-3.830310393	2.566142085	0.163854527
O	5.101838633	3.026219925	-0.284388219
O	-5.101832378	3.026060113	-0.286019257
H	1.353313434	-3.799176698	-0.079021073
H	-1.353363796	-3.799162739	-0.078768664
H	1.615933364	1.025483435	0.057564965
H	5.889387155	0.772560711	0.052387866
H	5.733990360	-1.707647009	-0.028288801
H	3.538737840	-2.817545616	-0.053618041
H	-1.615923547	1.025545751	0.056039940
H	-5.889375142	0.772604760	0.053250862
H	-5.734010574	-1.707663614	-0.025691322
H	-3.538764378	-2.817572123	-0.051258154
H	3.020424669	3.000699634	-0.435090022
H	3.660726934	2.866816244	1.208780929
H	-3.660598418	2.867647470	1.207132172
H	-3.020428866	3.000415238	-0.436878519
H	5.180426037	3.953680877	-0.041128865
H	-5.180499344	3.953608888	-0.043119723

x,y,z-Coordinates of 2,5-bis(thiophen-2-yl)furan (2l)

Dehedral Angle θ = 0°

SCF Done: E(RB3LYP) = -1333.84429854 Hartrees A.U. after 15 cycles

Sum of electronic and zero-point Energies= -1333.680751 Hartrees

Sum of electronic and thermal Energies= -1333.668310 Hartrees

Sum of electronic and thermal Enthalpies= -1333.667365 Hartrees

Sum of electronic and thermal Free Energies= -1333.722339 Hartrees

C	1.107968810	-0.561768032	0.000018164
C	0.710839008	-1.874467857	0.000073237
C	-0.710838093	-1.874468089	0.000090448
C	-1.107967963	-0.561768657	0.000037967
O	0.000000780	0.247314469	-0.000006247
C	2.387264675	0.099321220	-0.000018542
C	-2.387264939	0.099320777	0.000039061
C	2.642382293	1.452131545	-0.000089949
C	4.030550039	1.760976250	-0.000099328
C	4.824282497	0.650330307	-0.000034418
C	-2.642382653	1.452131010	0.000171543
C	-4.030550451	1.760975689	0.000124500
C	-4.824283243	0.650329968	-0.000042663
S	-3.885008334	-0.810058040	-0.000160006
S	3.885008049	-0.810058468	0.000039578
H	1.357782032	-2.737397208	0.000103730
H	-1.357781884	-2.737396822	0.000147528
H	1.855475572	2.193362442	-0.000135335
H	4.422110321	2.769565381	-0.000151970
H	5.901351900	0.587011675	-0.000027173
H	-1.855476244	2.193361913	0.000307330
H	-4.422110750	2.769564889	0.000217449
H	-5.901352482	0.587011252	-0.000104858

x,y,z-Coordinates of 2,5-bis(thiophen-3-yl)furan (2m)

Dehedral Angle θ = 0°

SCF Done: E(RB3LYP) = -1333.84469985 Hartrees A.U. after 8 cycles

Sum of electronic and zero-point Energies= -1333.681146 Hartrees

Sum of electronic and thermal Energies= -1333.668795 Hartrees

Sum of electronic and thermal Enthalpies= -1333.667850 Hartrees

Sum of electronic and thermal Free Energies= -1333.722421 Hartrees

C	-1.103830731	0.821119310	0.000001338
C	-0.739201816	2.141184812	0.000012095
C	0.684159978	2.176596088	0.000009821
C	1.113838997	0.875673892	-0.000002036
O	0.026225070	0.042097634	-0.000007305
C	-2.376364475	0.124929598	0.000000000
C	2.426133953	0.262637131	-0.000003217
C	-2.501223100	-1.307147958	0.000006771
C	-3.795507708	-1.729116434	0.000004934
S	-4.903817599	-0.394930723	-0.000004931
C	2.656698584	-1.093129205	0.000003518
S	4.346168167	-1.462339459	0.000002764
C	4.760213852	0.223359105	-0.000006489
C	-3.605147225	0.744528840	-0.000007226
C	3.652574317	1.013486802	-0.000009140
H	-1.407504731	2.987603186	0.000021360
H	1.309396934	3.055134595	0.000016790
H	-1.653620508	-1.977669818	0.000013018
H	-4.170894664	-2.740618796	0.000009142
H	1.928945729	-1.888534731	0.000009038
H	5.796666666	0.523184994	-0.000009332
H	-3.821244871	1.801438420	-0.000013886
H	3.696777996	2.094272107	-0.000015242

x,y,z-Coordinates of 2,5-bis(phenanthren-9-yl)furan (2n)

Dehedral Angle θ = 38°

SCF Done: E(RB3LYP) = -1307.02239560 Hartrees   A.U. after   14 cycles

Sum of electronic and zero-point Energies=   -1306.604522 Hartrees

Sum of electronic and thermal Energies=   -1306.580911 Hartrees

Sum of electronic and thermal Enthalpies=   -1306.579967 Hartrees

Sum of electronic and thermal Free Energies=   -1306.659379 Hartrees

C	-1.109065117	-1.414116570	-0.078129562
C	-0.710388499	-2.722714282	-0.051916387
C	0.710387086	-2.722720744	0.051852160
C	1.109072901	-1.414126973	0.078105457
O	0.000006988	-0.607565344	-0.000000705
C	-2.440756664	-0.802194443	-0.111477480
C	2.440766480	-0.802211377	0.111473948
C	-2.753826175	0.376959139	-0.903926663
C	-4.081967231	0.903389066	-0.881866316
C	-5.113065657	0.250824928	-0.085924948
C	-4.771410740	-0.917381935	0.648822930
C	-3.429977658	-1.403556964	0.618808233
C	-1.788087178	1.003966013	-1.727268929
C	-2.098375181	2.116012979	-2.481804860
C	-3.396124898	2.647735807	-2.443874575
C	-4.361911294	2.048348714	-1.662523244
C	-6.445405071	0.715875630	-0.006156163
C	-7.391298163	0.058657569	0.756360698
C	-7.047454982	-1.098041799	1.478704210
C	-5.755706831	-1.574289859	1.424352467
C	3.429994470	-1.403564706	-0.618810096
C	4.771424197	-0.917379861	-0.648817580
C	5.113065990	0.250829476	0.085933303
C	4.081959981	0.903384039	0.881872148
C	2.753825117	0.376939022	0.903931864
C	4.361887809	2.048351360	1.662524488
C	3.396094312	2.647724769	2.443877596
C	2.098353819	2.115979995	2.481815730
C	1.788080076	1.003929019	1.727279650
C	5.755728528	-1.574276100	-1.424347016
C	7.047472710	-1.098016425	-1.478693214
C	7.391303402	0.058683338	-0.756344267
C	6.445401790	0.715890940	0.006171037
H	-1.358256238	-3.582655906	-0.111484521
H	1.358248343	-3.582668845	0.111393320
H	-3.192093593	-2.270244912	1.225135112
H	-0.784401332	0.607120073	-1.760046496

H	-1.338961206	2.575652285	-3.103886688
H	-3.644044719	3.523749770	-3.032266883
H	-5.358579919	2.468529884	-1.660418882
H	-6.745643855	1.603548278	-0.546771828
H	-8.405436779	0.439296399	0.797683018
H	-7.794841505	-1.608451177	2.075088224
H	-5.474014960	-2.462951988	1.979051361
H	3.192118100	-2.270250712	-1.225142563
H	5.358548169	2.468552739	1.660414501
H	3.644002179	3.523744753	3.032265957
H	1.338935663	2.575605971	3.103902299
H	0.784400406	0.607067531	1.760060398
H	5.474045600	-2.462938070	-1.979050568
H	7.794865635	-1.608417193	-2.075076582
H	8.405439001	0.439330770	-0.797661470
H	6.745631472	1.603564455	0.546790228

x,y,z-Coordinates of 2,5-bis(4-fluorophenyl)furan (2o)

Dehedral Angle θ = 0°

SCF Done: E(RB3LYP) = -890.850618071 Hartrees A.U. after 14 cycles

Sum of electronic and zero-point Energies= -890.636256 Hartrees

Sum of electronic and thermal Energies= -890.621601 Hartrees

Sum of electronic and thermal Enthalpies= -890.620657 Hartrees

Sum of electronic and thermal Free Energies= -890.680900 Hartrees

C	1.110297046	1.015116434	-0.000125458
C	0.711037312	2.326479563	-0.000075106
C	-0.711037243	2.326479515	0.000093679
C	-1.110297014	1.015116387	0.000132949
O	0.000000026	0.211687556	0.000000616
C	2.409902385	0.359205553	0.000067662
C	-2.409902353	0.359205561	-0.000065515
C	2.514431221	-1.041903766	0.004344871
C	3.756994925	-1.666415564	0.004525155
C	4.898594770	-0.880894557	0.000485984
C	-2.514431221	-1.041903782	-0.004330061
C	-3.756994920	-1.666415595	-0.004514517
C	-4.898594780	-0.880894578	-0.000492188
C	-4.837860586	0.505409817	0.003708940
C	-3.592430546	1.119843055	0.003943672
F	-6.112870561	-1.483999806	-0.000642632
C	4.837860575	0.505409783	-0.003728607
C	3.592430504	1.119843002	-0.003959550
F	6.112870508	-1.483999811	0.000632504
H	1.352818421	3.192697787	-0.000033484
H	-1.352818463	3.192697617	0.000061502
H	1.616624188	-1.645539769	0.007754107
H	3.845080729	-2.745677617	0.007800902
H	-1.616624194	-1.645539859	-0.007725717
H	-3.845080654	-2.745677691	-0.007780283
H	-5.752464405	1.084983959	0.006855842
H	-3.542859118	2.201741494	0.007399225
H	5.752464405	1.084983864	-0.006889551
H	3.542858854	2.201741357	-0.007427788

References

[1] Rhys, A. T. R.; Winfield, S. A.; J. N. Miller *Analyst* **1983**, *108*, 1067-1071. DOI: 10.1039/AN9830801067

[2] Pavlopoulos, T. G.; Hammond, P. R. *J. Am. Chem. Soc.* **1974**, *96*, 6568-6579. DOI: 10.1021/ja00828a005

[3] Jones II, G.; Jackson, W. R.; Choi C.; Bergmark W. R. *J. Phys. Chem.* **1985**, *89*, 294-300. DOI: 10.1021/j100248a024

[4] Marcus, R. A. *J. Chem. Phys.* **1956**, *24*, 966-978. DOI: 10.1063/1.1742723

[5] Lee, C.; Yang, W.; Parr, R. G. *Phys. Rev. B* **1988**, *37*, 785-789. DOI: 10.1103/PhysRevB.37.785

[6] Becke, A. D. *J. Chem. Phys.* **1993**, *98*, 1372-1377. DOI: 10.1063/1.464913

[7] Kim, K.; Jordan, K. D. *J. Phys. Chem.* **1994**, *98*, 10089-10094. DOI: 10.1021/j100091a024

[8] Stephens, P. J.; Devlin, F. J.; Chabalowski, C. F.; Frisch, M. J. *J. Phys. Chem.* **1994**, *98*, 11623-11627. DOI: 10.1021/j100096a001

[9] Krishnan, R.; Binkley, J. S.; Seeger, R.; Pople, J. A. *J. Chem. Phys.* **1980**, *72*, 650-654. DOI: 10.1063/1.438955

[10] Frisch, M. J.; Trucks, G. W.; Schlegel, H. B.; Scuseria, G. E.; Robb, M. A.; Cheeseman, J. R.; Scalmani, G.; Barone, V.; Mennucci, B.; Petersson, G. A.; Nakatsuji, H.; Caricato, M.; Li, X.;. Hratchian, H. P; Izmaylov, A. F.; Bloino, J.; Zheng, G.; Sonnenberg, J. L.; Hada, M.; Ehara, M.; Toyota, K.; Fukuda, R.; Hasegawa, J.; Ishida, M.; Nakajima, T.; Honda, Y.; Kitao, O.; Nakai, H.; Vreven, T.; Montgomery Jr., J. A.; Peralta, J. E.; Ogliaro, F.; Bearpark, M.; Heyd, J. J.; Brothers, E.; Kudin, K. N.; Staroverov, V. N.; Kobayashi, R.; Normand, J.; Raghavachari, K.; Rendell, A.; Burant, J. C.; Iyengar, S. S.; Tomasi, J.; Cossi, M.; Rega, N.; Millam, J. M.; Klene, M.; Knox, J. E.; Cross, J. B.; Bakken, V.; Adamo, C.; Jaramillo, J.; Gomperts, R.; Stratmann, R. E.; Yazyev, O.; Austin, A. J.; Cammi, R.; Pomelli, C.; Ochterski, J. W.; Martin, R. L.; Morokuma, K.; Zakrzewski, V. G.; Voth, G. A.; Salvador, P.; Dannenberg, J. J.; Dapprich, S.; Daniels, A. D.; Farkas, O.; Foresman, J. B.; Ortiz, J. V.; Cioslowski, J.; Fox, D. J. GAUSSIAN 09 (Revision A.02) Gaussian, Inc., Wallingford CT, **2009**.

[11] Urselmann, D.; Antovic, D.; Müller, T. J. J. *Beilstein J. Org. Chem.* **2011**, *7*, 1499–1503. DOI: 10.3762/bjoc.7.174

[12] Zhang, M.; Jiang, H.-F.; Neumann, H.; Beller, M.; Dixneuf, P. H. *Angew. Chem., Int. Ed.* **2009**, *48*, 1681-1684. DOI: 10.1002/anie.200805531

[13] De Oliveira, R. B.; Vaz, A. BM; Alves, R. O.; Liarte, D. B.; Donnici, C. L.; Romanha, A. J.; Zani, C. L. *Mem. Inst. Oswaldo Cruz* ***2006***, *101*, 169-173. DOI: 10.1590/S0074-02762006000200009

[14] Majumder, P.; Saha, S. *Phytochemistry* **1978**, *77*, 1439-1440. DOI: 10.1016/S0031-9422(00)94610-7

[15] Yin, G.; Wang, Z.; Chem, A.; Gao, M.; Wu, A.; Pan, Y. *J. Org. Chem.* **2008**, *73*, 3377-3383. DOI: 10.1021/jo702585s

[16] Kagan, J.; Arora, S. K. *Heterocycles* **1983**, *20*, 1941-1943. DOI: 10.3987/R-1983-10-1941

[17] Moriarty, R. M.; Prakash, O.; Duncan, M. P. *Synth. Commun.* **1985**, *15*, 789-795. DOI: 10.1080/00397918508063874

[18] Pommerehne, J.; Vestweber, H.; Guss, W.; Mahrt, R. F.; Bässler, H.; Porsch, M.; Daub, J. *Adv. Mat.* **1995**, 7. DOI: 10.1002/adma.19950070608
